# Potential Dental Biofilm Inhibitors: Dynamic Combinatorial Chemistry Affords Sugar‐Based Molecules that Target Bacterial Glucosyltransferase

**DOI:** 10.1002/cmdc.202000222

**Published:** 2020-07-09

**Authors:** Alwin M. Hartman, Varsha R. Jumde, Walid A. M. Elgaher, Evelien M. Te Poele, Lubbert Dijkhuizen, Anna K. H. Hirsch

**Affiliations:** ^1^ Department of Drug Design and Optimization Helmholtz Institute for Pharmaceutical Research Saarland (HIPS) Helmholtz Centre for Infection Research (HZI) Campus Building E8.1 66123 Saarbrücken Germany; ^2^ Department of Pharmacy Saarland University Campus Building E8.1 66123 Saarbrücken Germany; ^3^ Stratingh Institute for Chemistry University of Groningen Nijenborgh 7 9747 AG Groningen The Netherlands; ^4^ Microbiology, Groningen Biomolecular Sciences and Biotechnology Institute (GBB) University of Groningen Nijenborgh 7 9747 AG Groningen (The Netherlands; ^5^ CarbExplore Research BV Zernikepark 1 9747 AN Groningen (The Netherlands

**Keywords:** drug discovery, dynamic combinatorial chemistry, glucosyltransferase, glycosides, synthesis

## Abstract

We applied dynamic combinatorial chemistry (DCC) to find novel ligands of the bacterial virulence factor glucosyltransferase (GTF) 180. GTFs are the major producers of extracellular polysaccharides, which are important factors in the initiation and development of cariogenic dental biofilms. Following a structure‐based strategy, we designed a series of 36 glucose‐ and maltose‐based acylhydrazones as substrate mimics. Synthesis of the required mono‐ and disaccharide‐based aldehydes set the stage for DCC experiments. Analysis of the dynamic combinatorial libraries (DCLs) by UPLC‐MS revealed major amplification of four compounds in the presence of GTF180. Moreover, we found that derivatives of the glucose‐acceptor maltose at the C1‐hydroxy group act as glucose‐donors and are cleaved by GTF180. The synthesized hits display medium to low binding affinity (*K*_D_ values of 0.4–10.0 mm) according to surface plasmon resonance. In addition, they were investigated for inhibitory activity in GTF‐activity assays. The early‐stage DCC study reveals that careful design of DCLs opens up easy access to a broad class of novel compounds that can be developed further as potential inhibitors.

## Graphical abstract

**Delay decay**: Structure‐based design in combination with acylhydrazone‐based dynamic combinatorial chemistry (DCC) afforded inhibitors of glucansucrase (GS), the main virulence factor responsible for dental caries. DCC offered a facile pathway for finding the first hits of GS in the form of glucose‐ and maltose‐based acylhydrazones, mimicking the GS substrate.

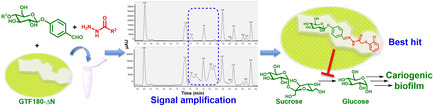

## Introduction

Cariogenic dental biofilm, also known as dental plaque, is a causative agent for dental caries. An important factor for the initiation and development of this oral disease is the fermentation of dietary carbohydrates, of which sucrose is considered the most cariogenic. It acts as a substrate for the synthesis of extracellular (EPS) and intracellular (IPS) polysaccharides, which are involved in the formation of the biofilm, having α‐glucan as one of the main components. The biofilm hosts bacteria and can promote their adhesion to the tooth enamel. Glucosyltransferases (GTFs) are the major producers of EPS, and are secreted by different strains of bacteria. These GTFs, also known as glucansucrases (GSs), therefore are potential targets in order to inhibit biofilm formation and therefore prevent dental caries.[^1^, ^2^, ^3^]

**Figure 1:**
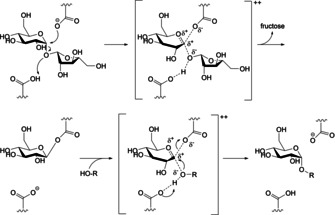
Reaction scheme of the proposed catalytic mechanism of GTF180: α‐retaining double displacement leads to retention of the α‐configuration.^[5]^

Glucansucrases are enzymes that are part of the glycoside hydrolase family GH70, consisting of four catalytically essential conserved sequences. To the superfamily of GH‐H also belong the glycoside hydrolase families 13 and 77.^[4]^ Cocrystal structures, containing the catalytic and C‐terminal domains of glucansucrase of *Lactobacillus reuteri* 180 were previously reported and provided evidence for an α‐retaining double displacement mechanism using one nucleophilic residue.^[5]^ Briefly, the α(1→2)‐glycosidic linkage of a donor substrate (e. g., sucrose) is cleaved, resulting in the release of fructose and formation of a β‐glucosyl−enzyme intermediate. Subsequently, an acceptor substrate attacks the β‐glucosyl−enzyme intermediate, and the glucosyl moiety is transferred to the acceptor (e. g., a glucan chain, maltose or water molecule) with restoration of the α‐anomeric configuration (Figure 1).

Reportedly, glucansucrases can be inhibited by natural as well as synthetic compounds. Natural inhibitors can for example be found in culture broths of bacteria, as was the case for acarbose (Table 1). In 1977, researchers from Bayer discovered α‐amylase inhibitors from broths of *Actinoplanes* strains SE 50, SE 82 and SB 18, of which BAY g 5421 (acarbose) was the most potent. They postulated that acarbose could be a transition‐state analogue.^[6]^ Since then, acarbose has been used as an antidiabetic drug throughout the world, and was found to have cardiovascular benefits.[^7^, ^8^, ^9^] Newbrun et al. showed that acarbose also inhibits GSs,^[10]^ and its mode of action was confirmed by a cocrystal structure.^[11]^ Compounds from plant sources such as polyphenols in green tea extracts,^[12]^ theaflavins in black tea extracts,^[13]^ curcumin^[14]^ and oxyresveratrol^[15]^ showed marked inhibitory effects on the biofilm of the cariogenic pathogen *Streptococcus mutans*, however, high concentrations and sufficient time were required. Their antibiofilm activities were mainly exerted through direct inhibition of the bacterial GTFs or down‐regulation of GTF expression.[^12^, ^13^, ^14^, ^15^] On the other hand, small‐molecule GTF inhibitors can be categorized into just two groups, hydroxychalcones and compounds with a high number of heteroatoms, especially nitrogen.^[16]^ In view of this limited number of inhibitors, we were interested in discovering a novel chemical class that addresses the promising yet underexploited antibiofilm target GTF180.

**Table 1:** Structures, amplification factors, binding affinities and maximum responses of the hits from DCLs 1–4, GTF substrate and cleavage compounds.

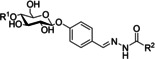						
DCL	Compound	Structure		Amplification fold^[a]^	*K*_D_ [mm] ^[b]^	*R*_max_ (RU)^[c]^
		R^1^	R^2^			
DCL1	**A1H2** ^[d]^	H	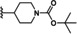	1.8	1.6±0.4	5±1
DCL2	**A2H6**	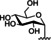	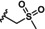	1.5	n.d.	n.d.
DCL3	**A1H12**	H	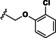	2.1	0.4±0.1	11±2
DCL4	**A2H12**	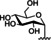	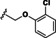	3.2	10±2	140±30
DCL2	**A2H2** (GTF substrate)	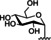	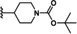	–	5±1	20±2
DCL2	**A1H8** (cleavage product of **A2H8**)	H	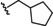	–	8±1	34±5
Acarbose (positive control)		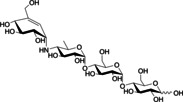		–	0.18±0.01	4±1

[a] Calculated as (%*P*/%*B*), where %*P* and %*B* are the relative peak areas of the compound in the UV chromatograms of the protein‐templated reaction and blank reaction, respectively; [b] *K*_D_: equilibrium dissociation constant determined by SPR; [c] *R*_max_: maximum analyte binding capacity; [d] Compound **A1H2** was also observed in DCL2 as a cleavage product of **A2H2**.

**Figure 2:**
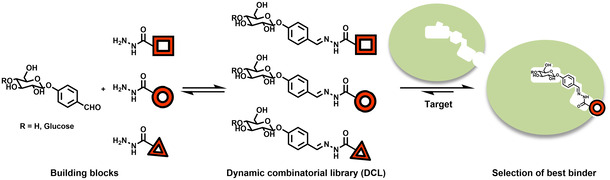
Schematic illustration of target‐directed dynamic combinatorial chemistry (tdDCC) using the acylhydrazone linkage, formed by the reversible reaction between a glucose‐ or maltose‐linked arylaldehyde and three representative hydrazides. Binding to the target protein causes a change in equilibrium, which, in turn, leads to the amplification of the hit compounds.

To identify new ligands of a protein, dynamic combinatorial chemistry (DCC) has become an attractive strategy. DCC allows a target protein to alter the equilibrium of a mixture of products, also known as dynamic combinatorial library (DCL). Due to the change in equilibrium in presence of a protein, good binders get amplified and will therefore be selected as hits (Figure 2). The conditions, reactions, protocols, analysis and applications of DCC were reviewed before.[^17^, ^18^, ^19^, ^20^] For example, the group of Lehn et al. identified inhibitors of the plant lectin concanavalin A using a carbohydrate‐based DCL and dynamic deconvolution.^[21]^ The group of Beau probed DCC to discover binders for the glycosidase hen egg‐white lysozyme (HEWL).^[22]^ Ernst and coworkers used DCC to identify sub‐micromolar binders of the bacterial adhesin FimH by adjusting the ratio of building blocks and establishing a protein‐capturing protocol.^[23]^ Furthermore, we demonstrated that DCC can be applied to challenging targets involved in protein−protein interactions by discovering stabilizers of 14‐3‐3(ζ)−synaptopodin complex.^[24]^ In this work, we exploited the power of DCC to identify new inhibitors of the sugar‐modifying enzyme GS using the well‐established acylhydrazone formation as a reversible reaction. Mono‐ or disaccharide motifs as substrate mimics constitute the building blocks of four DCLs.

## Results and Discussion

### Designing a dynamic combinatorial library

**Figure 3:**
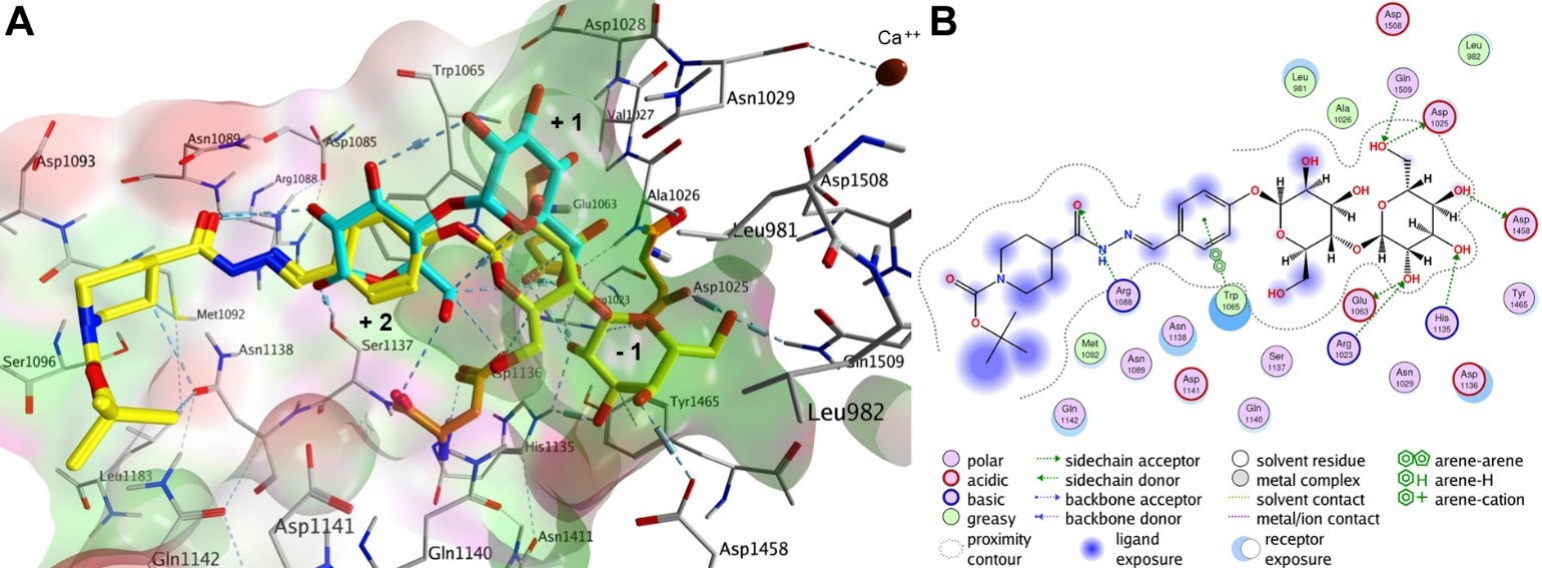
A) Putative binding mode of **A2H2** (yellow) compared to maltose (cyan) in the GTF180‐ΔN active site by using a Molecular Operating Environment (MOE): hydrophobic surface (green), polar surface (magenta), exposed (red). The maltose moiety of **A2H2** occupies the catalytic subsite −1 with a glycosidic bond accessible to the catalytic residues Asp1025, Glu1063 and Asp1136 (orange). B) 2D ligand interactions.

We adopted structure‐based design using the cocrystal structures of *L. reuteri* 180 GTF180‐ΔN complex with two disaccharides, namely sucrose as a donor substrate (PDB ID: 3HZ3) and maltose as an acceptor substrate (PDB ID: 3KLL).^[5]^ The active site of GS is relatively wide and it can be divided into subsites −1 (the catalytic pocket), +1, and +2 (Figure 3A). The donor substrate such as sucrose binds at the catalytic site −1 with its glycosidic linkage in near proximity of the catalytic triad Asp1025, Glu1063, and Asp1136 (highlighted in orange in Figure 3A), and is cleaved by GS. On the other hand, the acceptor substrate, which accepts a glucose molecule such as maltose, binds at subsites +1 and +2 (Figure 3A).

**Scheme 1:**
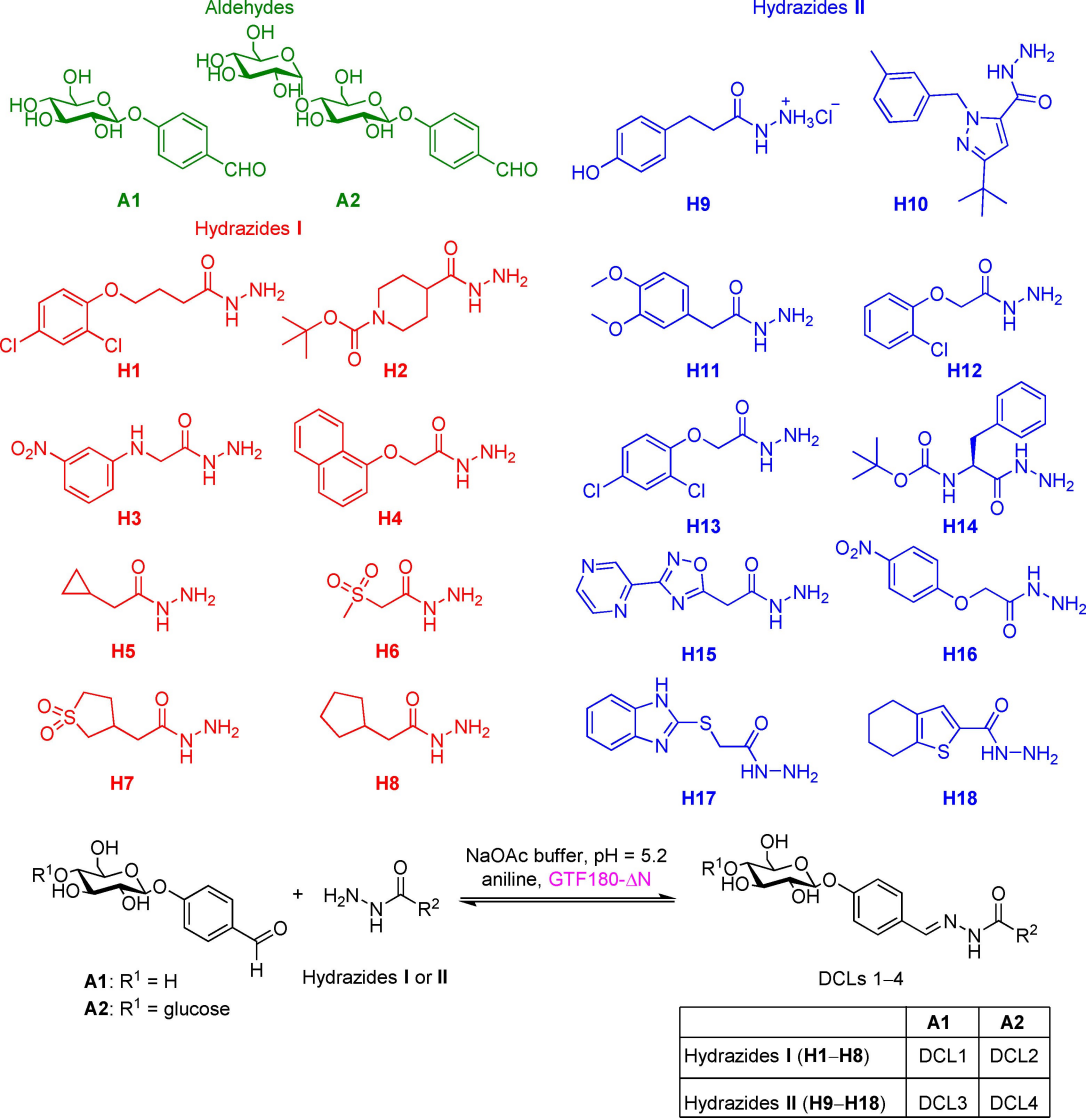
Structures of mono‐ and disaccharide‐based aldehydes (**A1** and **A2**) and hydrazides (**I** and **II**) used in the DCC experiments. Each aldehyde was treated separately with the two hydrazide libraries, resulting in the formation of four dynamic combinatorial libraries (DCLs 1–4).

Inspired by these substrates, we designed the basic scaffold of acylhydrazone by using glucose and maltose linked to benzaldehyde at the *para* position (**A1** and **A2**, respectively, Scheme 1), which would be elongated by a hydrophobic hydrazide moiety for full occupation of the active site. We excluded sucrose (a glucose donor) due to its liability to cleavage by GTFs and opted for glucose instead. On the other hand, *p*‐hydroxybenzaldehyde was chosen for glycosylation as it fits perfectly into subsite +2 permitting π–π interactions with Trp1065 (Figure 3A). Moreover, the *para* position holds the optimum distance between the sugar moiety and the hydrazide, mimicking the α(1→4) glycosidic connection of α‐glucans. Subsequently, we selected 18 chemically different and commercially available hydrazides (**H1**–**H18**) that were divided into two groups (hydrazides **I** and **II**, Scheme 1). Each aldehyde (**A1** or **A2**) was allowed to react separately with both sets of hydrazides (**I** and **II**), resulting in four series of substrate mimetics (DCLs 1–4, Scheme 1).

Docking of all possible 36 acylhydrazones into GTF180‐ΔN active site indicated a favorable binding with high‐energy scores (−7.2 to −10.4 kcal mol^−1^) similar to that of acarbose (Table S1). Compound **A2H2** for instance, is anchored to subsite −1 through the maltose moiety, establishing six H‐bonds with the catalytic residues Asp1025 and Glu1063 as well as Arg1023, His1135, Asp1458, and Gln1509. The aromatic ring of the aldehyde part is located at subsite +2 and involved in a π–π interaction with Trp1065. An additional hydrogen bond is formed between C=O of the acylhydrazone linker as H‐acceptor and NH_2_ of Arg1088 as H‐donor. The piperidine and *tert*‐butyl motifs of the hydrazide in part occupy the distal region of the active site through hydrophobic interactions with Met1092 and Asn1138 and are partially exposed to the solvent (Figure 3). It is worth mentioning that the maltose moiety of the **A2**‐derived acylhydrazones is forced into a different binding site (subsites −1 and +1) and a new binding mode different to that of free maltose. These modifications could affect the binding energy and recognition by the enzyme as an acceptor substrate.

### Synthesis of glucose and maltose‐based building blocks

**Scheme 2:**
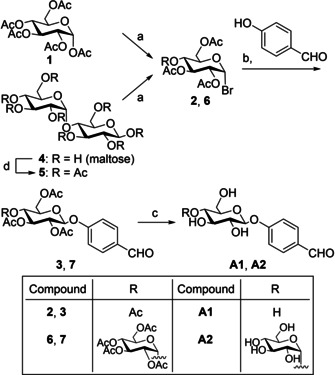
Synthetic route towards aldehydes **A1** and **A2**. a) Ac_2_O, HBr 33 % in AcOH, 0 °C–RT, 15 h, 70 % (**2**) and 52 % (**6**) over two steps; b) Ag_2_O, MeCN, RT, overnight 40 % (**3**) and 62 % (**7**); c) NaOMe, MeOH, Amberlite H^+^ resin, quantitative (**A1**) and 70 % (**A2**); d) Ac_2_O, HClO_4_, AcOH, RT, 1 h.

The aldehydes **A1** and **A2** were synthesized according to the routes shown in Scheme 2. α‐d‐Glucose pentaacetate (**1**) was reacted with a solution of HBr in acetic acid, resulting in 1‐bromo glucose tetraacetate (**2**). The crude product **2** was coupled to *p*‐hydroxybenzaldehyde using silver(I) oxide, yielding compound **3**.^[25]^ Deprotection of the acetate groups by sodium methoxide using the classical Zemplén deacetylation^[26]^ gave aldehyde **A1** in a quantitative yield. The same route was used for the synthesis of **A2**; however, the starting material β‐maltose first had to be acetylated^[27]^ (Scheme 2).

### DCL formation

Each library consisted of one aldehyde (300 μm), one group of hydrazides (300 μm each), aniline (10 mm) and DMSO (10 % *v*/*v*) in sodium acetate buffer (pH 5.2). Aniline enhances the rate at which the acylhydrazone formation reaches equilibrium, as it serves as nucleophilic catalyst to form Schiff bases with the corresponding aldehydes.^[28]^ The use of 10 % DMSO as a cosolvent was feasible thanks to the stability of GTFs at a DMSO concentration of up to 20 %.^[29]^ It assures the solubility of building blocks and products, preventing any undesired shift in equilibrium due to precipitation. A desired shift in the equilibrium, also known as the template effect, was achieved by the addition of the target protein GTF180 (30 μm). The protein was added following a pre‐equilibrated approach, that is, after an equilibrium was reached in the blank library (3 h for these building blocks). A blank reaction (DCL without protein) was prepared in parallel for monitoring the amplification.

### Monitoring the DCLs

**Figure 4:**
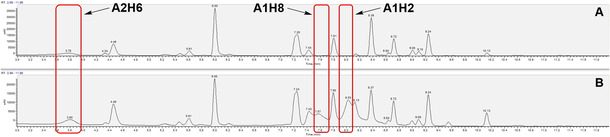
Analysis of dynamic combinatorial library of aldehyde **A2** with hydrazide library **I** (DCL2): UV chromatograms at 290 nm of A) the blank reaction at 6 h and B) the protein‐templated reaction at 6 h.

The DCLs were allowed to stir at room temperature and were regularly monitored by UPLC‐MS on an hourly basis along with the zero‐hour sample. Samples were prepared by taking 100 μL of the corresponding library and raising the pH to >8 by the addition of NaOH (2 m, 8 μL) to freeze the equilibrium, followed by acetonitrile (100 μL) for protein denaturation and liberation of protein‐bound ligands. The mixture was centrifuged at 9720 *g* for 2 min, and the supernatant was subjected to UPLC‐MS analysis. Samples of the blank reaction were treated in the same manner. The formation of the acylhydrazones reached equilibrium within three hours. It was at this time point that we added the GTF180‐▵N and continued the analysis by UPLC‐MS. The distribution of the products in the DCLs of the blank library versus the protein library can be compared by the relative peak areas from the UV chromatograms (Figures 4 and S1–S3, Tables S2–S5). We selected the most amplified ligation product of each library for synthesis and biological evaluation (Table 1).

As aniline could cause false‐positive hits from the reaction with the aldehydes in DCC,^[30]^ we screened the LCMS chromatograms for the possible imine products or hemiaminal intermediates and no corresponding peaks were detected. Other factors could result in false‐positive or false‐negative hits such as concentration and purity of the protein template, buffer composition, and binding interaction (aggregation) between the DCL members.^[31]^ To avoid such pitfalls, we used low concentration of purified GTF180 (0.1 equivalent to the building blocks) and selected the optimum buffer and pH for DCC according to our systematic stability monitoring.^[20]^ Furthermore, we used cheminformatics to exclude potential aggregators from the DCLs through the aggregator advisor.^[32]^

Interestingly, analysis of DCL2, featuring the maltose‐derived aldehyde component, in the presence of GTF after 6 h revealed the formation of two compounds that show molecular weights corresponding to acylhydrazones of the glucose‐based aldehyde **A1** (**A1H2** and **A1H8**). These compounds can result from cleavage of the α‐glycosidic bond between the two sugar units of maltose for the DCL2 members (**A2H2** and **A2H8**; Figure 4 and Table S3). Both compounds possess a nonplanar hydrophobic alicyclic substituent on the hydrazide moiety. This finding indicates that these compounds can indeed bind to the active site, act as a substrate and get cleaved by the enzyme in agreement with our docking study (Figure 3). Accordingly, we also synthesized an example of the substrate molecules (**A2H2**, featuring the hydrazide **H2** that showed favorable amplification in DCL1) and the hydrolysis products (**A1H2** and **A1H8**) in order to investigate their affinity and inhibitory activity toward GTF.

Analysis of the NMR spectra of the prepared acylhydrazones revealed two sets of signals. This can be ascribed to the presence of *cis*/*trans*‐amide CO−NH conformers of the energetically more stable *E* imine C=N configuration as the *Z* isomer is usually disfavored by steric repulsion.[^33^, ^34^, ^35^]

Owing to the reversible nature of the acylhydrazone linkage, we investigated the chemical stability of the compounds under the conditions of SPR and GTF180 activity assays. Encouragingly, all six acylhydrazones showed marked stability at physiological pH 7.4 at room temperature for 24 h as well as at acidic pH 4.7 at 37 °C up to 3 h with less than 5 % degradation as indicated by the UPLC chromatograms (Figures S4–S15). These results are in agreement with previous findings using UV/VIS spectroscopy and ^1^H NMR techniques.[^34^, ^36^]

### Binding studies by surface plasmon resonance

We evaluated the binding affinities of the DCC hits, the substrate compound **A2H2** and the cleavage products by using SPR. The *L. reuteri* GTF180‐ΔN was immobilized covalently to a carboxymethyldextran‐coated sensor chip by amine coupling. In order to ensure that the active site was accessible after immobilization, we used acarbose as a positive control acting through a competitive inhibition mechanism.^[10]^ Binding‐affinity determination for acarbose showed a sub‐millimolar dissociation constant (*K*_D_) of 0.18 mm in line with the reported values for GTFs from other bacterial strains.[^10^, ^37^] Subsequently, we determined the binding affinities of our hit compounds using 6–9 different concentrations in the range of 0.0076–2.0 mm according to their solubility. Except for compound **A2H6**, all compounds showed concentration‐dependent binding responses to GTF180‐ΔN, yet with *K*_D_ values mainly about one order of magnitude higher than acarbose (Table 1 and Figures S17–S23). Generally, the glucose‐bearing acylhydrazones exhibit higher affinity than the maltose derivatives with compound **A1H12** displaying the most promising *K*_D_ value of 0.4 mm. On the other hand, the sensorgrams of the least amplified hit **A2H6** showed low responses up to a concentration of 1 mm and therefore its affinity could not be determined (Figure S22). These unexpectedly weak affinities of the new acylhydrazones may be attributed to the intrinsic weak affinity and very weak inhibitory activity of their sugar moieties especially maltose for GTF.^[10]^ Moreover, the hydrophobic hydrazide moieties of the compounds seem to be unfavorably accommodated in the mainly hydrophilic active site of GTF180. Furthermore, the rather rigid and planar acylhydrazone scaffold possibly hinders a proper fit into the binding cavity.

### GTF180 activity assay

The inhibitory effects of the compounds on GTF180 was assessed by monitoring the hydrolysis of sucrose into glucose using the glucose oxidase/peroxidase (GOPOD) analysis. Acarbose and the compounds at a concentration of 0.5 mm were incubated with the enzyme for 30 min at 37 °C, then sucrose was added, and the hydrolysis products were determined over time to calculate the GTF activity. Consistent with our affinity results, only acarbose showed about 70 % inhibition of GTF180 activity at 0.5 mm, whereas none of the compounds exhibited significant inhibition at the same concentration (Figure S24). Besides the moderate to weak affinity of the compounds, the unobserved residual inhibitory activity can be ascribed to the presence of a high concentration of the substrate sucrose in the assay (20‐fold more than that of the compounds). Such a large excess of the substrate concentration can diminish or abolish the effects of competitive inhibitors.^[38]^ Altogether, these results suggest that the new GTF ligands most probably act via competitive inhibition in agreement with our design rationale.

## Conclusions

We described the first application of DCC to the bacterial glucosyltransferase 180 belonging to the GS family, a potential target for combating dental caries. We designed our compounds to bear a glucose or maltose anchor targeting the active site, resembling natural substrates, as a rational starting point. These molecules were then expected to grow into the pocket by 18 different aromatic or aliphatic tails by DCC. A docking study of the 36 DCC products into the active site of GTF180 supported our rationale, showing high binding energy scores. By separating the complex library into four individual DCLs, we were able to analyze the DCC experiments without overlap among the DCL components. UPLC‐MS analyses of the DCLs resulted in the identification of four most amplified hit compounds, which we synthesized and evaluated for their biophysical and biochemical properties by SPR and in a GTF‐activity assay. Remarkably, we discovered that the maltose‐derived acylhydrazones **A2H2** and **A2H8** with a lipophilic 5/6‐membered alicyclic motif can be cleaved by GTF180, acting as glucose‐donor substrates, in contrast to the parent maltose, which is a known acceptor. This indicates that modification of maltose at the C1‐hydroxy group can alter its recognition by GTF from an acceptor substrate to a donor. Acarbose showed moderate binding affinity for GTF180 (*K*_D_ 0.18 mm) and partial inhibition at 0.5 mm. In comparison to acarbose, the hit compounds showed only moderate to low affinities to GTF180. Results of the activity assay are in line with the SPR measurements, showing no pronounced inhibition at 0.5 mm, most probably due to the weak affinity of the sugar units, hydrophobicity of the hydrazide tails, and overall rigidity of the scaffold due to the acylhydrazone linker. Nevertheless, our endeavor target the sugar‐binding site of GTF180 using DCC resulted in the identification of moderate to weak binders that are indeed capable of binding to the active site as indicated by the cleavage of the maltose derivatives **A2H2** and **A2H8**. This work demonstrates the utility of DCC for notoriously challenging targets such as sugar‐converting enzymes with inherently weak ligand interactions and millimolar affinity.^[22]^ Besides saving time and resources, DCC can be particularly advantageous in absence of structural information or a known ligand to afford novel binders. We gave insight into some challenges encountered by using a carbohydrate‐based scaffold for inhibiting GTFs. Optimization of the physicochemical parameters such as topological polar surface area, flexibility, and water solubility would be required in order to improve the affinity and inhibitory activity of this class. Alternatively, exploration of non‐carbohydrate chemical scaffolds should be envisaged.

## Experimental Section

**Materials and methods**: Chemicals were purchased from commercial suppliers and used without pretreatment. Solvents used for the experiments were reagent‐grade and dried, if necessary, according to standard procedures. The reactions were performed under nitrogen atmosphere, unless otherwise stated. The yields were calculated for the analytically pure compounds and were not optimized. The purifications were performed using column chromatography with Macherey‐Nagel Silica 60 M 0.04–0.063 mm. Preparative HPLC (Ultimate 3000 UHPLC+ focused, Thermo Scientific) purification was performed on a reversed‐phase column (C_18_ column, 5 μm, Macherey‐Nagel, Germany). The solvents used for the chromatography were water (0.1 % formic acid) and MeCN (0.1 % formic acid), or EtOAc and CH_2_Cl_2_. NMR spectra were measured on a Bruker Fourier 500 or Varian AMX400 spectrometers at (500 or 400 MHz for ^1^H) and (126 or 101 MHz for ^13^C), respectively. The chemical shifts are reported in parts per million relative to the corresponding solvent peak. The coupling constants of the splitting patterns are reported in Hertz (Hz).

**UPLC‐MS analysis of DCC**: UPLC‐MS was carried out on a ThermoScientific Dionex Ultimate 3000 UHPLC System coupled to a ThermoScientific Q Exactive Focus with an electrospray ion source. An Acquity Waters Column (BEH, C8 1.7 μm, 2.1×150 mm, Waters, Germany) equipped with a VanGuard Pre‐Column (BEH C8, 5×2.1 mm, 1.7 μm, Waters, Germany) was used for separation. At a flow rate of 0.250 mL/min, the gradient of H_2_O (0.1 % FA) and MeCN (0.1 % FA) was held at 5 % MeCN for 1 min and then increased to 95 % over 16 min. It was held there for 1.5 min before the gradient was decreased to 5 % over 0.1 min where it was held for 1.9 min. The mass spectrum was measured in positive mode in a range from 100–700 *m*/*z*.

**HRMS analysis**: High‐resolution mass spectra were recorded with a ThermoScientific system where a Dionex Ultimate 3000 RSLC was coupled to a Q Exactive Focus mass spectrometer with an electrospray ion source. An Acquity UPLC® BEH C8, 150×2.1 mm, 1.7 μm column equipped with a VanGuard Pre‐Column BEH C8, 5×2.1 mm, 1.7 μm (Waters, Germany) was used for separation. At a flow rate of 250 μL/min, the gradient of H_2_O (0.1 % FA) and MeCN (0.1 % FA) was held at 10 % B for 1 min and then increased to 95 % B over 4 min. It was held there for 1.2 min before the gradient was decreased to 10 % B over 0.3 min where it was held for 1 min. The mass spectrum was measured in positive mode in a range from 120–1000 *m*/*z*. UV spectrum was recorded at 254 nm.

**General procedure for DCC experiments**: The reaction mixture composition for each DCC library was obtained by adding the hydrazides (each 3 μL, stock solutions 100 mm in DMSO) and the aldehyde (3 μL, stock solutions 100 mm in DMSO) to a sodium acetate buffer (590.5 μL, 0.1 m, pH 5.2). Aniline (5.55 μL, stock solution 1.8 m) was added as well as DMSO, to reach a final concentration of DMSO in the DCL of 10 %. Protein (309.5 μL, stock solution 96.93 μm) was added accordingly after 3 h of equilibration. Instead of protein, sodium acetate buffer (309.5 μL) was added to another sample to serve as a blank reaction. Final concentrations in the DCLs were: aniline (10 mm), aldehyde (300 μm), hydrazides (300 μm each), protein (30 μm) and DMSO (10 %). The DCLs were left shaking at room temperature and were concurrently monitored at regular intervals via UPLC‐MS. After 6–7 h of shaking with protein, the mixture was analyzed by UPLC‐MS. For monitoring, 100 μL of the corresponding library was mixed with 8 μL of NaOH (2 m) to raise pH >8, followed by adding 100 μL acetonitrile. The mixture was centrifuged at 9720 *g* for 2 min, and the supernatant was analyzed via UPLC‐MS.

**Chemistry**: Compounds **1** and **4** were purchased. Compounds **2**,^[39]^ **3**,[^40^, ^41^] **5**,^[27]^ **6**,[^42^, ^43^] and **7** ^[40]^ were prepared according to reported procedures.

**4‐(β‐d‐Glucopyranosyloxy)benzaldehyde (A1)**: Compound **A1** was synthesized by a slight modification of the reported method.^[44]^ *p*‐(Tetraacetyl‐β‐d‐glucopyranosyl)benzaldehyde **3**[^40^, ^41^] (225 mg, 0.5 mmol) was dissolved in dry MeOH (3 mL) in a flame‐dried flask under nitrogen. Next, a methanolic NaOMe solution (1.5 m, 0.5 mL) was added dropwise, and the reaction mixture was stirred at room temperature for 4 h. The reaction was neutralized with DOWEX 50WX8 hydrogen‐form ion exchange resin, filtered and passed through a pad of charcoal to remove any colored impurities and then evaporated to dryness in vacuo to obtain pure compound **A1** (137 mg, quantitative). NMR spectra show that **A1** is present as a hydrate form in the NMR solvent. ^1^H NMR (400 MHz, CD_3_OD): *δ*=7.34 (d, *J*=8.6 Hz, 2H), 7.09 (d, *J*=8.7 Hz, 2H), 5.33 (s, 1H), 4.94–4.90 (m, 1H), 3.89 (dd, *J*=12.0, 2.1 Hz, 1H), 3.69 (dd, *J*=12.0, 5.4 Hz, 1H), 3.50–3.29 (m, 4H); ^13^C NMR (101 MHz, CD_3_OD): *δ*=159.2, 133.5, 129.0 (2 C), 117.3 (2 C), 104.2, 102.2, 78.2, 78.0, 74.9, 71.4, 62.5; HRMS (ESI) calcd for C_13_H_15_O_7_ [*M*−H]^–^: 283.0823, found: 283.0822.

**4‐(4‐*O*‐α‐d‐Glucopyranosyl‐β‐d–glucopyranosyloxy)benzaldehyde (A2)**: *p*‐(Heptaacetyl‐β‐d‐maltosyl)benzaldehyde **7** ^[40]^ (340.7 mg, 0.46 mmol) was dissolved in 5 mL dry MeOH in a flame‐dried flask under nitrogen. Next, a methanolic NaOMe solution (1.5 m, 1 mL) was added dropwise, and the reaction was stirred for 4 h at room temperature. The reaction was neutralized with DOWEX 50WX8 hydrogen‐form ion exchange resin, filtered and passed through a pad of charcoal to remove any colored impurities and then evaporated to dryness in vacuo to obtain pure compound **A2** (142 mg, 70 %). ^1^H NMR (400 MHz, CD_3_OD): *δ*=9.84 (s, 1H), 7.87 (d, *J*=8.7 Hz, 2H), 7.34 (d, *J*=8.7 Hz, 2H), 7.23 (d, *J*=8.7 Hz, 2H), 7.08 (d, *J*=8.7 Hz, 2H), 5.32 (s, 1H), 5.21 (t, *J*=3.4 Hz, 2H), 5.09 (d, *J*=7.8 Hz, 1H), 4.96 (d, *J*=7.8 Hz, 1H), 3.96–3.43 (m, 22H), 3.32–3.25 (m, 2H); ^13^C NMR (101 MHz, CD_3_OD): *δ*=193.1, 163.8 (2 C), 159.0 (2 C), 133.4, 132.9, 132.4, 128.9, 117.8 (2 C), 117.2 (2 C), 104.3, 102.7 (2 C), 101.9, 101.2, 80.7, 80.5, 77.6, 77.5, 76.8, 76.6, 75.0 (2 C), 74.7 (2 C), 74.4, 74.3, 74.0 (2 C), 71.4 (2 C), 62.6 (2 C), 61.9 (2 C); HRMS (ESI) calcd for C_19_H_25_O_12_ [*M*−H]^−^: 445.1346, found: 445.1353.

**General procedure for acylhydrazone formation (GP1)**:^[36]^ To the hydrazide (1 equiv) dissolved in MeOH, the corresponding aldehyde (1.2 equiv) was added. The reaction mixture was stirred at room temperature or refluxed until completion. After cooling to room temperature, the reaction mixture was concentrated in vacuo. Purification of acetylated products was performed by column chromatography and deprotected sugars were purified by preparative HPLC, affording the corresponding acylhydrazone in 60 % to quantitative yields.

**General procedure for the deprotection of the acetyl groups (GP2)**:^[26]^ The classical Zemplén deacetylation method of the *O*‐acetyl protecting groups with sodium methoxide in MeOH at room temperature was used. The *O*‐acetyl protected sugar was dissolved in MeOH (0.01 m), and a catalytic amount of sodium methoxide (0.15 equiv) was added. The reaction mixture was stirred at room temperature until complete deprotection was achieved.

***tert*** **‐Butyl 4‐({(2*E*)‐2‐[4‐(β‐d‐glucopyranosyloxy)benzylidene]hydrazino}carbonyl)piperidine‐1‐carboxylate (A1H2)**: The acylhydrazone was synthesized according to GP1 by using 1‐Boc‐isonipecotic acid hydrazide (26 mg, 0.1 mmol) in MeOH (1.0 mL) and *p*‐(β‐d‐glucopyranosyloxy)benzaldehyde **A1** (14.5 mg, 0.05 mmol). After purification, the acylhydrazone was obtained as a white solid (12 mg, 46 %). ^1^H NMR (500 MHz, CD_3_OD): *δ*=8.05 (s, 1H*trans*), 7.88 (s, 1H*cis*), 7.70 (d, *J*=8.6 Hz, 2H*trans*), 7.60 (d, *J*=8.6 Hz, 2H*cis*), 7.15–7.06 (m, 2H*trans*, 2H*cis*), 4.98–4.92 (m, 1H*trans*, 1H*cis*), 4.12 (d, *J*=13.3 Hz, 2H*trans*, 2H*cis*), 3.92–3.84 (m, 1H*trans*, 1H*cis*), 3.69 (dd, *J*=12.1, 5.6 Hz, 1H*trans*, 1H*cis*), 3.51–3.34 (m, 6H*trans*, 7H*cis*), 2.45 (tt, *J*=11.4, 3.6 Hz, 1H*trans*), 1.89–1.52 (m, 4H*trans*, 4H*cis*), 1.45 (s, 9H*trans*, 9H*cis*); ^13^C NMR (126 MHz, CD_3_OD): *δ*=178.6, 173.9, 161.0, 160.6, 156.5, 156.4, 149.3, 145.5, 130.2 (2 C), 129.8 (2 C), 129.5 (2 C), 117.9 (2 C), 117.8 (2 C), 101.9 (2 C), 81.2, 81.1, 78.2 (2 C), 78.0, 77.9, 74.8 (2 C), 71.3 (2 C), 62.5 (2 C), 49.8 (2 C), 42.7 (2 C), 39.5 (2 C), 29.5 (2 C), 28.9 (2 C), 28.7 (6 C); HRMS (ESI) calcd for C_24_H_36_N_3_O_9_ [*M*+H]^+^: 510.2452, found: 510.2432.

**2‐Cyclopentyl‐*N’*‐[(1*E*)‐4‐(2,3,4,6‐tetra‐*O*‐acetyl‐β‐d‐glucopyranosyloxy)benzylidene]acetohydrazide (acetylated A1H8)**: The acylhydrazone was synthesized according to GP1 by using 2‐chlorophenoxyacetic acid hydrazide (18.9 mg, 0.13 mmol) in MeOH (1.8 mL) and *p*‐(tetraacetyl‐β‐d‐glucopyranosyl)benzaldehyde **3** (50.0 mg, 0.11 mmol). After purification, the acylhydrazone was obtained as a white solid (59 mg, 93 %). ^1^H NMR (500 MHz, CD_3_OD): *δ*=8.05 (s, 1H*trans*), 7.89 (s, 1H*cis*), 7.75 (d, *J*=8.8 Hz, 2H*trans*), 7.63 (d, *J*=8.8 Hz, 2H*cis*), 7.07 (d, *J*=8.8 Hz, 2H*trans*, 2H*cis*), 5.46–5.35 (m, 2H*trans*, 2H*cis*), 5.22–5.07 (m, 2H*trans*, 2H*cis*), 4.39–4.24 (m, 1H*trans*, 1H*cis*), 4.21–4.01 (m, 2H*trans*, 2H*cis*), 2.74 (d, *J*=7.4 Hz, 2H*cis*), 2.40–2.12 (m, 3H*trans*, 1H*cis*), 2.10–1.93 (m, 12H*trans*, 12H*cis*), 1.91–1.78 (m, 2H*trans*, 2H*cis*), 1.77–1.53 (m, 4H*trans*, 4H*cis*), 1.38–1.16 (m, 2H*trans*, 2H*cis*); ^13^C NMR (126 MHz, CD_3_OD): *δ*=177.6, 172.4 (2 C), 172.3 (2 C), 171.6, 171.3 (2 C), 171.1 (2 C), 160.2, 159.9, 148.6, 144.8, 130.4 (2 C), 130.3 (2 C), 129.5 (2 C), 117.9 (2 C), 117.8 (2 C), 99.2, 99.1, 74.1 (2 C), 73.1 (2 C), 72.7 (2 C), 69.7 (2 C), 63.1, 63.0, 41.6, 39.4, 38.6, 38.0, 33.5 (2 C), 33.4 (2 C), 25.9 (4 C), 20.6 (4 C), 20.5 (4 C); HRMS (ESI) calcd for C_28_H_37_N_2_O_11_ [*M*+H]^+^: 577.2397, found: 577.2360.

**2‐Cyclopentyl‐*N’*‐[(1*E*)‐4‐(β‐d‐glucopyranosyloxy)benzylidene]acetohydrazide (A1H8)**: The acylhydrazone was synthesized according to GP2 by using acetylated **A1H8** (23.0 mg, 0.04 mmol) in MeOH (4 mL) and sodium methoxide (0.32 mg, 0.006 mmol). After purification, the acylhydrazone was obtained as a white solid (13 mg, 82 %). ^1^H NMR (500 MHz, CD_3_OD): *δ*=8.05 (s, 1H*trans*), 7.88 (s, 1H*cis*), 7.73 (d, *J*=8.8 Hz, 2H*trans*), 7.61 (d, *J*=8.8 Hz, 2H*cis*), 7.23–7.02 (m, 2H*trans*, 2H*cis*), 4.98–4.95 (m, 1H*trans*, 1H*cis*), 3.93–3.87 (m, 1H*trans*, 1H*cis*), 3.74–3.66 (m, 1H*trans*, 1H*cis*), 3.52–3.36 (m, 4H*trans*, 4H*cis*), 2.74 (d, *J*=7.5 Hz, 2H*cis*), 2.43–2.23 (m, 3H*trans*, 1H*cis*), 1.92–1.77 (m, 2H*trans*, 2H*cis*), 1.75–1.52 (m, 4H*trans*, 4H*cis*), 1.41–1.09 (m, 2H*trans*, 2H*cis*); ^13^C NMR (126 MHz, CD_3_OD): *δ*=177.5, 172.3, 160.9, 160.6, 149.0, 145.2, 130.2 (2 C), 129.9 (2 C), 129.6, 129.4, 117.9 (2 C), 117.8 (2 C), 101.9 (2 C), 78.2 (2 C), 78.0, 77.9, 74.9 (2 C), 71.3 (2 C), 62.5 (2 C), 41.6, 39.4, 38.5, 38.0, 33.5 (2 C), 33.4 (2 C), 25.9 (4 C); HRMS (ESI) calcd for C_20_H_29_N_2_O_7_ [*M*+H]^+^: 409.1975, found: 409.1964.

**2‐(2‐Chlorophenoxy)‐*N’*‐[(1*E*)‐4‐(2,3,4,6‐tetra‐*O*‐acetyl‐β‐d‐gluco‐pyranosyloxy)benzylidene]acetohydrazide (acetylated A1H12)**: The acylhydrazone was synthesized according to GP1 by using 2‐chlorophenoxyacetic acid hydrazide (26.6 mg, 0.13 mmol) in MeOH (1.8 mL) and *p*‐(tetraacetyl‐β‐d‐glucopyranosyl)benzaldehyde **3** (50.0 mg, 0.11 mmol). After purification, the acylhydrazone was obtained as a white solid (43 mg, 61 %). ^1^H NMR (500 MHz, CD_3_OD): *δ*=8.19 (s, 1H*trans*), 7.93 (s, 1H*cis*), 7.77 (d, *J*=8.8 Hz, 2H*trans*), 7.66 (d, *J*=8.8 Hz, 2H*cis*), 7.43–7.36 (m, 1H*trans*, 1H*cis*), 7.33–7.19 (m, 1H*trans*, 1H*cis*), 7.14–6.90 (m, 4H*trans*, 4H*cis*), 5.44–5.34 (m, 2H*trans*, 2H*cis*), 5.25 (s, 2H*cis*), 5.21–5.15 (m, 1H*trans*, 1H*cis*), 5.15–5.09 (m, 1H*trans*, 1H*cis*), 4.76 (s, 2H*trans*), 4.35–4.27 (m, 1H*trans*, 1H*cis*), 4.21–4.06 (m, 2H*trans*, 2H*cis*), 2.19–1.76 (m, 12H*trans*, 12H*cis*); ^13^C NMR (126 MHz, CD_3_OD): *δ*=172.3 (2 C), 171.6 (2 C), 171.4, 171.3 (2 C), 171.1 (2 C), 167.1, 160.1, 159.8, 155.5, 155.0, 150.6 (2 C), 146.1 (2 C), 131.5, 131.4, 130.6, 130.3, 130.1, 129.8, 129.3, 128.9, 124.4, 124.1, 124.0, 123.1, 117.9, 117.8, 116.1, 115.3, 99.1 (2 C), 74.1 (2 C), 73.1 (2 C), 72.6 (2 C), 69.7, 69.6, 69.2 (2 C), 67.3 (2 C), 63.1, 63.0, 20.6 (4 C), 20.5 (4 C); HRMS (ESI) calcd for C_29_H_32_ClN_2_O_12_ [*M*+H]^+^: 635.1644, found: 635.1638.

**2‐(2‐Chlorophenoxy)‐*N’*‐[(1*E*)‐4‐(β‐d‐glucopyranosyloxy)benzyl‐idene]acetohydrazide (A1H12)**: The acylhydrazone was synthesized according to GP2 by using acetylated **A1H12** (26.1 mg, 0.04 mmol) in MeOH (4 mL) and sodium methoxide (0.33 mg, 0.006 mmol). After purification, the acylhydrazone was obtained as a white solid in quantitative yield (20 mg). ^1^H NMR (500 MHz, DMSO‐d_6_): *δ*=11.54 (br s, NH, 1H*trans*, 1H*cis*), 8.22 (s, 1H*cis*), 7.96 (s, 1H*trans*), 7.70–7.60 (m, 2H*trans*, 2H*cis*), 7.49–7.37 (m, 1H*trans*, 1H*cis*), 7.34–7.20 (m, 1H*trans*, 1H*cis*), 7.12–6.89 (m, 4H*trans*, 4H*cis*), 5.37 (br s, OH, 1H*trans*, 1H*cis*), 5.25 (s, 2H*trans*), 5.16 (br s, OH, 1H*trans*, 1H*cis*), 5.08 (br s, OH, 1H*trans*, 1H*cis*), 4.96–4.88 (m, 1H*trans*, 1H*cis*), 4.74 (s, 2H*cis*), 4.58 (br s, OH, 1H*trans*, 1H*cis*), 3.69 (d, *J*=11.3 Hz, 1H*trans*, 1H*cis*), 3.53–3.41 (m, 1H*trans*, 1H*cis*), 3.39–3.11 (m, 4H*trans*, 4H*cis*); ^13^C NMR (126 MHz, DMSO‐d_6_): *δ*=168.4, 158.7, 153.7, 153.5, 143.6 (2 C), 130.1 (2 C), 130.0 (2 C), 128.6, 128.4, 128.3, 128.1, 127.8, 127.7, 122.1, 121.5, 121.4, 121.1, 116.4 (2 C), 114.1 (2 C), 113.8 (2 C), 100.0 (2 C), 77.1 (2 C), 76.6 (2 C), 73.2 (2 C), 69.7 (2 C), 69.6 (2 C), 65.3 (2 C), 60.6 (2 C); HRMS (ESI) calcd for C_21_H_24_ClN_2_O_8_ [*M*+H]^+^: 467.1221, found: 467.1205.

***tert*** **‐Butyl 4‐({(2*E*)‐2‐[4‐(4‐*O*‐α‐d‐glucopyranosyl‐β‐d‐glucopyranosyloxy)benzylidene]hydrazine}carbonyl)piperidine‐1‐carboxylate (A2H2)**: The acylhydrazone was synthesized according to GP1 by using 1‐boc‐isonipecotic acid hydrazide (38 mg, 0.15 mmol) in MeOH (1.0 mL) and *p*‐(β‐d‐maltosyl)benzaldehyde **A2** (22 mg, 0.05 mmol). After purification, the acylhydrazone was obtained as a white solid (9.1 mg, 28 %); ^1^H NMR (500 MHz, CD_3_OD): *δ*=8.07 (s, 1H*trans*), 7.90 (s, 1H*cis*), 7.73 (d, *J*=8.8 Hz, 2H*trans*), 7.63 (d, *J*=8.8 Hz, 2H*cis*), 7.15–7.10 (m, 2H*trans*, 2H*cis*), 5.21 (d, *J*=3.8 Hz, 1H*trans*, 1H*cis*), 5.01–4.99 (m, 1H*trans*, 1H*cis*), 4.18–4.09 (m, 2H*trans*, 2H*cis*), 3.96–3.41 (m, 12H*trans*, 12H*cis*), 3.30–3.24 (m, 2H*trans*, 3H*cis*), 2.47 (tt, *J*=11.5, 3.8 Hz, 1H), 1.90–1.54 (m, 4H*trans*, 4H*cis*), 1.47 (s, 9H*trans*, 9H*cis*); ^13^C NMR (126 MHz, CD_3_OD): *δ*=178.6, 173.9, 160.9, 160.6, 156.5, 156.4, 149.3, 145.5, 130.3 (4 C), 129.6, 129.5, 117.9 (2 C), 117.8 (2 C), 102.9 (2 C), 101.7 (2 C), 81.2 (2 C), 81.1, 80.9 (2 C), 80.8, 77.7 (2 C), 76.8 (2 C), 75.1 (2 C), 74.9 (2 C), 74.4 (2 C), 74.2 (2 C), 71.5 (2 C), 62.8 (2 C), 61.9 (2 C), 61.9 (2 C), 42.7 (2 C), 39.5 (2 C), 29.5 (2 C), 28.7 (3 C), 28.7 (3 C); HRMS (ESI) calcd for C_30_H_46_N_3_O_14_ [*M*+H]^+^: 672.2980, found: 672.2958.

***N’*** **‐[(1*E*)‐4‐(4‐*O*‐α‐d‐Glucopyranosyl‐β‐d‐glucopyranosyloxy)benzylidene]‐2‐(methylsulfonyl)acetohydrazide (A2H6)**: The acylhydrazone was synthesized according to GP1 by using 2‐(methylsulfonyl)acetic acid hydrazide (12.8 mg, 0.08 mmol) in MeOH (0.8 mL) and *p*‐(β‐d‐maltosyl)benzaldehyde **A2** (30.2 mg, 0.07 mmol). After purification, the acylhydrazone was obtained as a white solid (4.1 mg, 10 %). ^1^H NMR (500 MHz, CD_3_OD): *δ*=8.10 (s, 1H*trans*), 7.95 (s, 1H*cis*), 7.75 (d, *J*=8.8 Hz, 2H*trans*), 7.66 (d, *J*=8.8 Hz, 2H*cis*), 7.14 (d, *J*=8.8 Hz, 2H*trans*, 2H*cis*), 5.21 (d, *J*=3.8 Hz, 2H*trans*), 5.02–5.01 (m, 1H*trans*, 1H*cis*), 4.68 (s, 2H*cis*), 4.15 (s, 2H*trans*), 3.96–3.38 (m, 12H*trans*, 12H*cis*), 3.19 (s, 3H*cis*), 2.66 (s, 3H); ^13^C NMR (126 MHz, CD_3_OD): *δ*=165.9, 161.2, 161.1, 160.8, 150.8, 146.8, 130.5 (2 C), 129.8 (2 C), 129.4, 129.1, 117.9 (4 C), 102.9 (2 C), 101.7 (2 C), 80.8 (2 C), 77.7 (2 C), 76.8 (2 C), 75.1 (2 C), 74.8 (2 C), 74.4 (2 C), 74.2 (2 C), 71.5 (2 C), 62.8, 61.9, 60.0, 57.4, 42.6, 42.0, 40.4 (2 C); HRMS (ESI) calcd for C_22_H_33_N_2_O_14_S [*M*+H]^+^: 581.1652, found: 581.1620.

**2‐(2‐Chlorophenoxy)‐*N’*‐{(1*E*)‐4‐[2,3,6‐tri‐*O*‐acetyl‐4‐*O*‐(2,3,4,6‐tetra‐*O*‐acetyl‐α‐d‐glucopyranosyl)‐β‐d‐glucopyranosyloxy]benzylidene}acetohydrazide (Acetylated A2H12)**: The acylhydrazone was synthesized according to GP1 by using 2‐chlorophenoxyacetic acid hydrazide (8.9 mg, 0.04 mmol) in MeOH (0.5 mL) and *p*‐(heptaacetyl‐β‐d‐maltosyl)benzaldehyde **7** (25.0 mg, 0.03 mmol). After purification, the acylhydrazone was obtained as a white solid (30 mg, 48 %). ^1^H NMR (500 MHz, CD_3_OD): *δ*=8.19 (s, 1H*trans*), 7.92 (s, 1H*cis*), 7.77 (d, *J*=8.8 Hz, 2H*trans*), 7.66 (d, *J*=8.8 Hz, 2H*cis*), 7.45–7.19 (m, 3H*trans*, 2H*cis*), 7.15–6.90 (m, 3H*trans*, 4H*cis*), 5.50–5.33 (m, 4H*trans*, 4H*cis*), 5.24 (s, 2H*cis*), 5.11–5.00 (m, 2H*trans*, 2H*cis*), 4.93–4.85 (m, 1H*trans*, 1H*cis*), 4.76 (s, 2H*trans*), 4.62 (s, 2H*cis*), 4.60–4.50 (m, 2H*trans*), 4.36–4.03 (m, 6H*trans*, 6H*cis*), 2.12–1.96 (m, 21H*trans*, 21H*cis*); ^13^C NMR (126 MHz, CD_3_OD): *δ*=172.3 (4 C), 171.9 (2 C), 171.8 (2 C), 171.6 (2 C), 171.3 (2 C), 171.2 (2 C), 167.1, 160.1, 159.7, 155.5, 154.9, 154.8, 150.6, 146.1, 131.5, 131.4 (2 C), 130.6, 130.3, 130.0, 129.8, 129.3, 129.2, 128.9, 124.4, 124.1, 124.0, 123.9, 123.1, 117.9, 117.8, 116.1, 115.7, 115.3, 98.7 (2 C), 97.3 (2 C), 76.4 (2 C), 75.0, 74.9, 73.6 (2 C), 73.3 (2 C), 71.7 (2 C), 70.7, 69.9, 69.7 (2 C), 69.2 (2 C), 68.8, 67.3, 64.3 (2 C), 63.1 (2 C), 21.2 (2 C), 20.8 (4 C), 20.7 (2 C), 20.6 (6 C); HRMS (ESI) calcd for C_41_H_48_ClN_2_O_20_ [*M*+H]^+^: 923.2489, found: 923.2446.

**2‐(2‐Chlorophenoxy)‐*N’*‐[(1*E*)‐4‐(4‐*O*‐α‐d–glucopyranosyl‐β‐d‐glucopyranosyloxy)benzylidene]acetohydrazide (A2H12)**: The acylhydrazone was synthesized according to GP2 by using acetylated **A2H12** (27.0 mg, 0.03 mmol) in MeOH (3 mL) and sodium methoxide (0.36 mg, 0.007 mmol). After purification, the acylhydrazone was obtained as a white solid (17.2 mg, 93 %). ^1^H NMR (500 MHz, CD_3_OD): *δ*=8.19 (s, 1H*trans*), 7.93 (s, 1H*cis*), 7.76 (d, *J*=8.8 Hz, 2H*trans*), 7.65 (d, *J*=8.8 Hz, 2H*cis*), 7.43 (dd, *J*=7.9, 1.6 Hz, 1H*trans*), 7.38 (dd, *J*=7.9, 1.6 Hz, 1H*cis*), 7.33–7.26 (m, 1H*trans*), 7.26–7.20 (m, 1H*cis*), 7.18–7.07 (m, 3H*trans*, 2H*cis*), 7.06–6.90 (m, 1H*trans*, 2H*cis*), 5.26 (s, 2H*cis*), 5.21 (d, *J*=3.7 Hz, 1H*trans*, 1H*cis*), 5.05–4.97 (m, 1H*trans*, 1H*cis*), 4.76 (s, 2H*trans*), 3.97–3.41 (m, 10H*trans*, 10H*cis*), 3.31–3.23 (m, 2H*trans*, 2H*cis*); ^13^C NMR (126 MHz, CD_3_OD): *δ*=171.4, 167.1, 161.1, 160.7, 155.6, 155.0, 151.0 (2 C), 146.4 (2 C), 131.5, 131.3, 130.5 (2 C), 129.7 (2 C), 129.6, 129.3 (2 C), 128.9, 124.4, 124.1, 124.0, 123.1, 117.9, 117.8, 116.1, 115.2, 102.9 (2 C), 101.7 (2 C), 80.9, 80.8, 77.7 (2 C), 76.8 (2 C), 75.1 (2 C), 74.9 (2 C), 74.5 (2 C), 74.2 (2 C), 71.5 (2 C), 69.2 (2 C), 67.3 (2 C), 62.8, 61.9; HRMS (ESI) calcd for C_27_H_34_ClN_2_O_13_ [*M*+H]^+^: 629.1749, found: 629.1744.

**Computational chemistry**: All computational work was performed using Molecular Operating Environment (MOE), version 2019.01 (Chemical Computing Group ULC, Montreal, Canada). The computational procedure was adopted from reported protocols[^45^, ^46^] with a slight modification as follows.

**Preparation of ligands and protein structure for docking**: The 2D structures of 36 acylhydrazone products of DCL1–4 were sketched using ChemDraw professional 17.0 and were pasted to the MOE window. The compounds were subjected to an energy minimization up to a gradient of 0.01 kcal mol^−1^ Å^2^ using the MMFF94x force field, then they were saved as mdb file. In the database viewer window, the acylhydrazone structures were washed via compute | molecule | wash command. Deprotonation of strong acids and protonation of strong bases were performed by choosing the dominant protonation at pH 7 option in the wash panel. The X‐ray crystal structure of the *L. reuteri* 180 GTF180‐ΔN in complex with maltose (PDB ID: 3KLL)^[5]^ was used to perform the molecular docking study. The potential was set up to Amber10:EHT as a force field and R‐field for solvation. Addition of hydrogen atoms, removal of water molecules farther than 4.5 Å from ligand or receptor, correction of library errors, and tethered energy minimization of binding site were performed by using QuickPrep module.

**Ligand−receptor docking**: The binding site was set to dummy atoms, which were calculated by the site‐finder command, and the amino acid residues were chosen where maltose binds in the GTF180‐ΔN active site. Docking placement was triangle matcher with an induced fit refinement option. The first scoring function was alpha HB with 100 poses, followed by a refinement score affinity dG with 10 poses.

**Chemical‐stability determinations**: Stability studies of the compounds in the SPR and GTF activity assays were performed as previously reported^[47]^ with slight modifications. Two sets of samples were prepared according to the assay conditions. For SPR, 5 μL of 2 mm stock solution of the compounds in DMSO was added to 95 μL of HEPES buffer (10 mm HEPES, 150 mm NaCl, 3 mm EDTA, 0.005 % *v*/*v* Tween 20, pH 7.4) and vortexed to attain a final concentration of 100 μM in a total volume (100 μL) containing 5 % DMSO. The solutions of the compounds were stirred in a shaking mixer at 10 rpm at RT. Aliquots of 5 μL were taken from the samples at time 0, 3, and 24 h, mixed with MeOH (45 μL) to have a final concentration 10 μm, and samples were submitted for UPLC‐MS analysis. In the other set for GTF activity assay, 5 μL of 2 mm stock solution of the compounds in DMSO was added to a mixture of 80 μL of sodium acetate buffer (100 mm, 8 mm CaCl_2_, pH 4.7) and DMSO (15 μL) and vortexed to reach a final concentration of 100 μm in a total volume (100 μL) containing 20 % DMSO. The solutions of the compounds were incubated in a water bath at 37 °C. 5 μL Aliquots were taken from the samples at time 0, 0.5, 1, and 3 h, mixed with MeOH (45 μL) and submitted for analysis. The samples were stored at −20 °C, if they were not measured directly.

LCMS analyses were measured by Dionex UltiMate 3000 UHPLC+ focused/Thermo Scientific ISQ EC mass spectrometer system (Thermo Fisher Scientific, Dreieich, Germany). The system consists of Dionex UltiMate 3000 RS pump, RS autosampler, column compartment, diode array detector, and single‐quadrupole mass spectrometer, as well as the standard software Chromeleon 7.2.9 for operation. RP Hypersil GOLD C_18_, 1.9 μm (100 mm×2.1 mm) column (Thermo Scientific, Dreieich, Germany) was used as stationary phase, and a binary solvent system A and B (A=water with 0.1 % FA; B=MeCN with 0.1 % FA) was used as mobile phase. In a gradient run, the percentage of B was increased from an initial concentration of 5 % at 0 min to 100 % at 4.2 min and kept at 100 % for 0.8 min. The injection volume was 5 μL and the flow rate was set to 600 μL/min. For compound **A2H6**, the initial concentration of B was 1 % and the injection volume was 10 μL. The column temperature was 40 °C and UV tracing was acquired at wavelength of 310 nm. MS (HESI) analysis was carried out at a spray voltage of 3000 V (positive), and −2000 V (negative), and an ion transfer tube temperature of 300 °C. Spectra were acquired in positive and negative mode from 100 to 1000 *m*/*z*.

**Binding studies by surface plasmon resonance**: The SPR experiments were performed using a Reichert SR7500DC surface plasmon resonance spectrometer (Reichert Technologies, Depew, NY, USA), and medium density carboxymethyl dextran hydrogel CMD500 M sensor chips (XanTec Bioanalytics, Düsseldorf, Germany). Doubly distilled (dd) water was used as the running buffer for immobilization. HEPES buffer (10 mm HEPES, 150 mm NaCl, 3 mm EDTA, 0.005 % *v*/*v* tween 20, pH 7.4) containing 5 % *v*/*v* DMSO was used as the running buffer for binding study. All running buffers were filtered and degassed prior to use. GTF180 (117 kDa) was immobilized in one of the two flow cells by amine coupling according to reported procedure.[^24^, ^48^] The other flow cell was left blank to serve as a reference. The system was initially primed with borate buffer 100 mm (pH 9.0), then the carboxymethyldextran matrix was activated by a 1 : 1 mixture of *N*‐ethyl‐*N*′‐(3‐dimethylaminopropyl)carbodiimide hydrochloride (EDC) 100 mm and *N*‐hydroxysuccinimide (NHS) 100 mm at a flow rate of 10 μL/min for 7 min. GTF180 5 μm in 10 mm sodium acetate buffer (pH 4.0) was injected at a flow rate of 10 μL/min for 7 min. Unreacted surface was quenched by 1 m ethanolamine hydrochloride (pH 8.5) at a flow rate of 25 μL/min for 3 min. A series of 10 buffer injections was run initially on both reference and active surfaces to equilibrate the system resulting in a stable immobilization level of approximately 2000 μ refractive index unit (μRIU; Figure S16). Binding experiments were performed at 20 °C. Compounds dissolved in DMSO were diluted with the running buffer (final DMSO concentration of 5 % *v*/*v*) and were injected at a flow rate of 30 μL/min. Single‐cycle kinetics were applied for *K*_D_ determination. The association time was set to 60 s, and the dissociation phase was recorded for 120 s. Ethylene glycol 80 % in the running buffer was used for regeneration of the surface. Differences in the bulk refractive index due to DMSO were corrected by a calibration curve (nine concentrations: 3–7 % *v*/*v* DMSO in HEPES buffer). Data processing and analysis were performed by Scrubber software (Version 2.0c, 2008, BioLogic Software). Sensorgrams were calculated by sequential subtractions of the corresponding curves obtained from the reference flow cell and the running buffer (blank). SPR responses are expressed in resonance unit (RU). The *K*_D_ values were calculated by global fitting of the kinetic curves as well as fitting of the steady state binding responses to a 1 : 1 Langmuir interaction model.

**GTF180‐ΔN activity assay**: In an 8‐well PCR strip containing 50 μL sodium acetate buffer (100 mm, pH 4.7, 8 mm CaCl_2_), 20 μL DMSO, 20 μL compound (5 mm in DMSO), and 70 μL Milli‐Q H_2_O, 20 μL GTF180‐▵N (4.5 μm) was added and the mixture was incubated for 30 min at 37 °C. Acarbose (5 mm in DMSO) and DMSO were used as a positive and negative control, respectively. To each well of a 96‐wells PCR plate, 12.5 μL NaOH (0.4 m) was added. The wells of a new 8‐well PCR strip were filled with 200 μL sucrose solution (100 mm) and incubated at 37 °C for 5 min before starting the assay. The assay was started by adding 20 μL of the 100 mm sucrose stock to the wells containing the compound and GTF180‐ΔN mixture to obtain a final volume of 200 μL. Every 30 s, a 25 μL sample was taken and mixed immediately with 12.5 μL NaOH (0.4 m) to stop the enzymatic activity. After the last time point at 3.5 min, 12.5 μL HCl (0.4 m) was added to neutralize the samples. The amount of glucose released from sucrose was measured with a glucose assay kit (glucose oxidase/peroxidase; GOPOD, Megazyme International Ireland Ltd., Ireland). For the GOPOD analysis, 12.5 μL of the neutralized samples was mixed with 187.5 μL GOPOD and incubated at 37 °C for 30 min. Absorbances were read at 510 nm and glucose concentrations were calculated from a trendline of glucose concentrations ranging from 25 to 0.195 mm.

## Conflict of interest

The authors declare no conflict of interest.

## Supporting information

As a service to our authors and readers, this journal provides supporting information supplied by the authors. Such materials are peer reviewed and may be re‐organized for online delivery, but are not copy‐edited or typeset. Technical support issues arising from supporting information (other than missing files) should be addressed to the authors.

Supplementary
