# Supplementary material for: Potential Dental Biofilm Inhibitors: Dynamic Combinatorial Chemistry Affords Sugar‐Based Molecules that Target Bacterial Glucosyltransferase
Source: ChemMedChem. 2020 Jul 9;16(1):113–23. doi: 10.1002/cmdc.202000222 (PMC7818428; doi:10.1002/cmdc.202000222)
Supplement: Supplementary file 1 — Supplementary [file CMDC-16-113-s001.pdf]

ChemMedChem

Supporting Information

Potential Dental Biofilm Inhibitors: Dynamic Combinatorial Chemistry Affords Sugar-Based Molecules that Target Bacterial Glucosyltransferase

Alwin M. Hartman[+], Varsha R. Jumde[+], Walid A. M. Elgaher[+], Evelien M. Te Poele, Lubbert Dijkhuizen, and Anna K. H. Hirsch*

Table of Contents

Molecular docking of the designed acylhydrazones ……………………………………………S3

UPLC-MS analysis of DCLs 1–4 ....S12

Chemical stability under the assays conditions …………………………..……………………..S16

Binding studies by surface plasmon resonance (SPR) S28

GTF180-ΔN activity assay...S31

LC-MS analysis and NMR spectra S32

Table S1. Binding energy scores and 2D ligand–protein interactions of the DCL constituents.

DCL	Compound	2D Ligand–protein interactions	Binding score (kcal/mol)
DCL1	**A1H1**	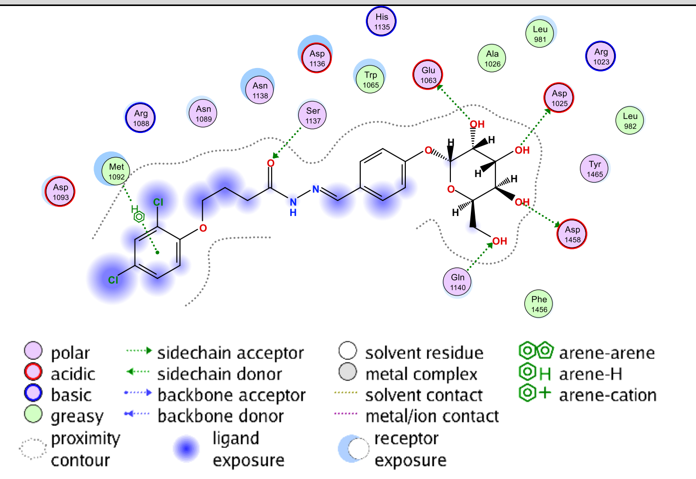	–8.2 to –7.0
	**A1H2**	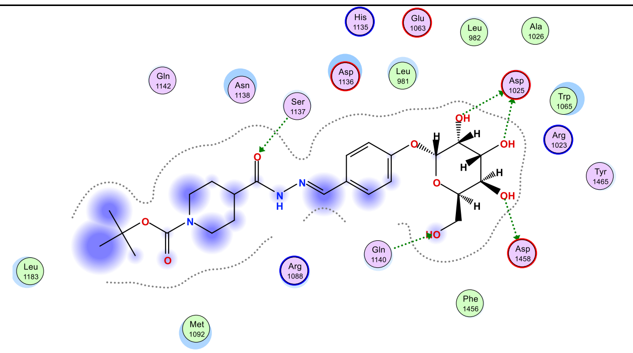	–7.7 to –7.2
	**A1H3**	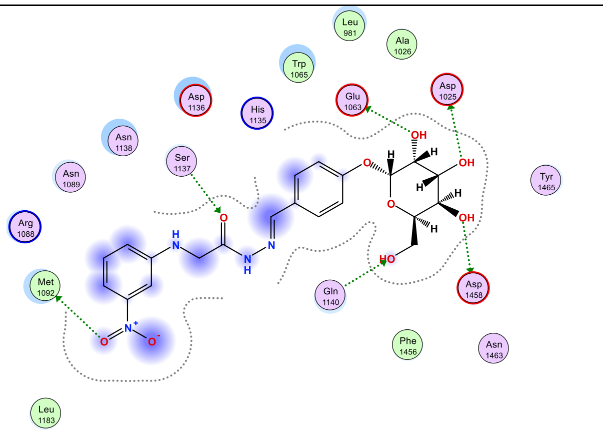	–7.3 to –6.6
	**A1H4**	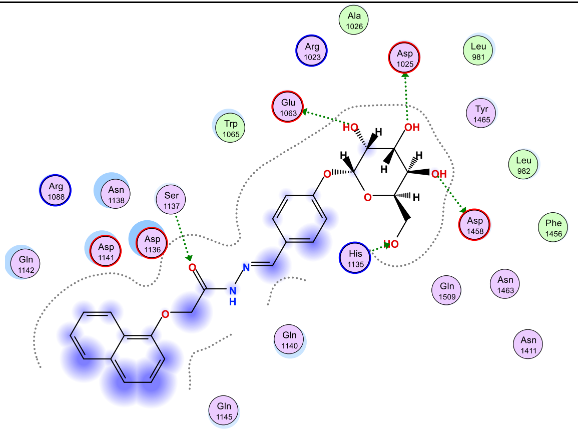	–7.5 to –6.9

DCL1	**A1H5**	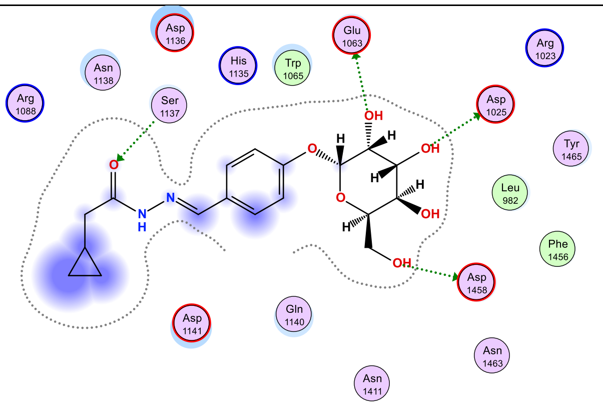	–8.0 to –7.4
	**A1H6**	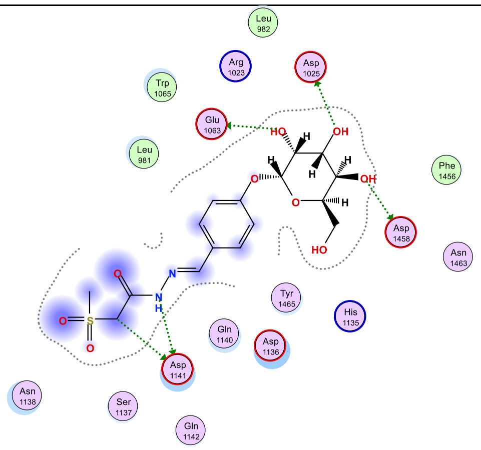	–8.4 to –7.3
	**A1H7**	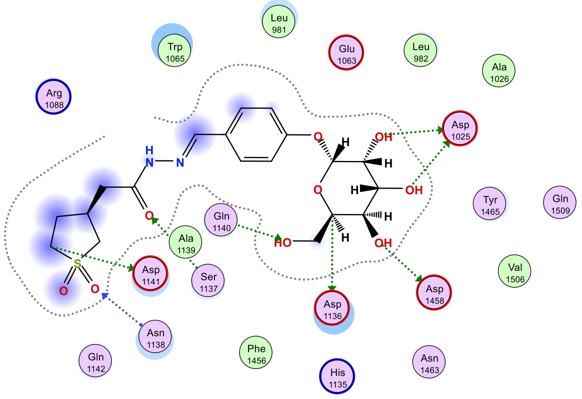	–7.7 to –7.4
	**A1H8**	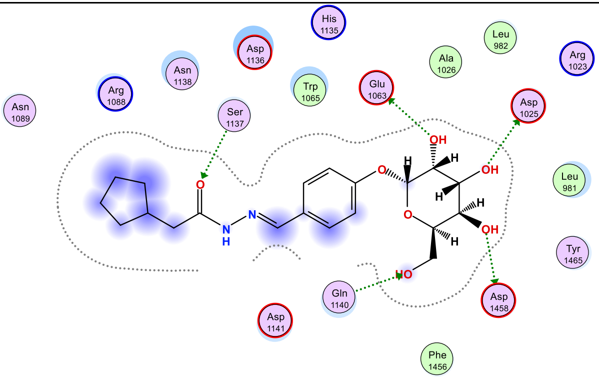	–7.5 to –7.0

DCL2	**A2H1**	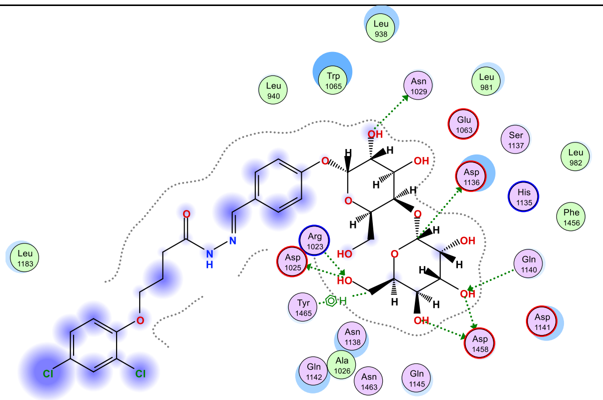	–9.1 to –7.6
	**A2H2**	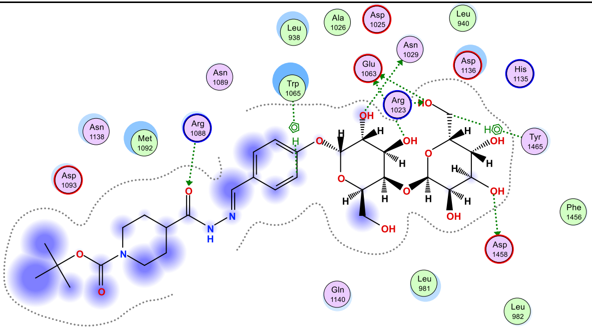	–8.7 to –8.3
	**A2H3**	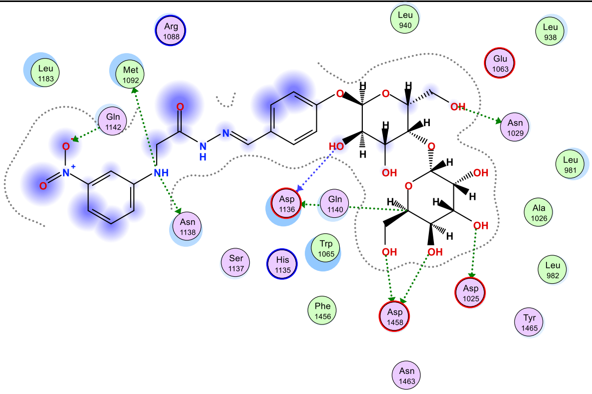	–8.5 to –8.1
	**A2H4**	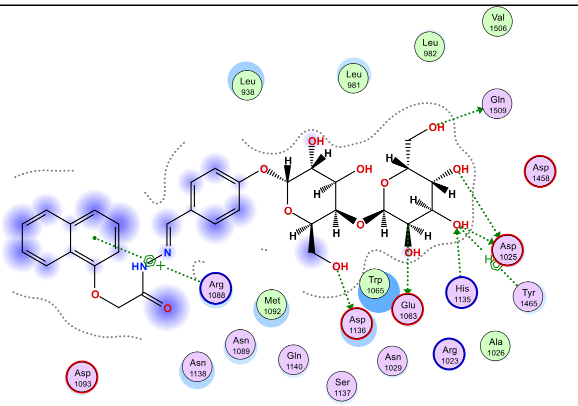	–10.4 to –7.7

DCL2	**A2H5**	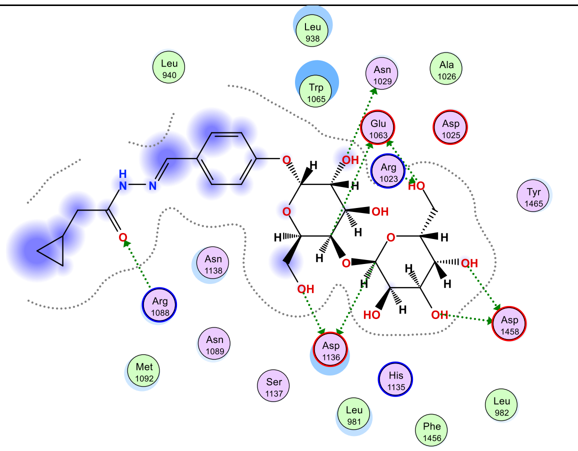	–8.6 to –7.1
	**A2H6**	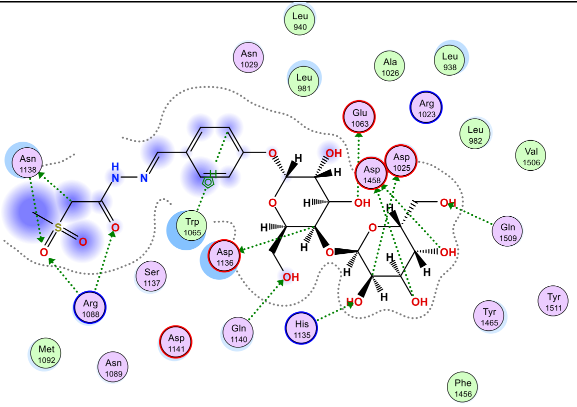	–8.4 to –7.6
	**A2H7**	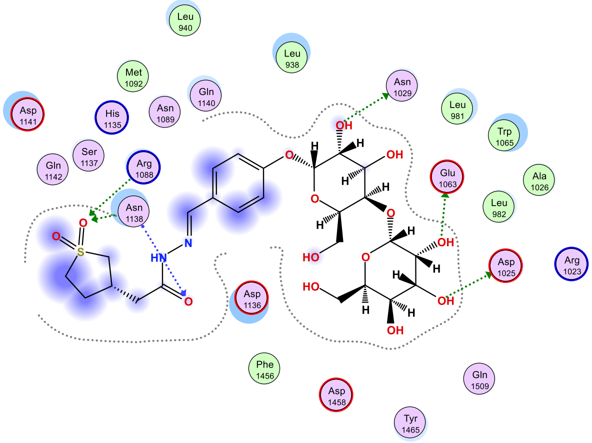	–8.7 to –7.7
	**A2H8**	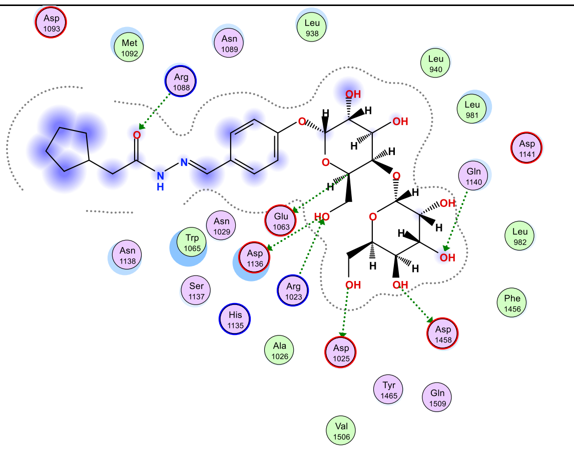	–7.6 to –6.9

DCL3	**A1H9**	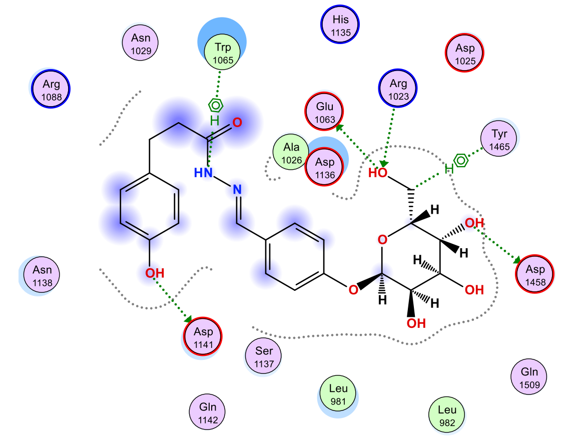	–7.2 to –6.7
	**A1H10**	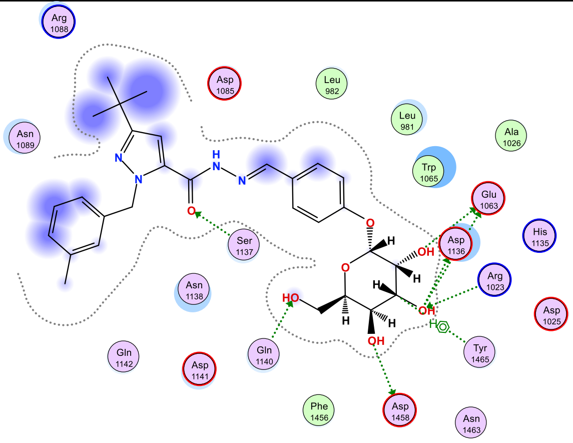	–7.6 to –6.9
	**A1H11**	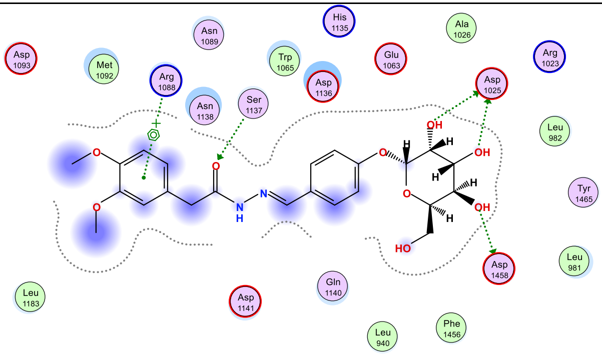	–8.2 to –7.4
	**A1H12**	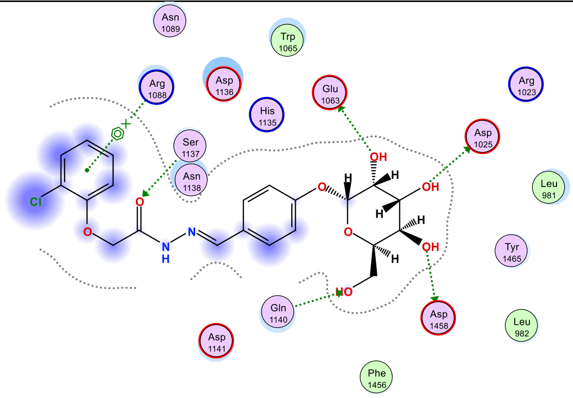	–8.4 to –6.6

DCL3	**A1H13**	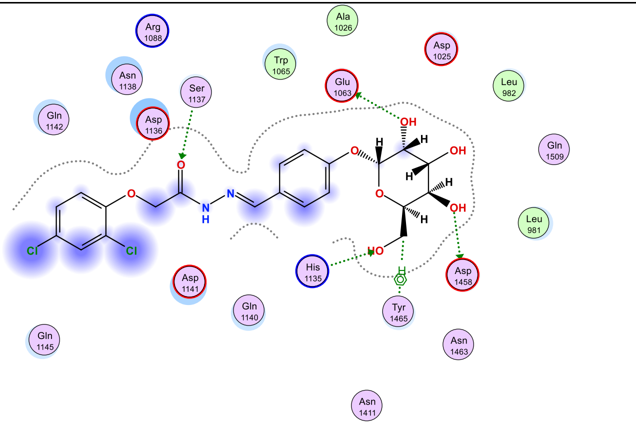	–8.0 to –7.0
	**A1H14**	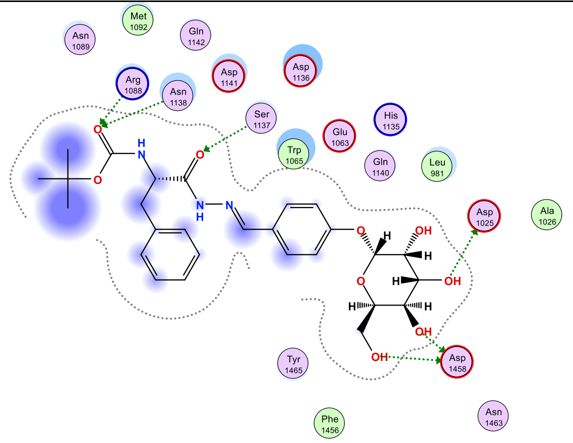	–8.5 to –7.3
	**A1H15**	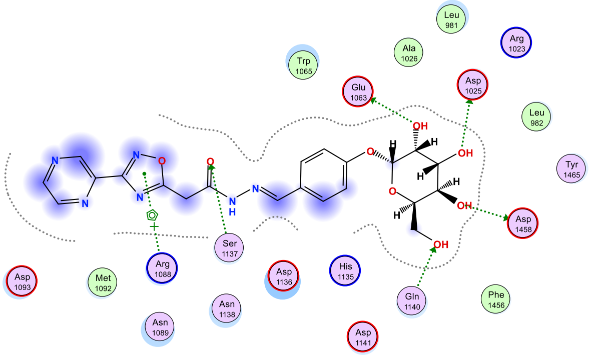	–8.7 to –7.5
	**A1H16**	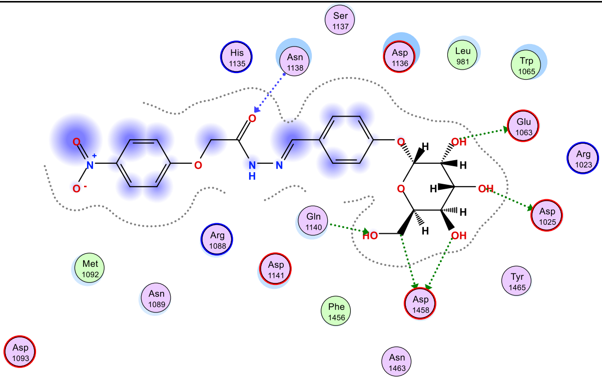	–8.3 to –6.6

<table>
<tr><td rowspan="2">DCL3</td><td>A1H17</td><td>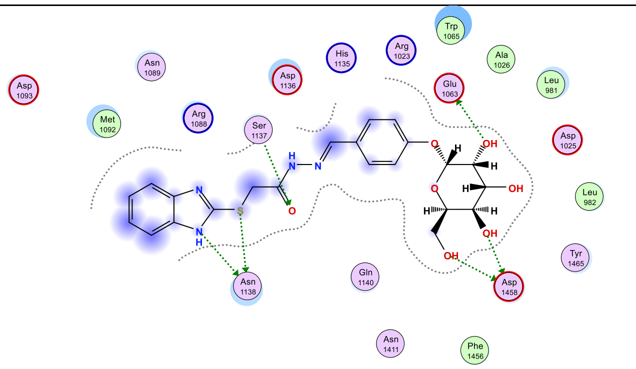
</td><td>–7.7 to –6.6</td></tr>
<tr><td>A1H18</td><td>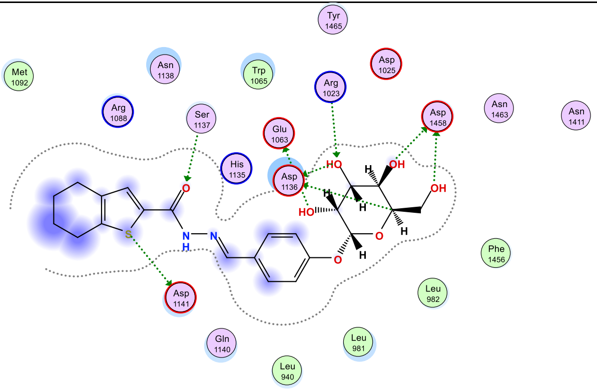
</td><td>–7.5 to –7.1</td></tr>
<tr><td rowspan="2">DCL4</td><td>A2H9</td><td>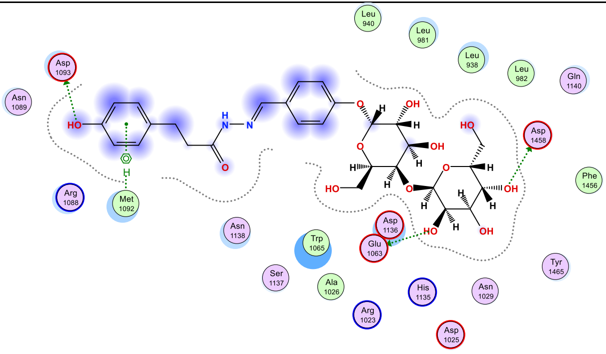
</td><td>–9.0 to –7.9</td></tr>
<tr><td>A2H10</td><td>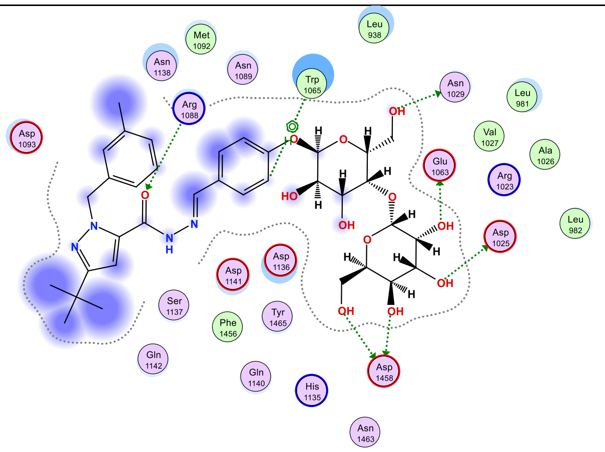
</td><td>–10.0 to –8.5</td></tr>
</table>

DCL4	**A2H11**	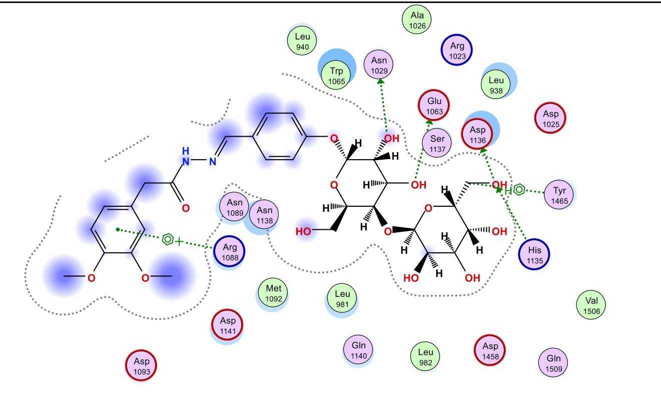	–7.7 to –7.1
	**A2H12**	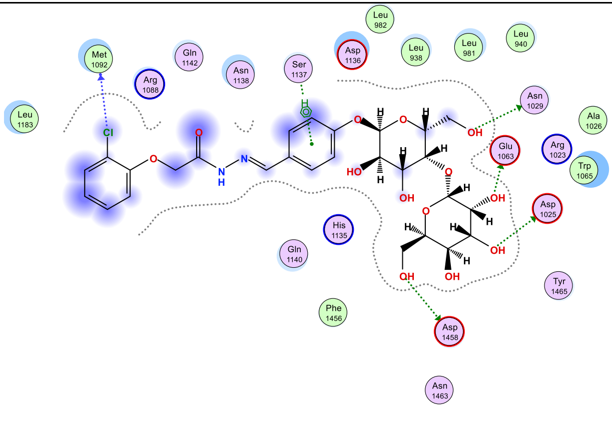	–8.7 to –8.1
	**A2H13**	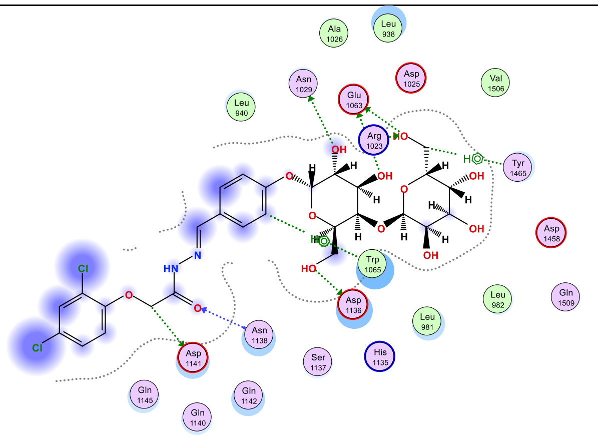	–9.1 to –7.6
	**A2H14**	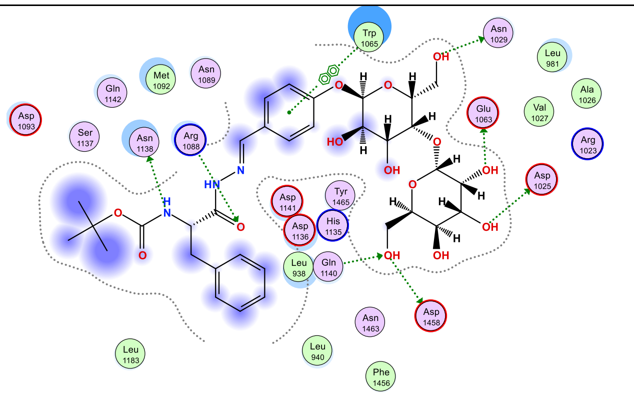	–8.7 to –8.1

DCL4	**A2H15**	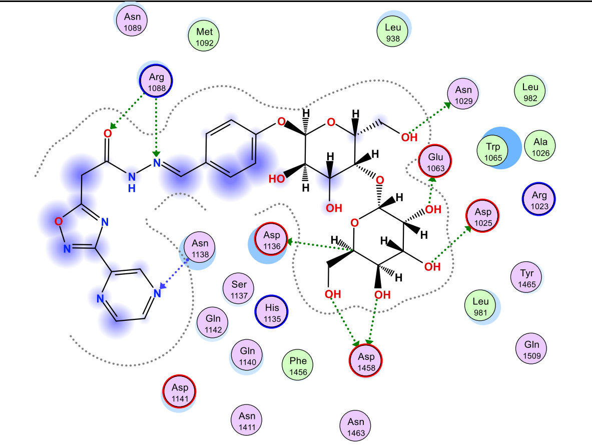	–9.3 to –8.7
	**A2H16**	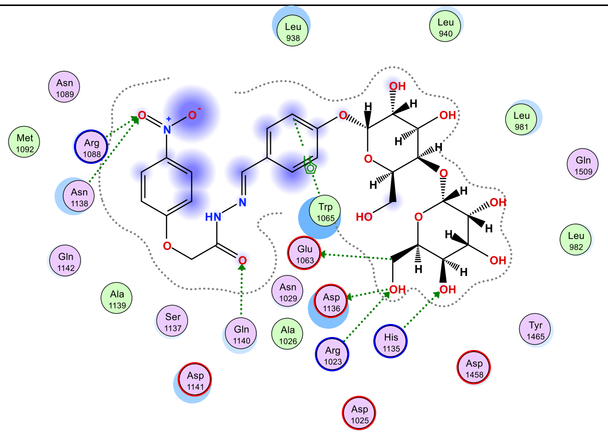	–9.9 to –8.3
	**A2H17**	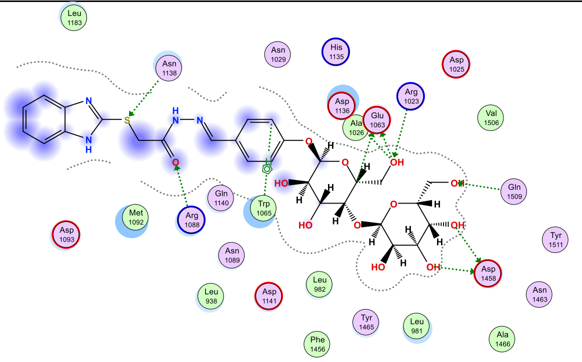	–9.0 to –7.9
	**A2H18**	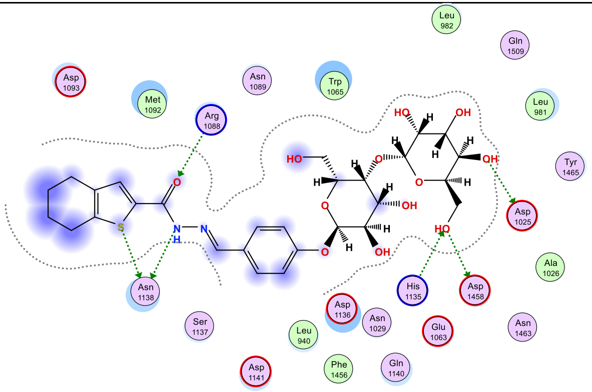	–9.4 to –7.2

Acarbose	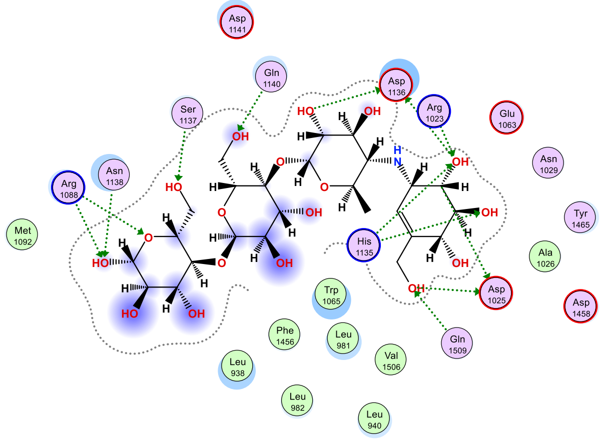	–10.3 to –9.7

UPLC-MS analysis of DCLs 1–4

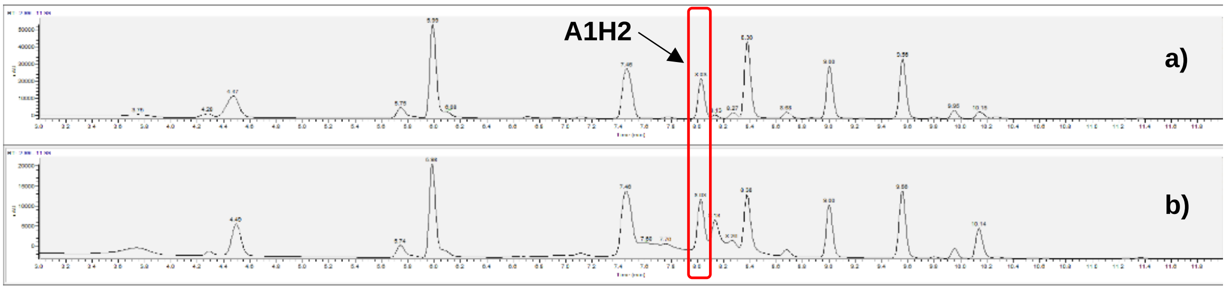

Figure S1. Analysis of dynamic combinatorial library DCL1 of aldehyde **A1** with hydrazides library **I**: a) UV-chromatogram at 290 nm of the blank reaction at 7 h; b) UV-chromatogram at 290 nm of the protein-templated reaction at 7 h.

Table S2. Amplification folds of the formed products in DCL1; analysed via the relative surface areas of peaks in the UV-chromatograms of the protein-templated reaction (P) and blank reaction (B). Data obtained from single experiment.

Acylhydrazone	UV-retention time (min)	Amplification fold (%P/%B)
**A1H1**	9.6	0.9
**A1H2**	8.1	1.8
**A1H3**	7.5	1.1
**A1H4**	9.0	0.8
**A1H5**	5.8	0.8
**A1H6**	3.8	1.2
**A1H7**	4.5	0.9
**A1H8**	7.5	1.1

Table S3. Amplification folds of the formed products in DCL2; analysed via the relative surface areas of peaks in the UV-chromatograms of the protein-templated reaction (P) and blank reaction (B). Data obtained from single experiment.

Acylhydrazone	UV-retention time (min)	Amplification folds (%P/%B)
**A2H1**	9.2	0.8
**A2H2**	7.8	0.9
**A2H3**	7.3	0.8
**A2H4**	8.7	0.8
**A2H5**	5.6	0.7
**A2H6**	3.8	1.5
**A2H7**	4.5	1.1
**A2H8**	7.3	0.8
**A1H2**	8.03	-
**A1H8**	7.57	-

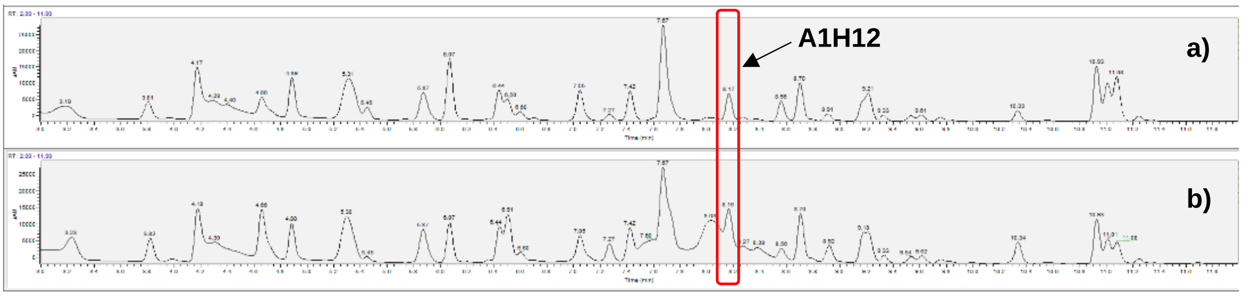

Figure S2. Analysis of dynamic combinatorial library DCL3 of aldehyde **A1** with hydrazide library **II**: a) UV-chromatogram at 290 nm of the blank reaction at 6 h; b) UV-chromatogram at 290 nm of the protein-templated reaction at 6 h. Data obtained from single experiment.

Table S4. Amplification folds of the formed products in DCL3; analysed *via* the relative surface areas of peaks in the UV-chromatograms of the protein-templated reaction (P) and blank reaction (B). Data obtained from single experiment.

Acylhydrazone	UV-retention time (min)	Amplification fold (%P/%B)
**A1H9**	5.9	0.8
**A1H10**	10.9	0.7
**A1H11**	6.5	1.2
**A1H12**	8.2	2.1
**A1H13**	9.2	1.1
**A1H14**	9.2	1.1
**A1H15**	5.4	0.8
**A1H16**	7.4	1.0
**A1H17**	5.4	0.8
**A1H18**	8.7	1.0

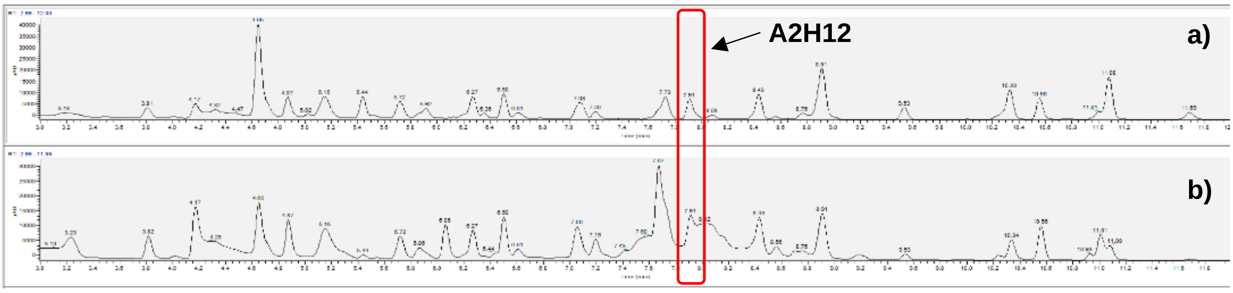

Figure S3. Analysis of dynamic combinatorial library DCL4 of aldehyde **A2** with hydrazide library **II**: a) UV-chromatogram of the blank reaction at 6 h; b) UV-chromatogram of the protein-templated reaction at 6 h. Data obtained from single experiment.

Table S5. Amplification folds of the formed products in DCL4; analysed *via* the relative surface areas of peaks in the UV-chromatograms of the protein-templated reaction (P) and blank reaction (B). Data obtained from single experiment.

Acylhydrazone	UV-retention time (min)	Amplification fold (%P/%B)
**A2H9**	5.7	1.0
**A2H10**	10.6	1.0
**A2H11**	6.2	1.0
**A2H12**	7.9	3.2
**A2H13**	8.9	0.6
**A2H14**	8.9	0.6
**A2H15**	-	-
**A2H16**	7.2	2.2
**A2H17**	5.2	0.1
**A2H18**	8.4	1.2

Chemical stability under the assays conditions

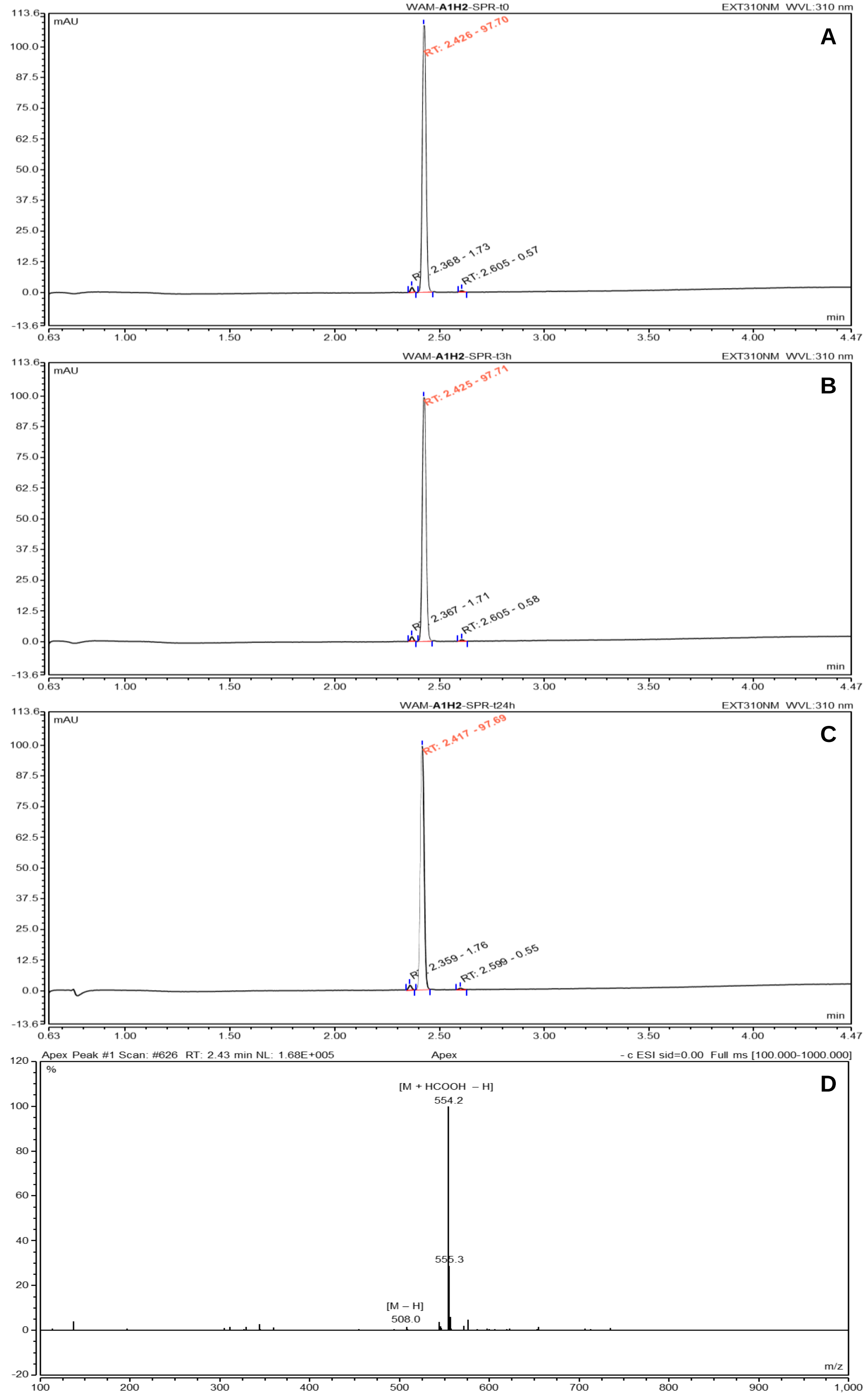

Figure S4. HPLC chromatograms of **A1H2** (t_R 2.42 min) incubated in HEPES buffer (pH 7.4) at rt for 0 (A), 3 (B), and 24 h (C) and the corresponding mass spectrum (D).

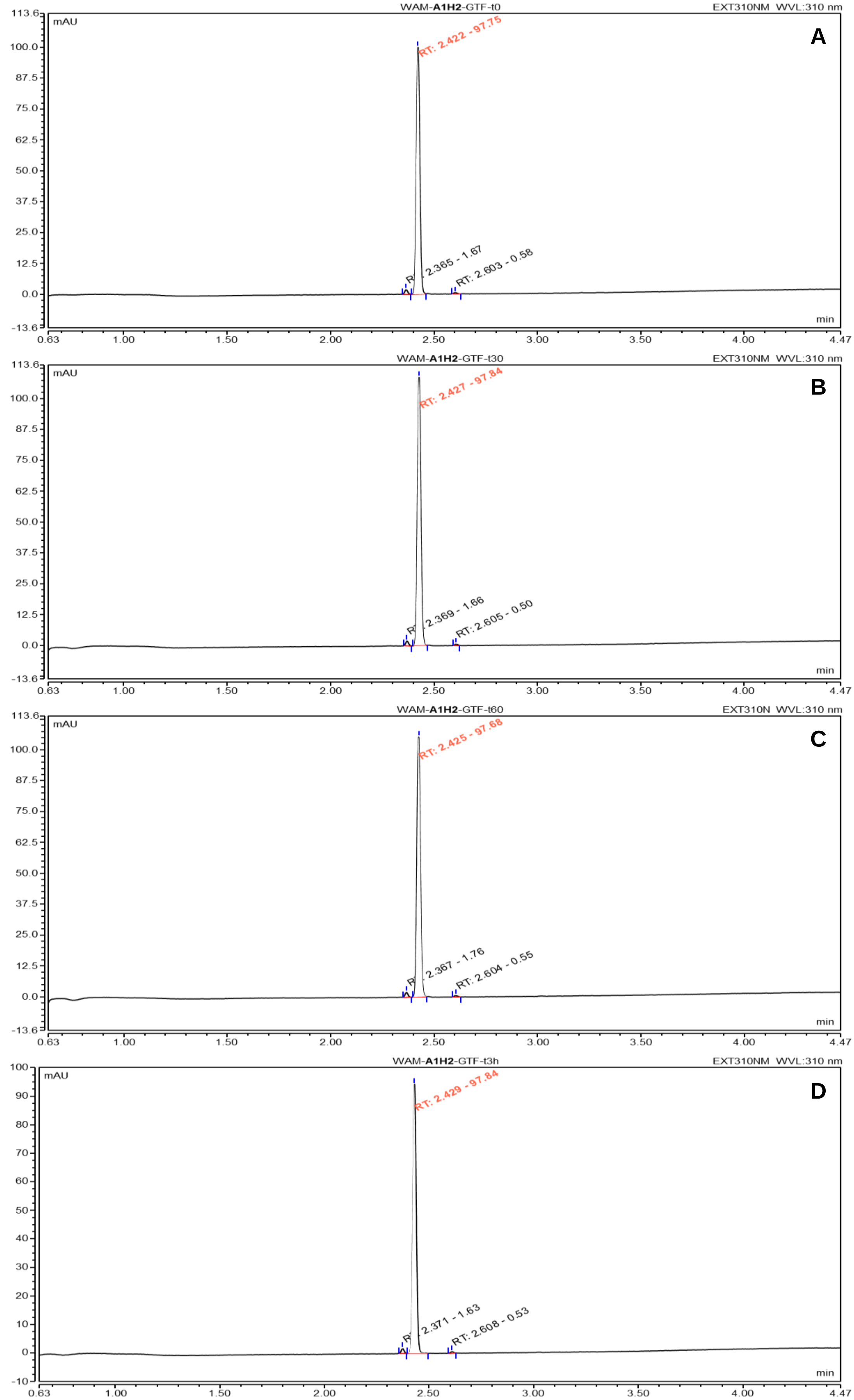

Figure S5. HPLC chromatograms of **A1H2** (t_R 2.42 min) incubated in acetate buffer (pH 4.7) at 37 °C for 0 (A), 0.5 (B), 1 (C), and 3 h (D).

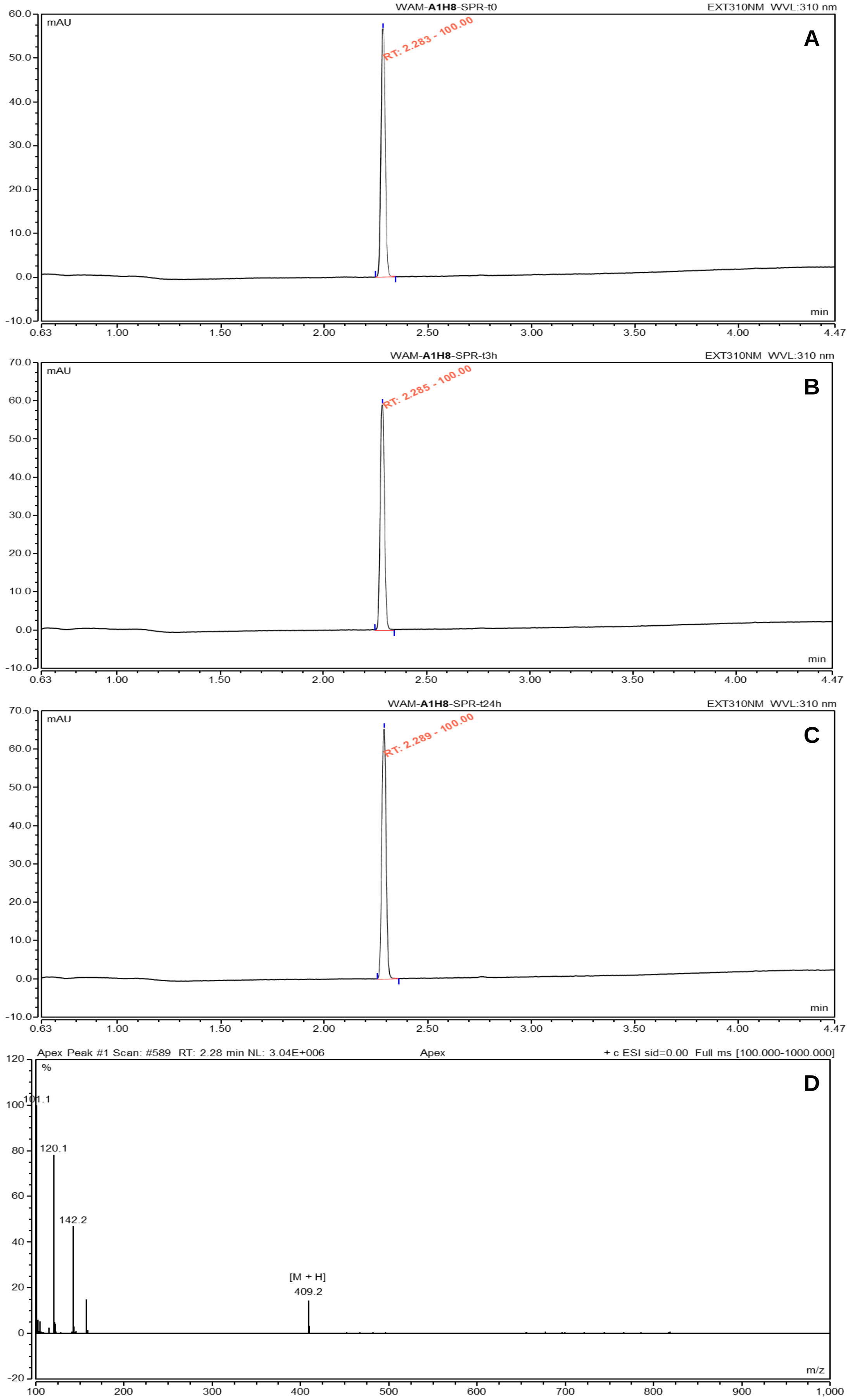

Figure S6. HPLC chromatograms of **A1H8** (t_R 2.28 min) incubated in HEPES buffer (pH 7.4) at rt for 0 (A), 3 (B), and 24 h (C) and the corresponding mass spectrum (D).

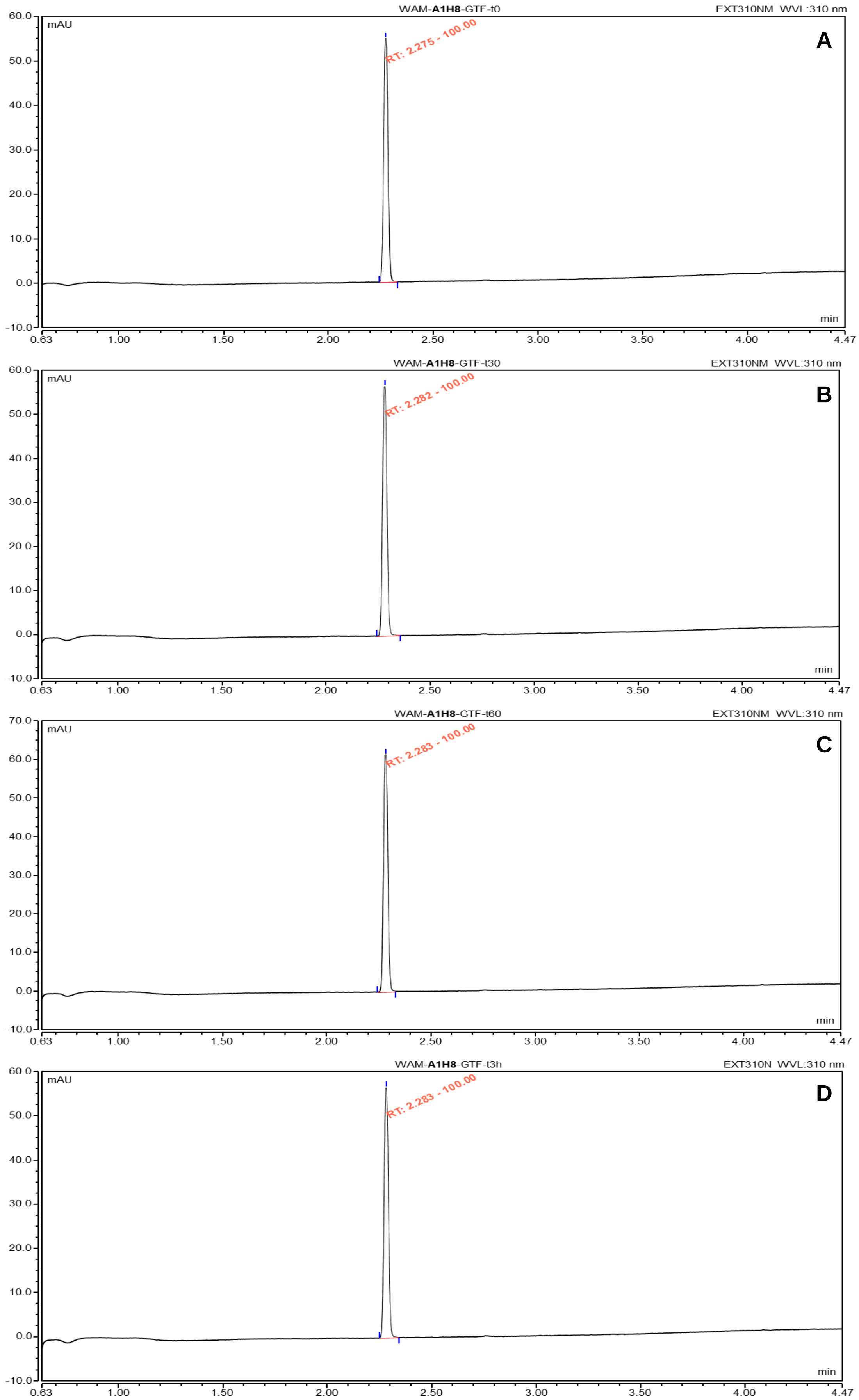

Figure S7. HPLC chromatograms of **A1H8** (t_R 2.28 min) incubated in acetate buffer (pH 4.7) at 37 °C for 0 (A), 0.5 (B), 1 (C), and 3 h (D).

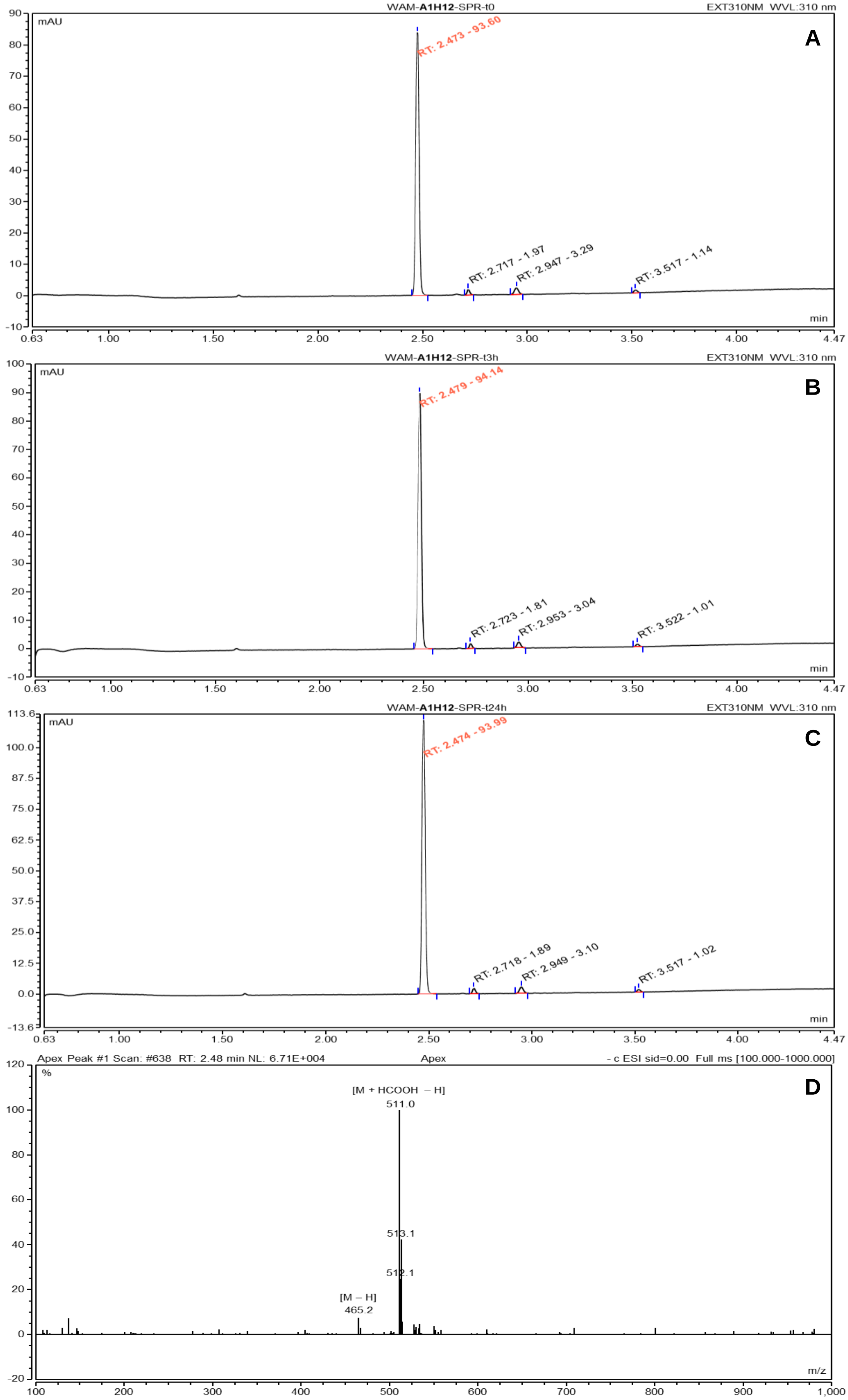

Figure S8. HPLC chromatograms of **A1H12** (t_R 2.47 min) incubated in HEPES buffer (pH 7.4) at rt for 0 (A), 3 (B), and 24 h (C) and the corresponding mass spectrum (D).

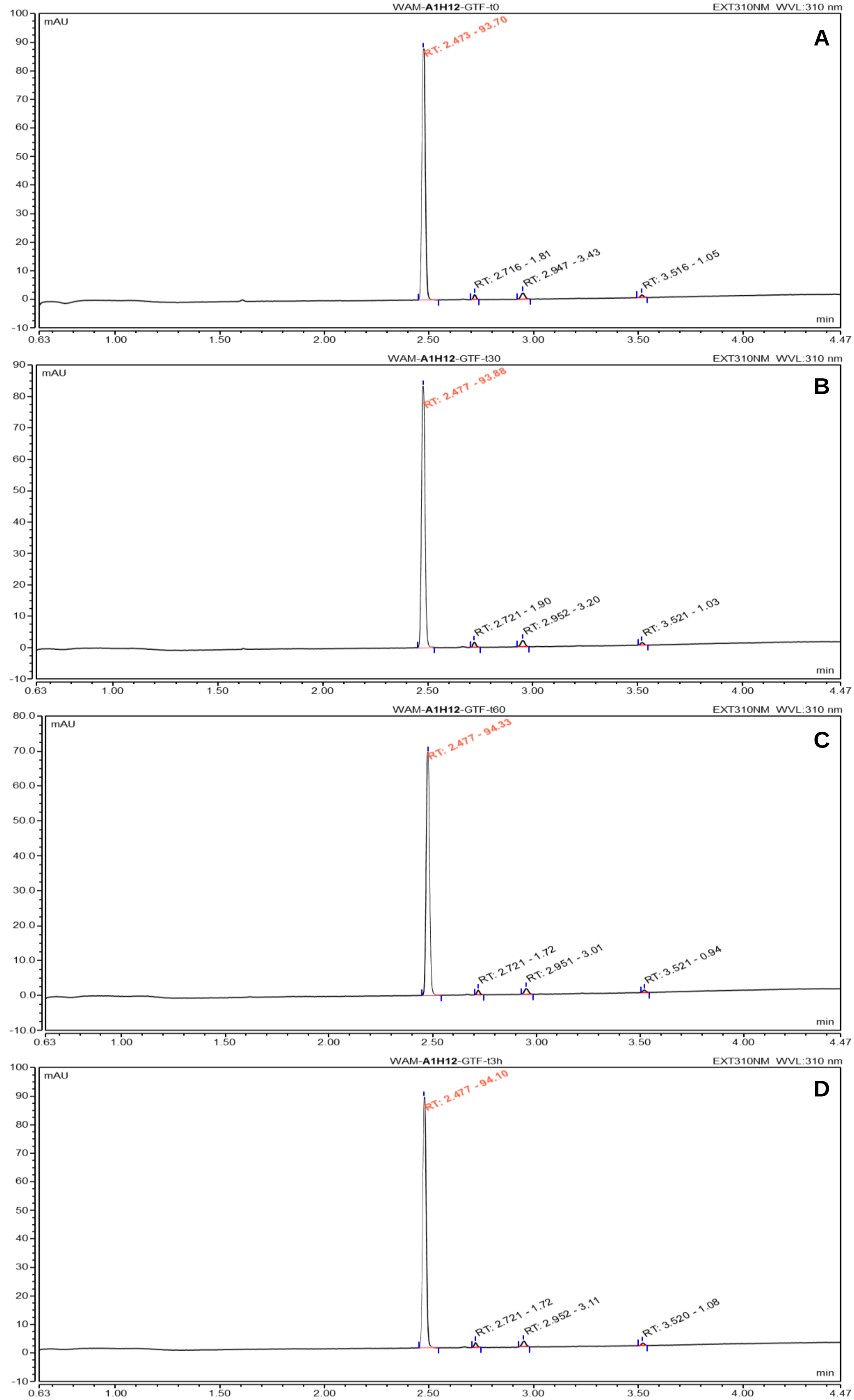

Figure S9. HPLC chromatograms of **A1H12** (t_R 2.47 min) incubated in acetate buffer (pH 4.7) at 37 °C for 0 (A), 0.5 (B), 1 (C), and 3 h (D).

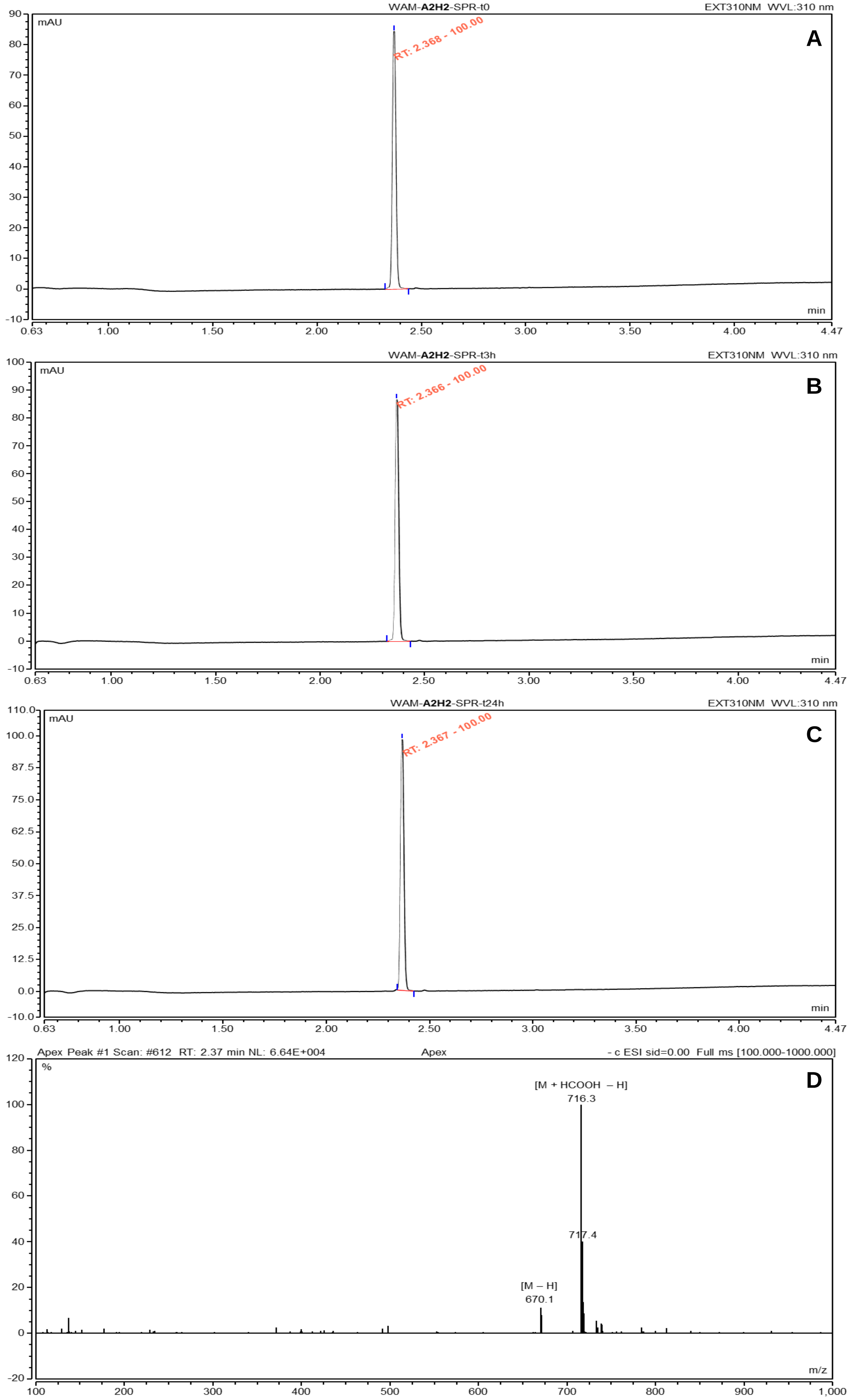

Figure S10. HPLC chromatograms of **A2H2** (t_R 2.36 min) incubated in HEPES buffer (pH 7.4) at rt for 0 (A), 3 (B), and 24 h (C) and the corresponding mass spectrum (D).

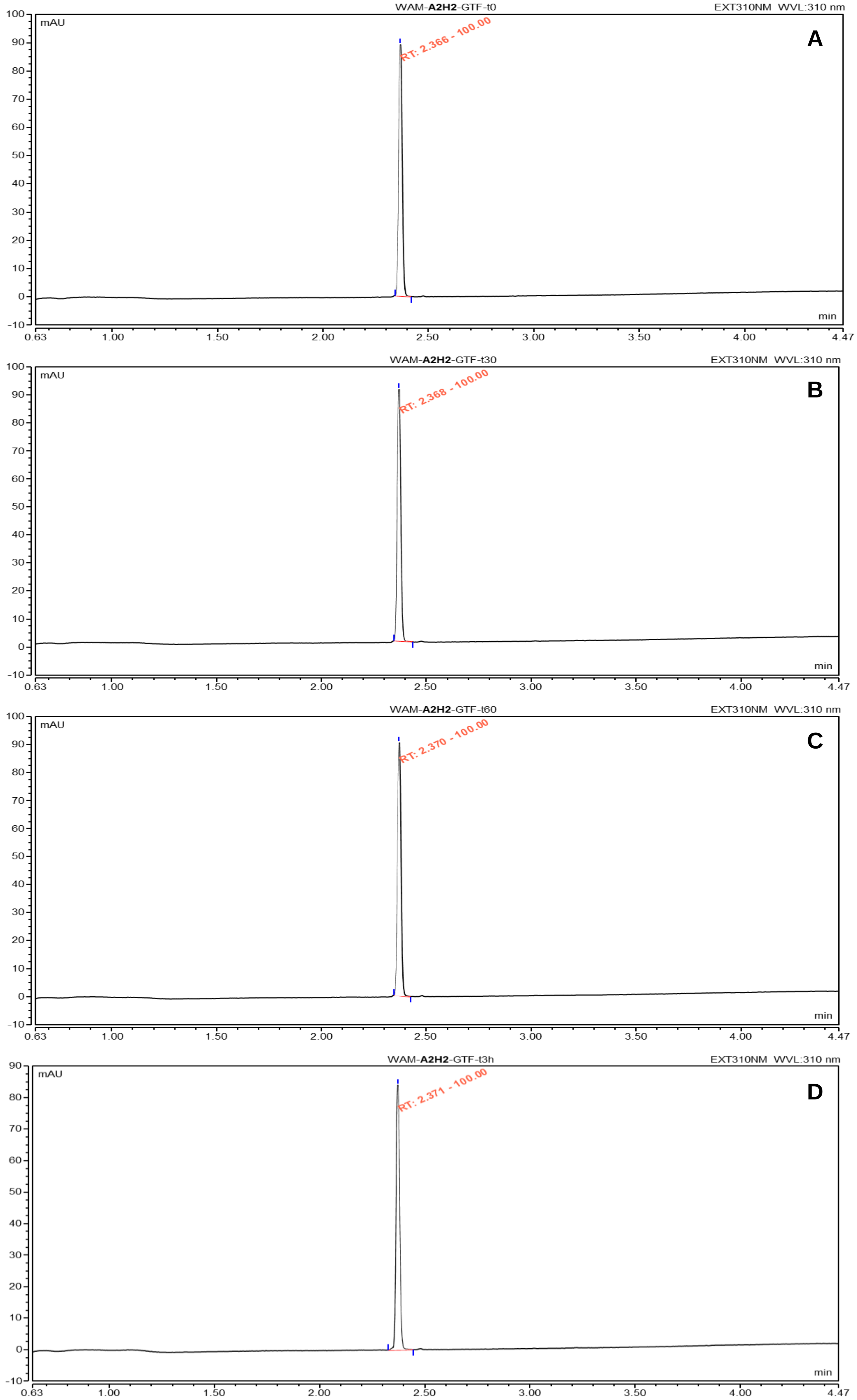

Figure S11. HPLC chromatograms of **A2H2** (t_R 2.37 min) incubated in acetate buffer (pH 4.7) at 37 °C for 0 (A), 0.5 (B), 1 (C), and 3 h (D).

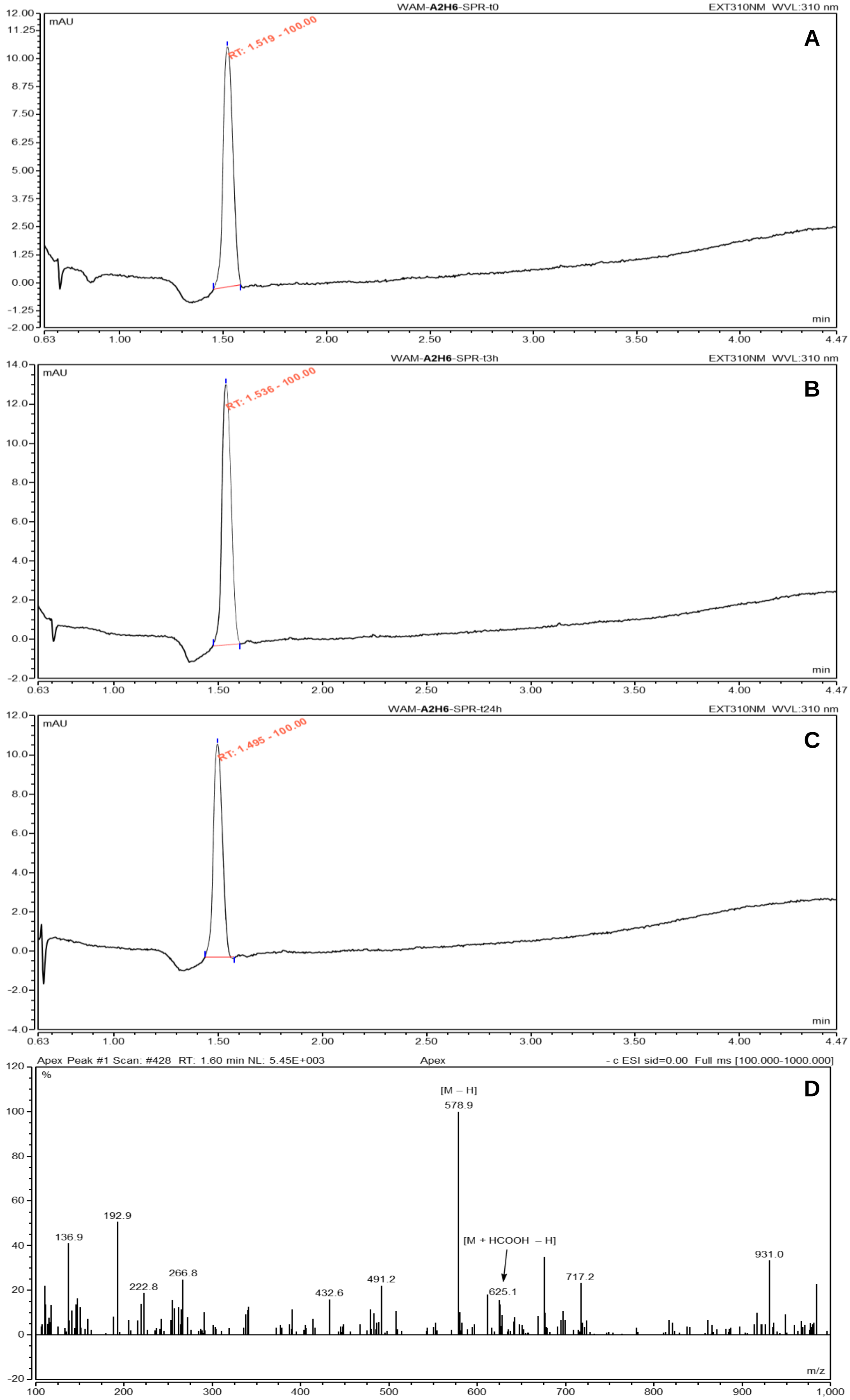

Figure S12. HPLC chromatograms of **A2H6** (t_R 1.53 min) incubated in HEPES buffer (pH 7.4) at rt for 0 (A), 3 (B), and 24 h (C) and the corresponding mass spectrum (D).

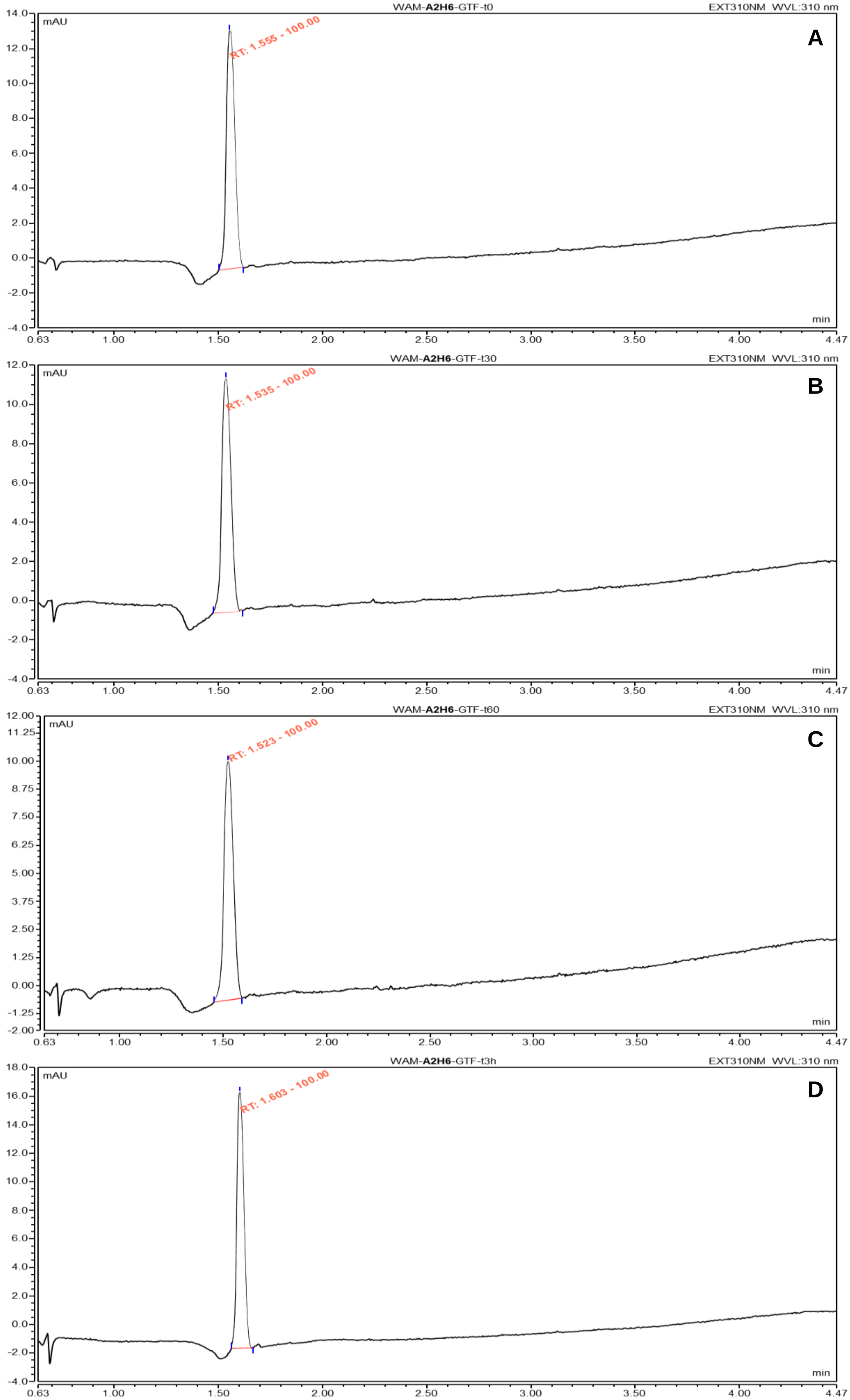

Figure S13. HPLC chromatograms of **A2H6** (t_R 1.53 min) incubated in acetate buffer (pH 4.7) at 37 °C for 0 (A), 0.5 (B), 1 (C), and 3 h (D).

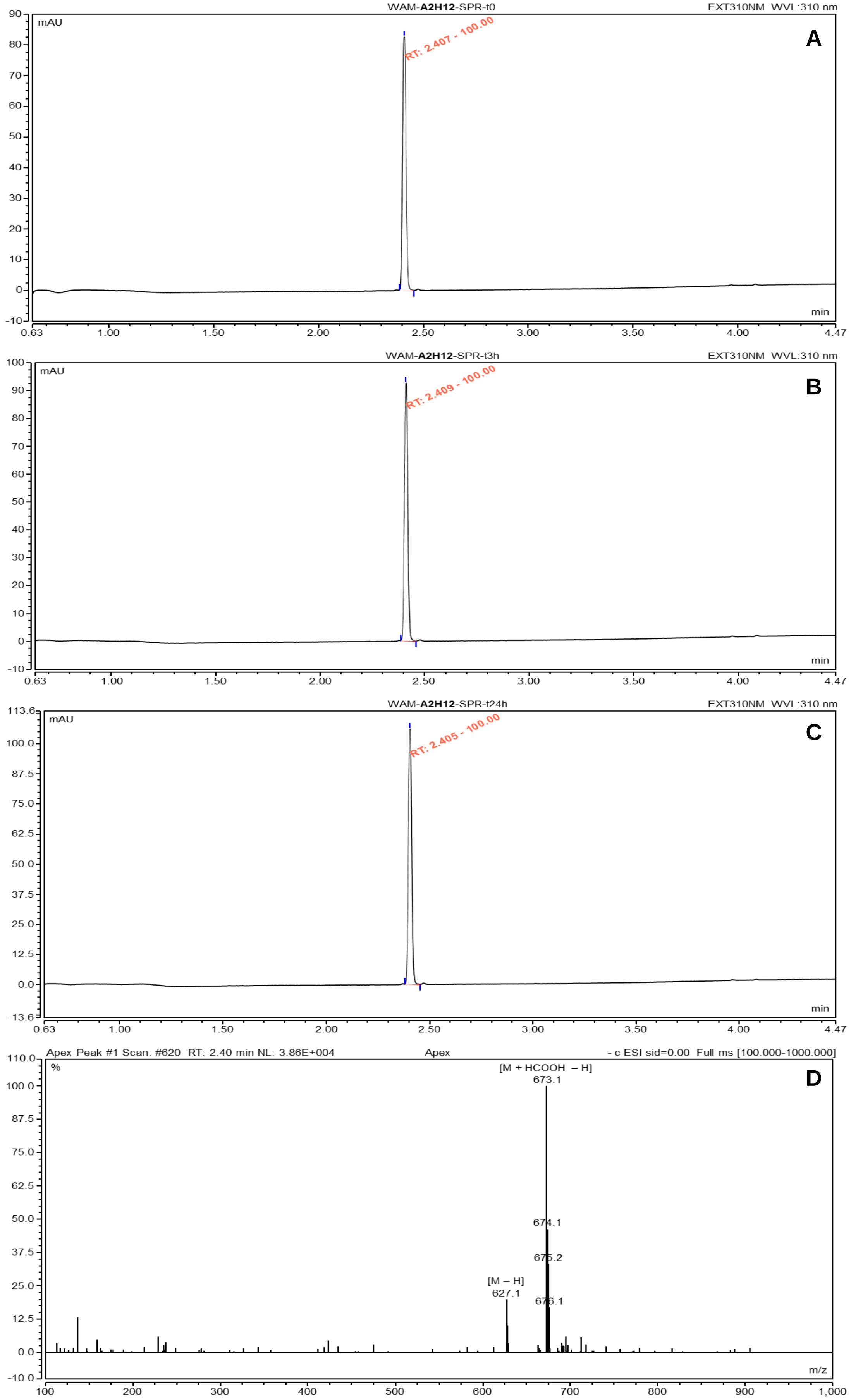

Figure S14. HPLC chromatograms of **A2H12** (t_R 2.40 min) incubated in HEPES buffer (pH 7.4) at rt for 0 (A), 3 (B), and 24 h (C) and the corresponding mass spectrum (D).

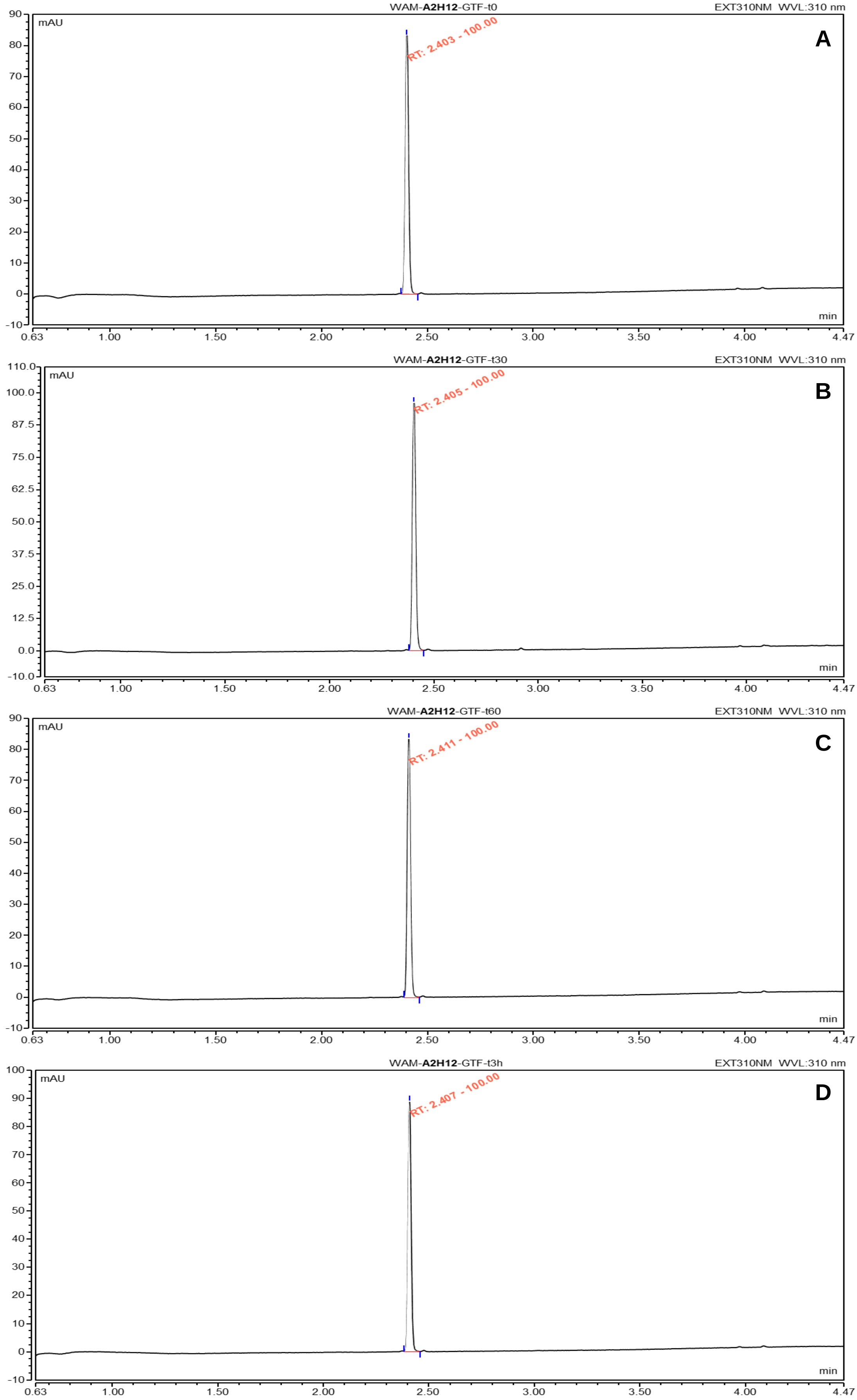

Figure S15. HPLC chromatograms of **A2H12** (t_R 2.40 min) incubated in acetate buffer (pH 4.7) at 37 °C for 0 (A), 0.5 (B), 1 (C), and 3 h (D).

Binding studies by surface plasmon resonance (SPR)

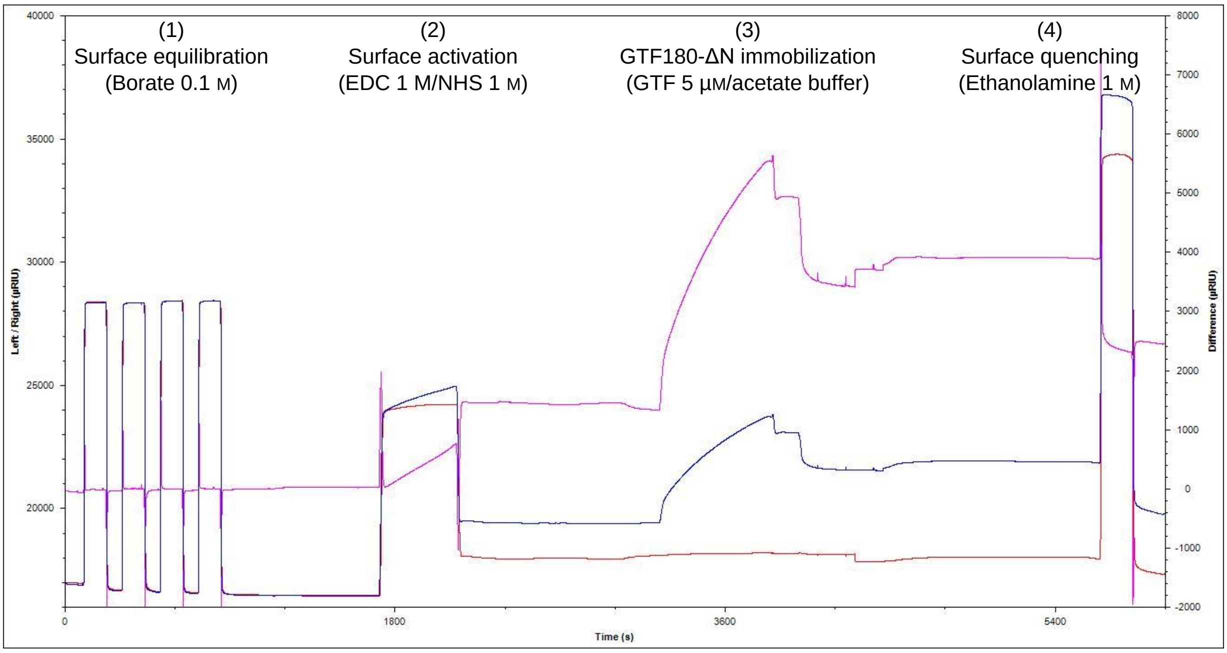

Figure S16. Sensorgram of the immobilization procedure for GTF180-ΔN on CMD500M sensor chip: (1) Four injections of cleaning solution, (2) activation solution, (3) GTF180-ΔN, and (4) quenching solution. The blue, red, and magenta curves represent the left (active) channel, right (reference) channel, and the difference, respectively.

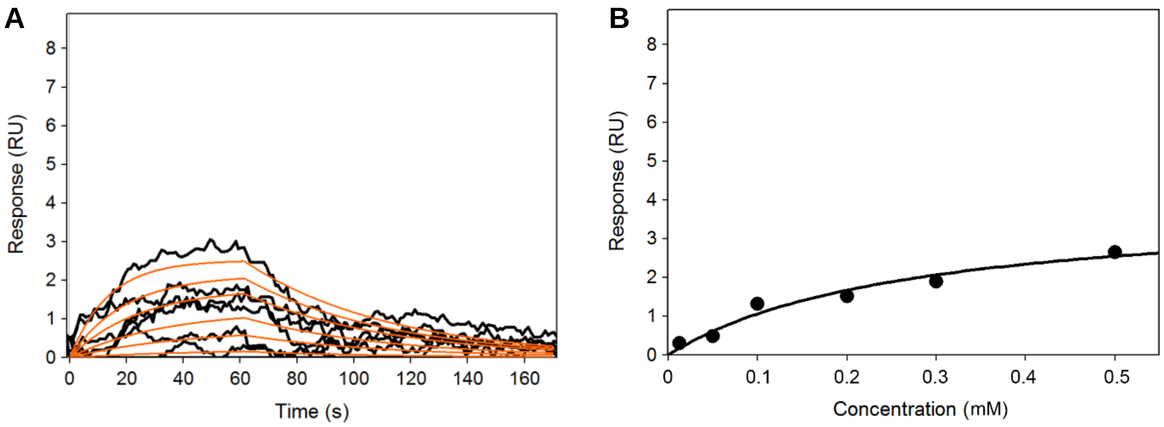

Figure S17. (A) Overlay of sensorgrams (black) of acarbose injected at concentrations of 0.0125–0.5 mM over an immobilized GTF-180. Global fitting of the association and dissociation curves (red); (B) Fitting of responses at equilibrium according to 1:1 Langmuir binding model (K_D: 0.18 mM).

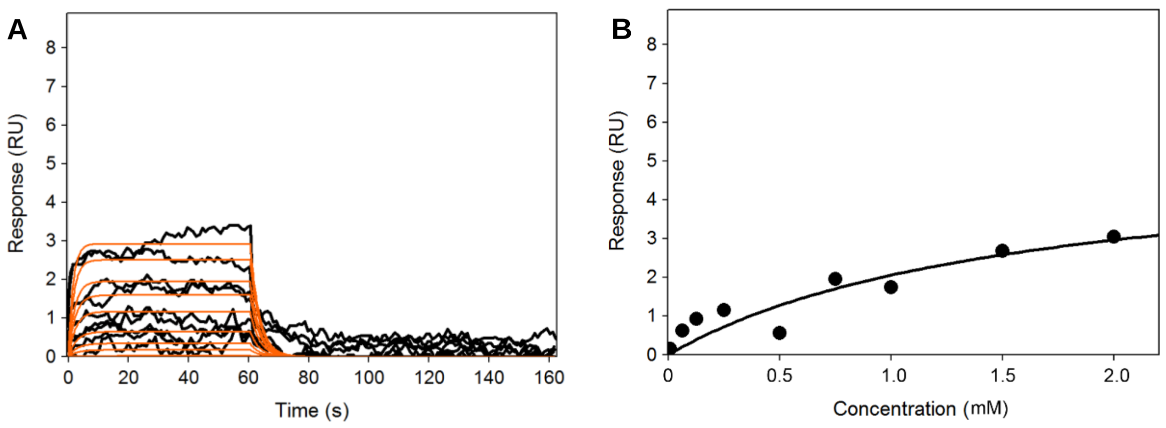

Figure S18. (A) Overlay of sensorgrams (black) of **A1H2** injected at concentrations of 0.0076–2.0 mM over an immobilized GTF-180. Global fitting of the association and dissociation curves (red); (B) Fitting of responses at equilibrium according to 1:1 Langmuir binding model (K_D: 1.6 mM).

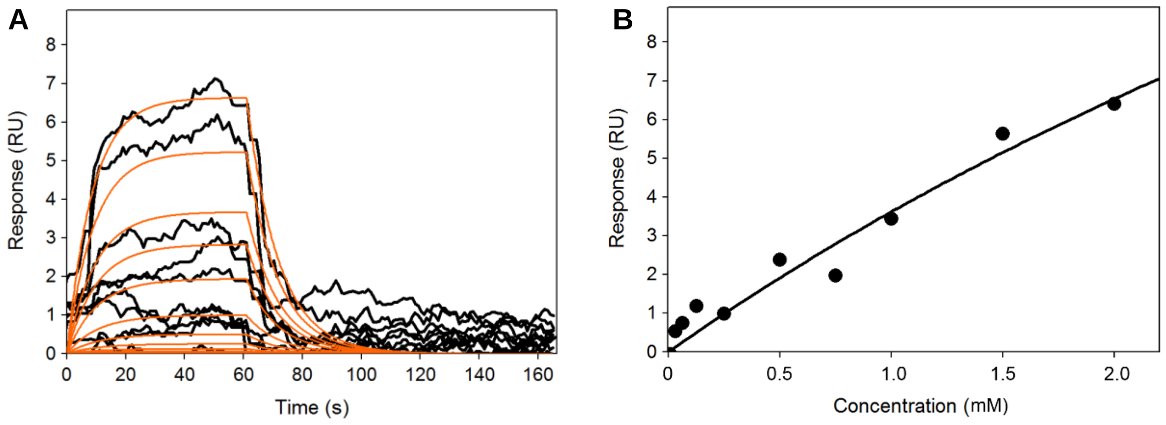

Figure S19. (A) Overlay of sensorgrams (black) of **A1H8** injected at concentrations of 0.0076–2.0 mM over an immobilized GTF-180. Global fitting of the association and dissociation curves (red); (B) Fitting of responses at equilibrium according to 1:1 Langmuir binding model (K_D: 8.0 mM).

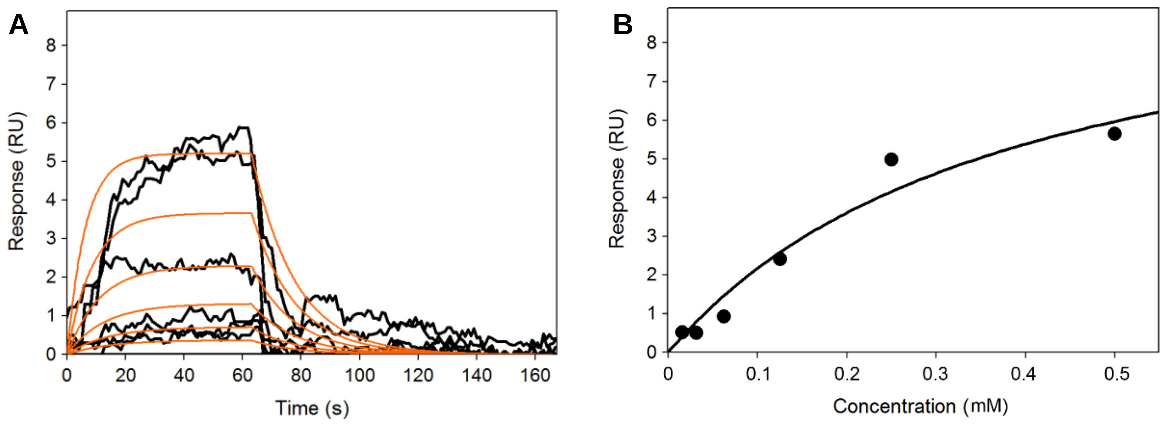

Figure S20. (A) Overlay of sensorgrams (black) of **A1H12** injected at concentrations of 0.0156–0.5 mM over an immobilized GTF-180. Global fitting of the association and dissociation curves (red); (B) Fitting of responses at equilibrium according to 1:1 Langmuir binding model (K_D: 0.4 mM).

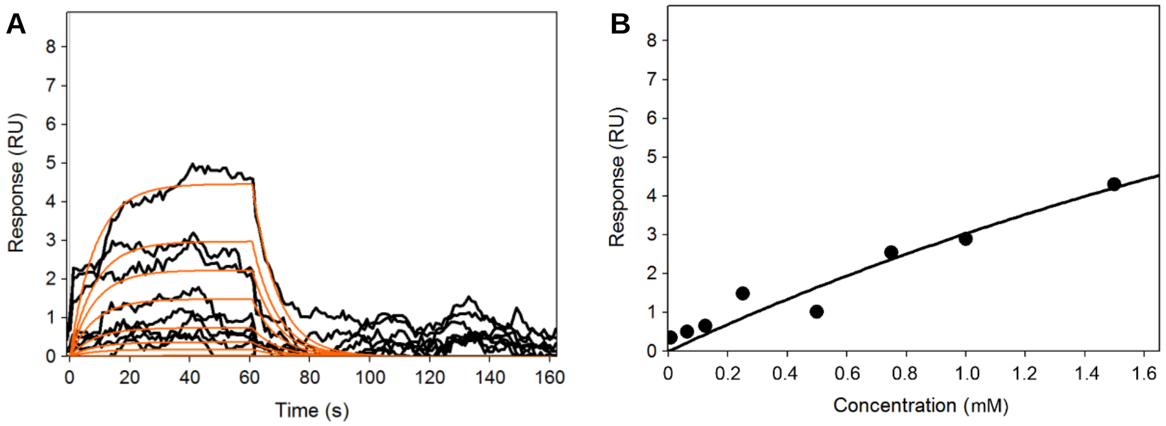

Figure S21. (A) Overlay of sensorgrams (black) of **A2H2** injected at concentrations of 0.0076–1.5 mM over an immobilized GTF-180. Global fitting of the association and dissociation curves (red); (B) Fitting of responses at equilibrium according to 1:1 Langmuir binding model (K_D: 5.0 mM).

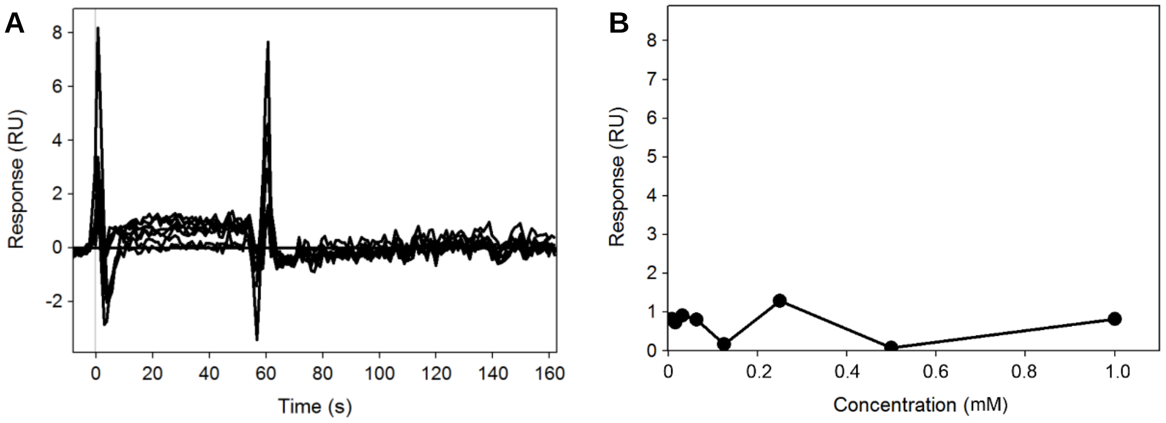

Figure S22. (A) Overlay of sensorgrams of **A2H6** injected at concentrations of 0.0076–1.0 mM over an immobilized GTF-180 showing low responses (RU < 2) indicating no or a very weak binding; (B) Responses at equilibrium could not be fitted to 1:1 Langmuir binding model.

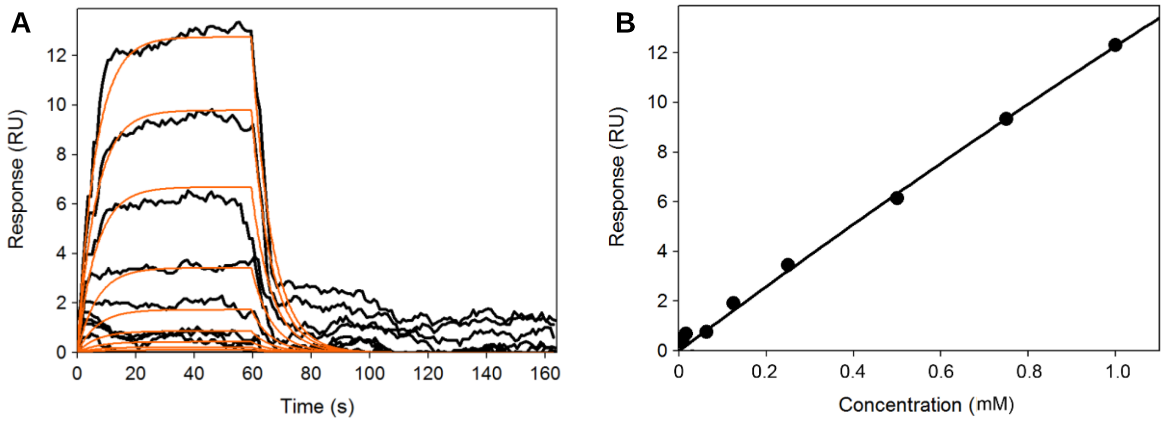

Figure S23. (A) Overlay of sensorgrams (black) of **A2H12** injected at concentrations of 0.0076–1.0 mM over an immobilized GTF-180. Global fitting of the association and dissociation curves (red); (B) Fitting of responses at equilibrium according to 1:1 Langmuir binding model (K_D: 10.0 mM).

GTF180-ΔN activity assay

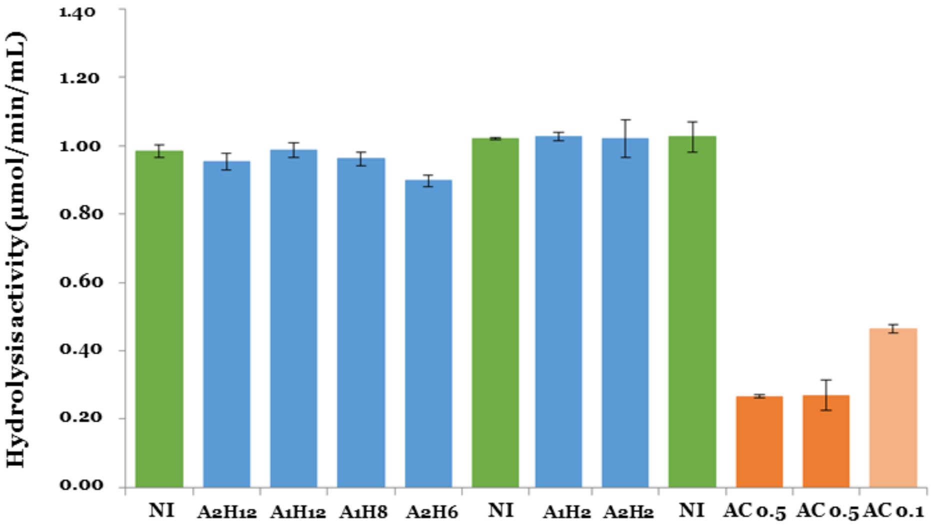

Figure S24. GTF180-ΔN activity assay based on the hydrolysis of sucrose (10 mM). No significant inhibition was observed for the hit compounds at 500 µM, and moderate inhibition by acarbose at 500 µM.

LC-MS analysis and NMR spectra

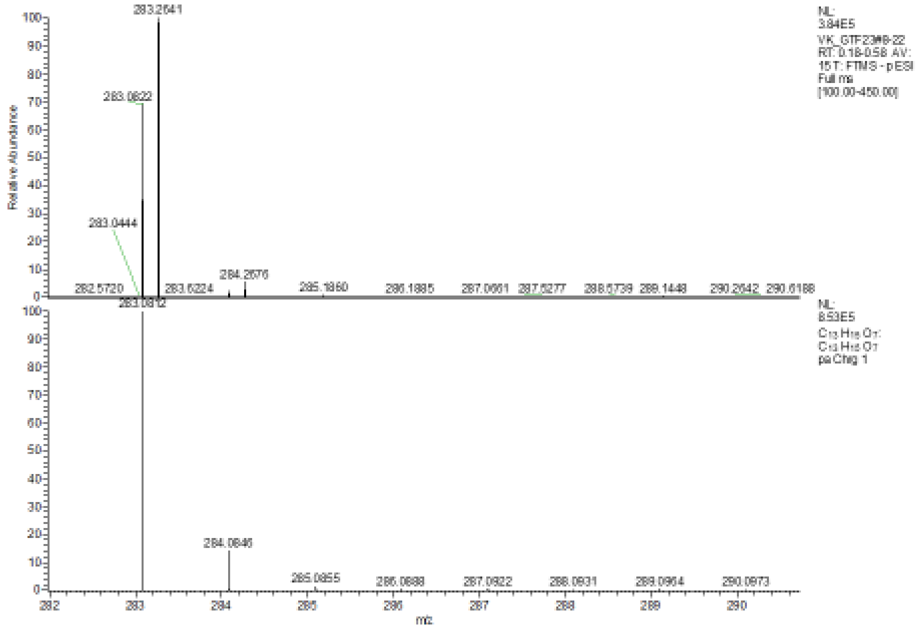

HRMS of **A1**

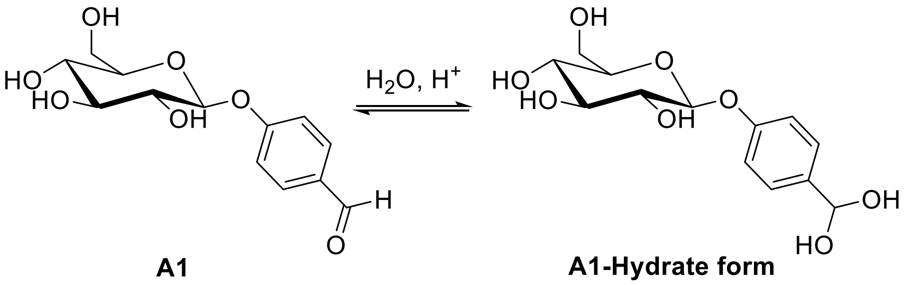

A1

Exact Mass: 284.0896

A1-Hydrate form

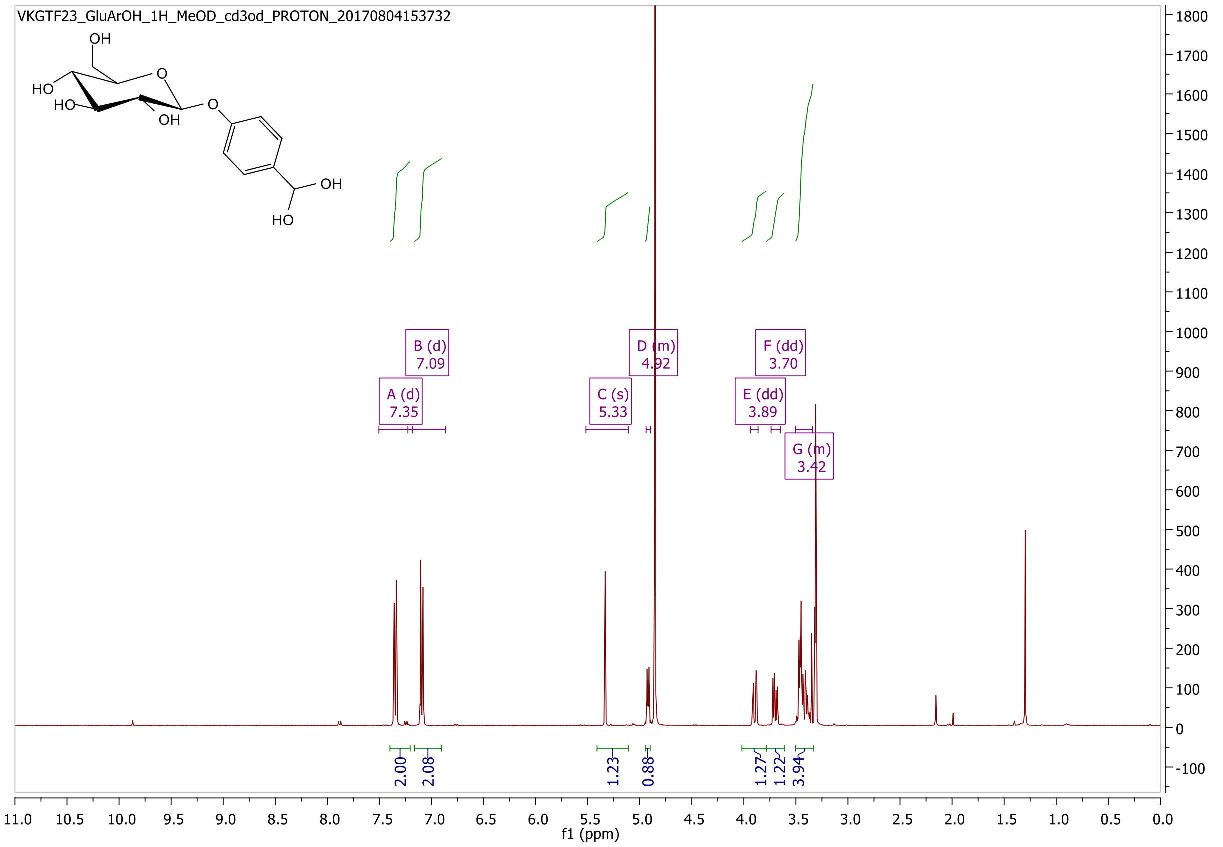

^{1}H-NMR spectrum of **A1** (in hydrate form)

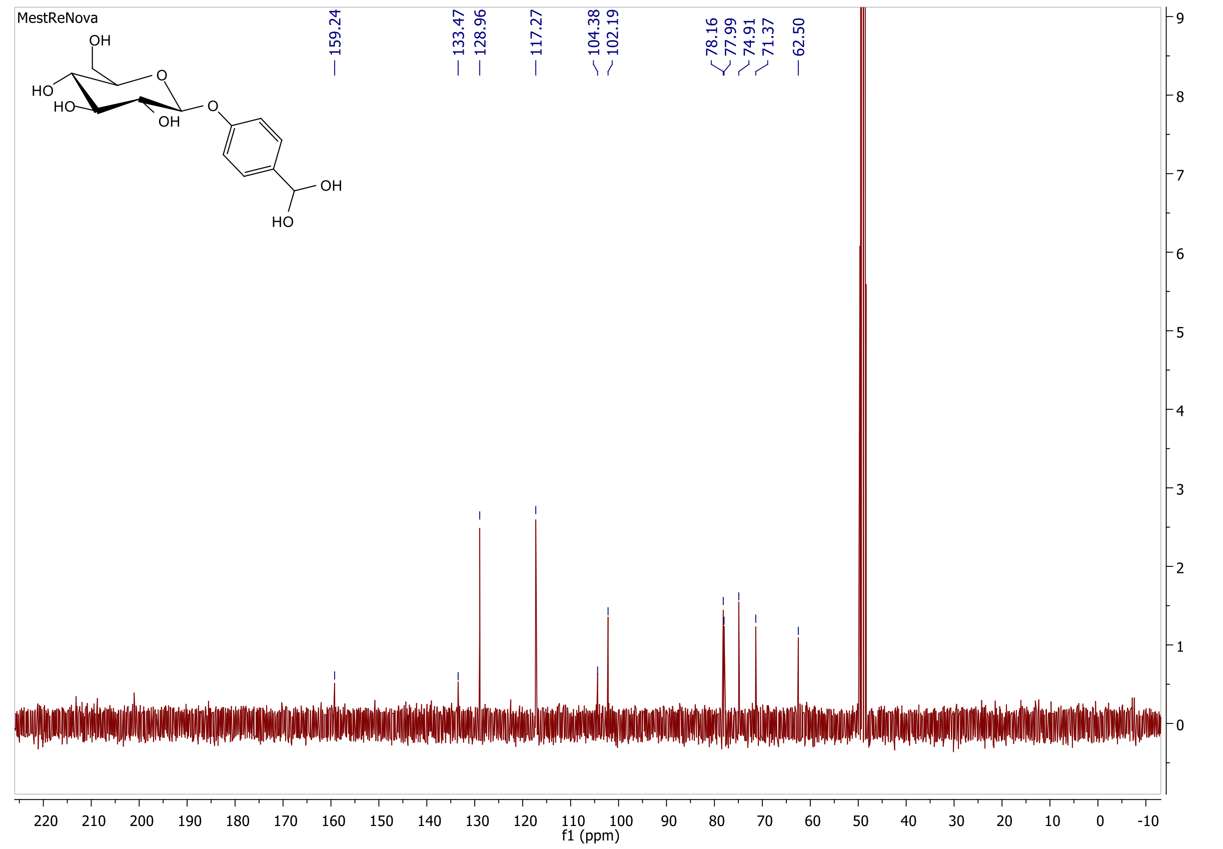

^{13}C-NMR spectrum of **A1** (in hydrate form)

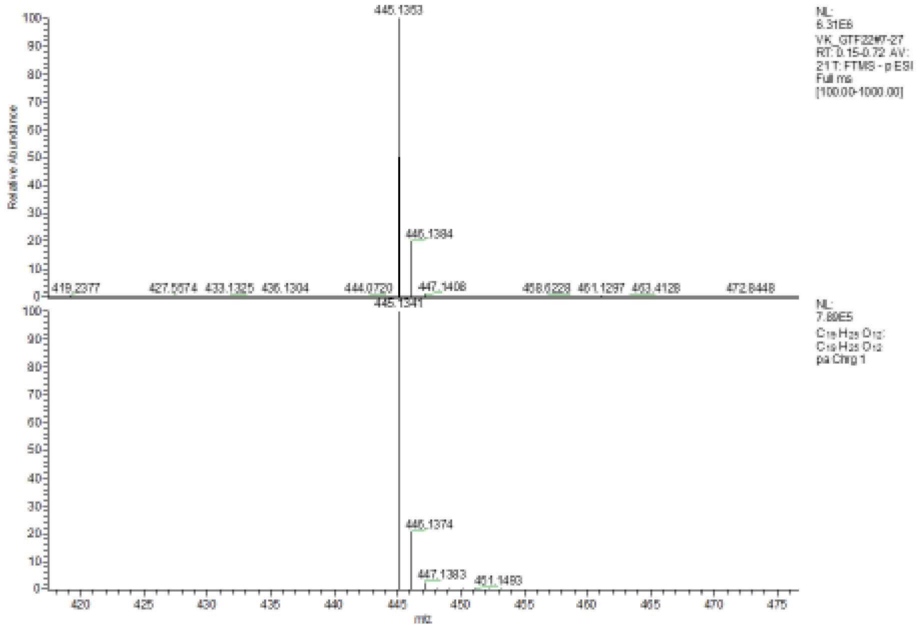

HRMS of **A2**

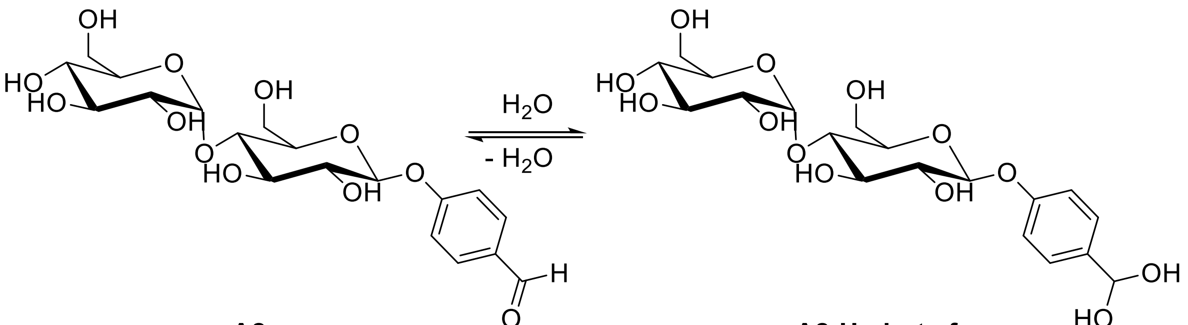

A2

Exact Mass: 446.1424

A2-Hydrate form

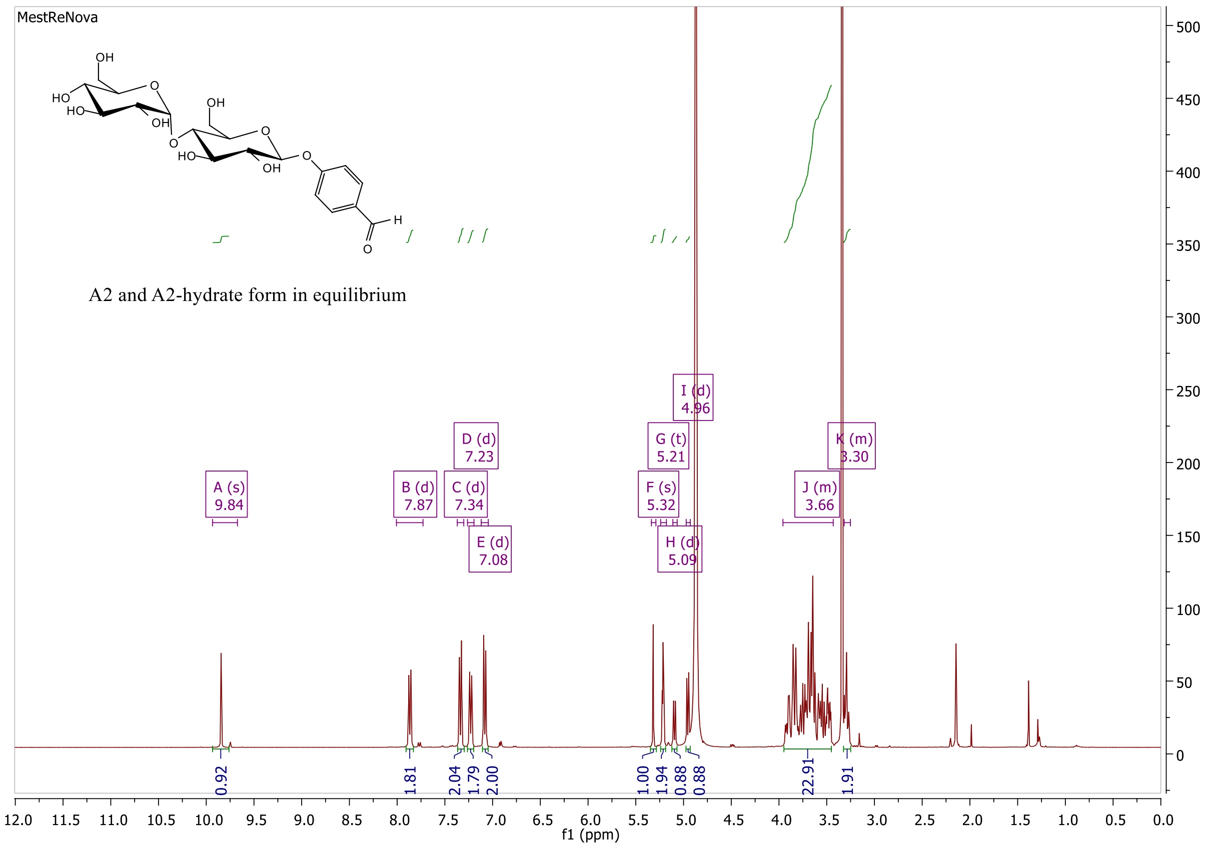

^{1}H-NMR spectrum of **A2** (in equilibrium with its hydrate form)

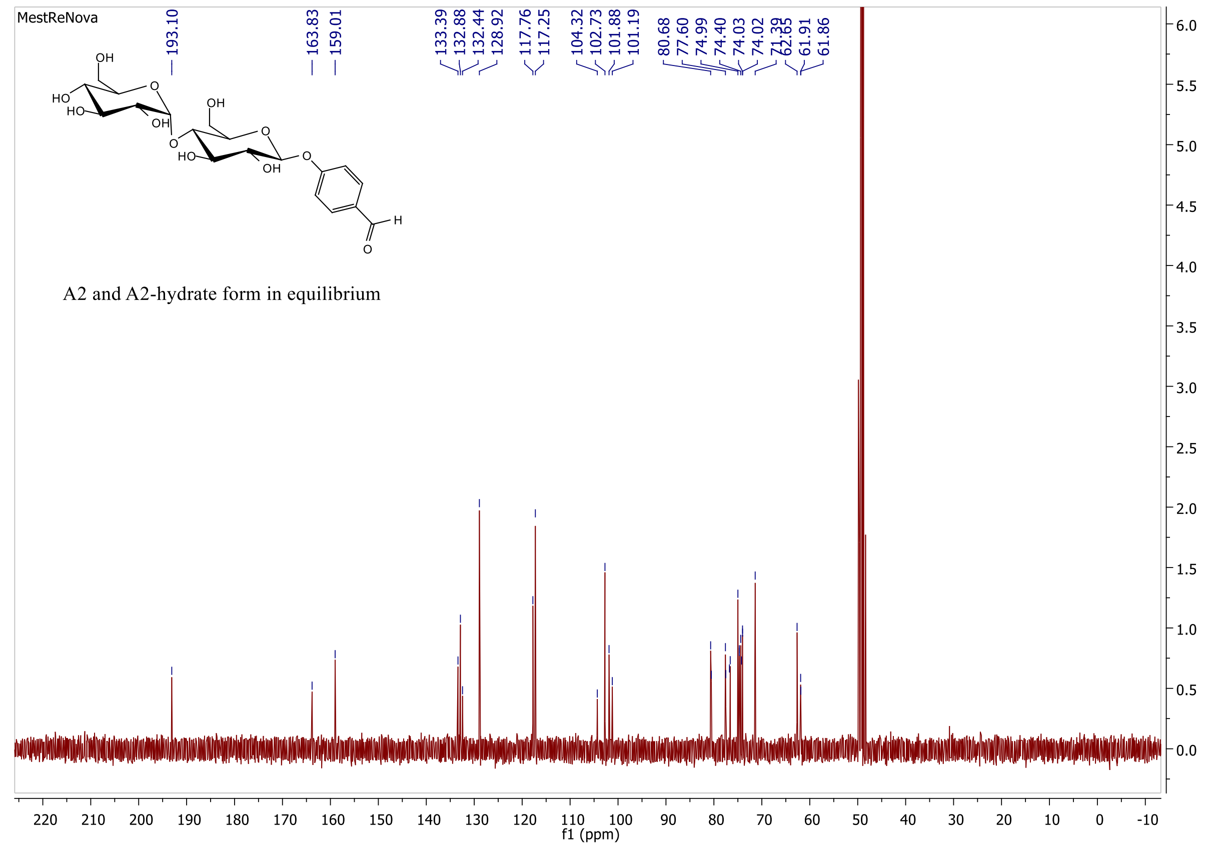

^{13}C-NMR spectrum of **A2** (in equilibrium with its hydrate form)

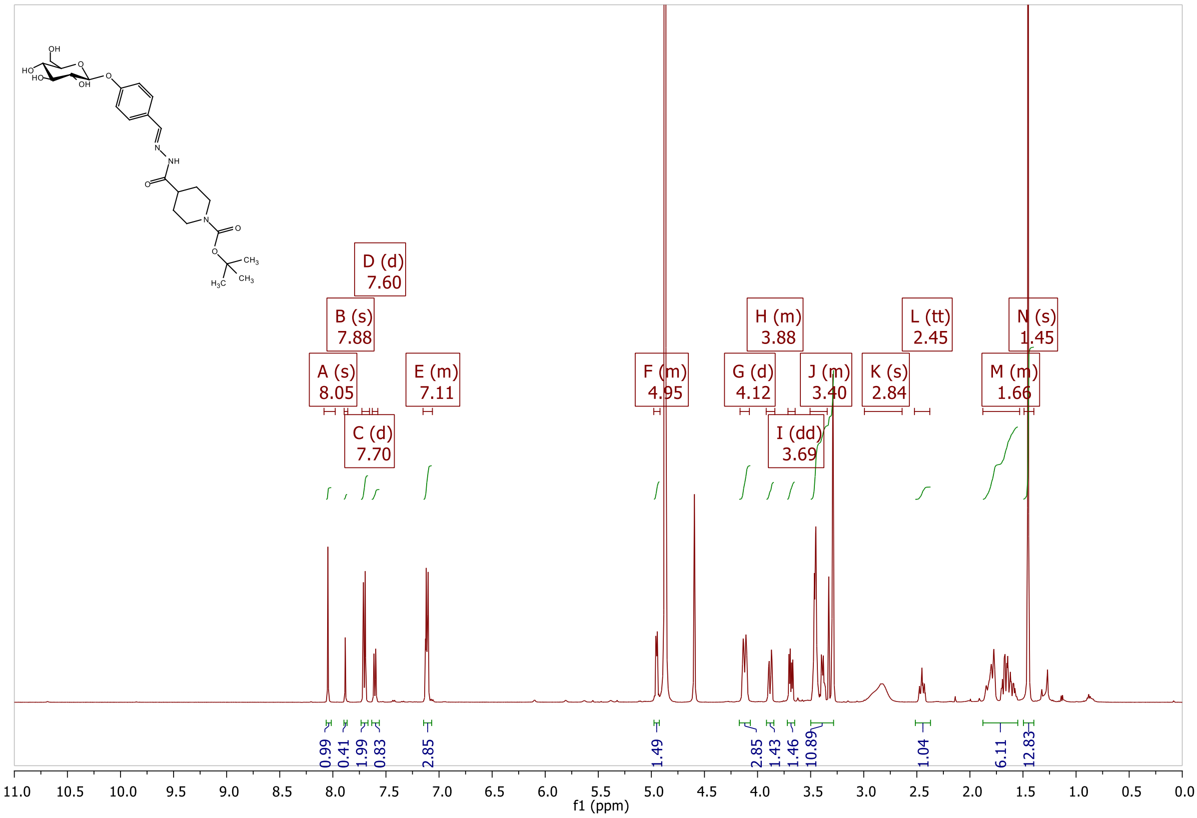

^{1}H-NMR spectrum of **A1H2**

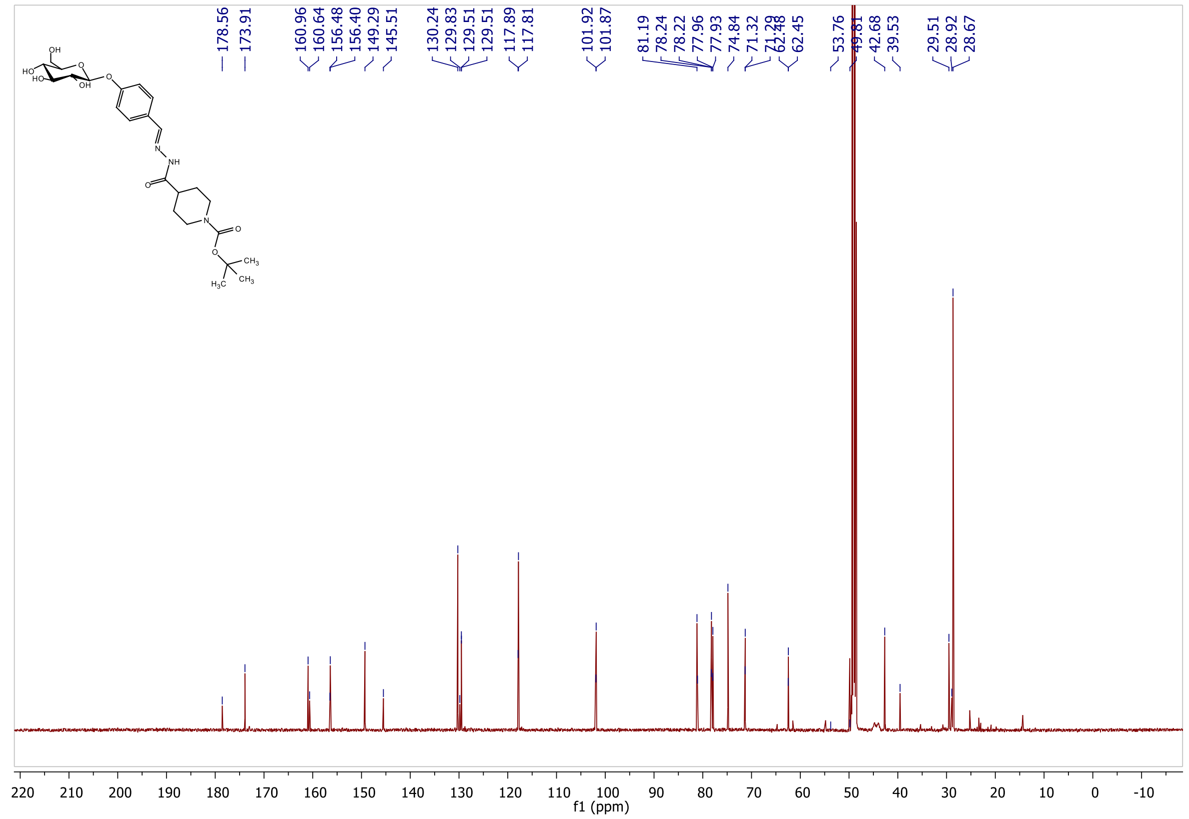

^{13}C-NMR spectrum of **A1H2**

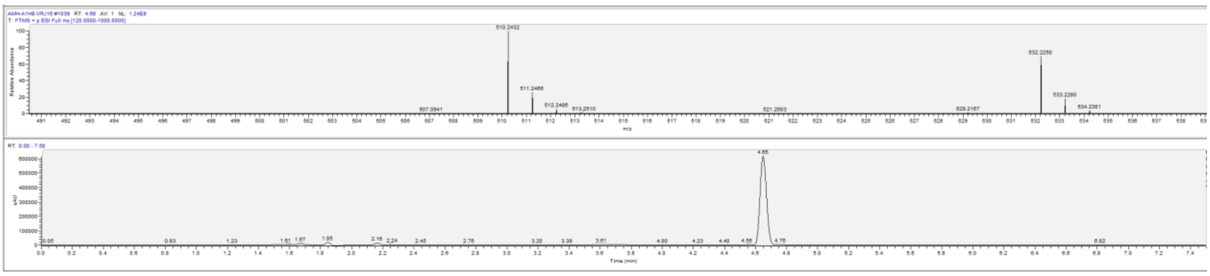

HPLC-HRMS analysis of **A1H2**, > 95% pure.

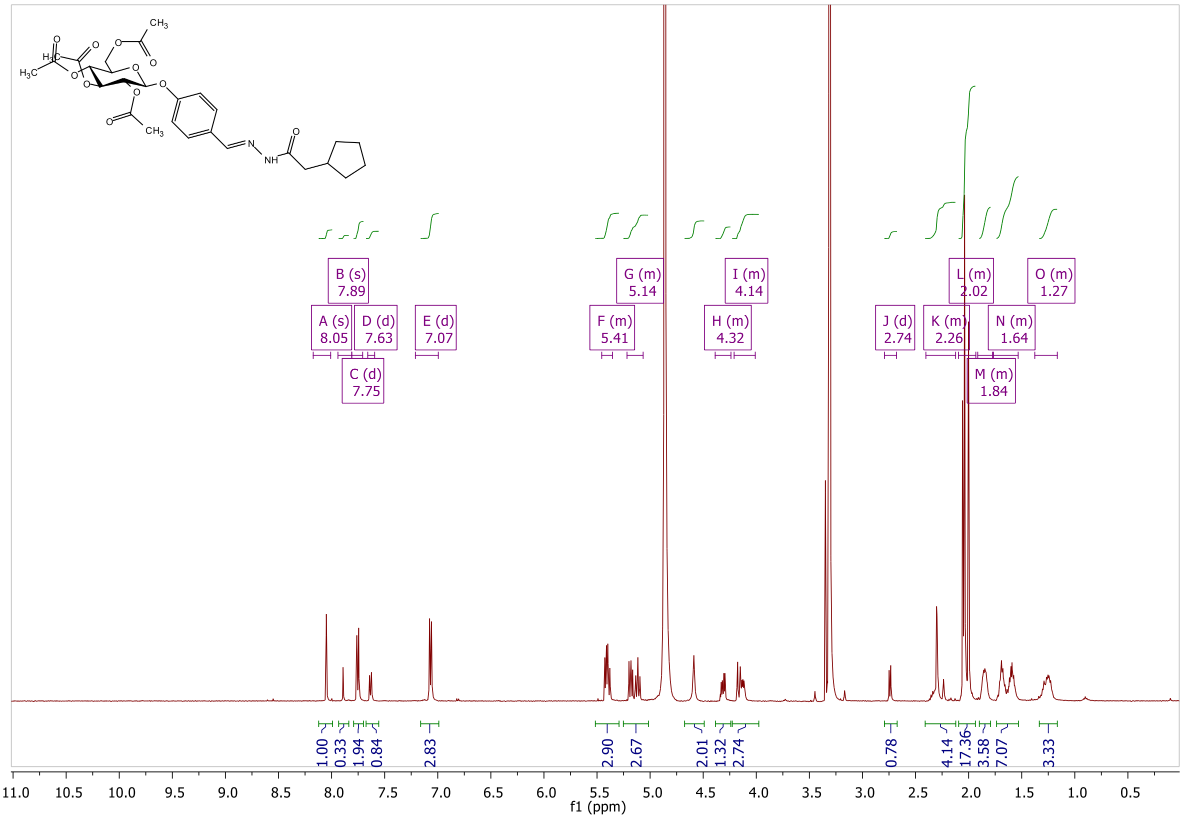

^{1}H-NMR spectrum of **acetylated A1H8**

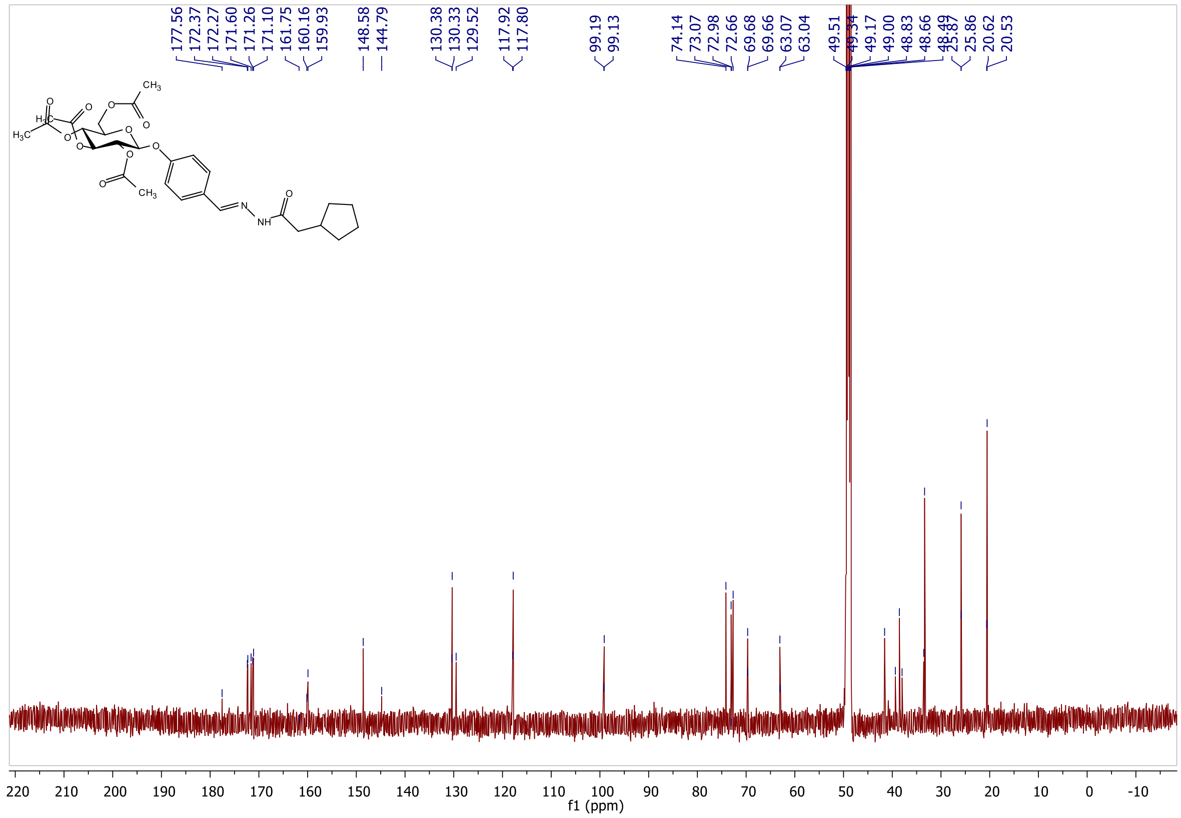

^{13}C-NMR spectrum of **acetylated A1H8**

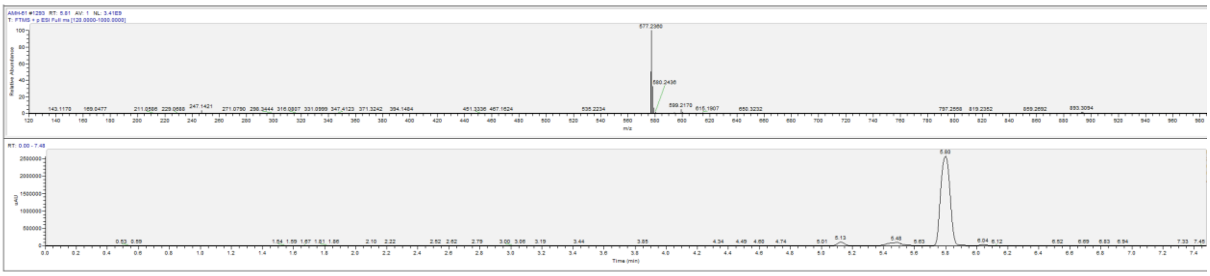

HPLC-HRMS analysis of **acetylated A1H8**, > 95% pure.

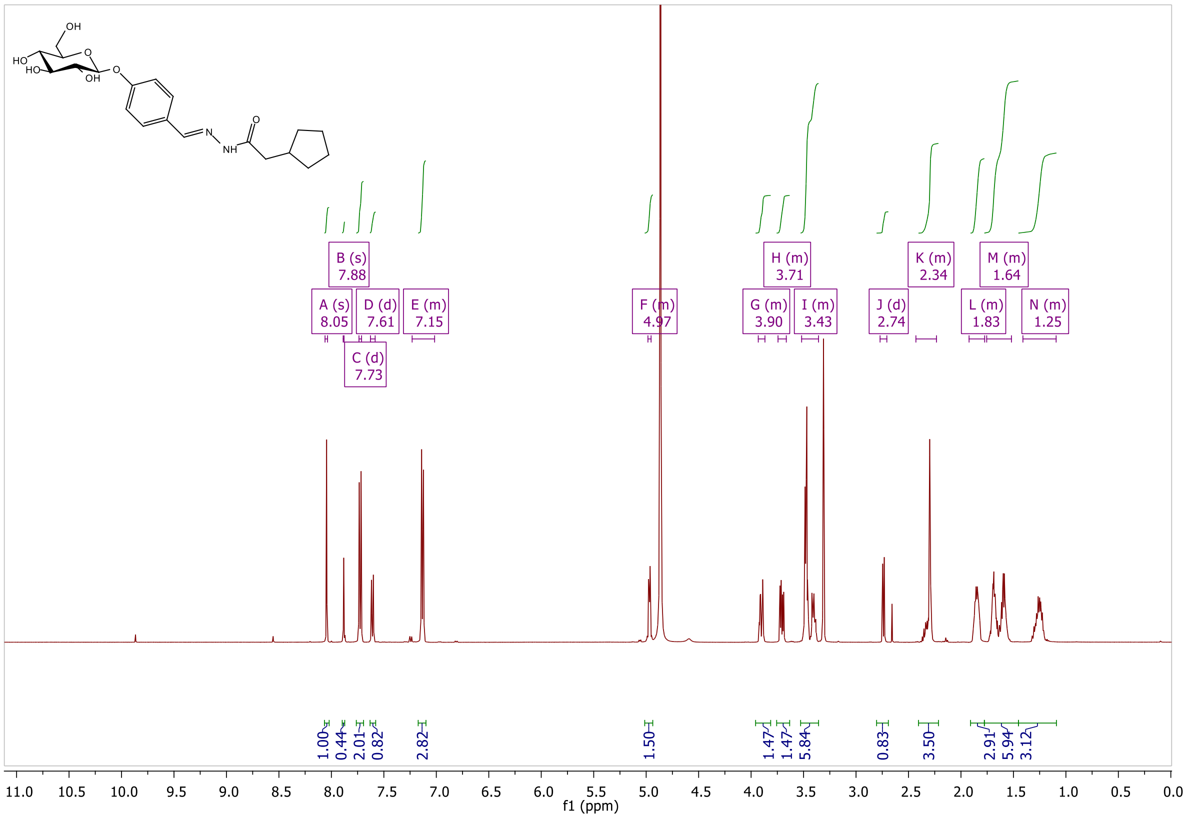

^{1}H-NMR spectrum of **A1H8**

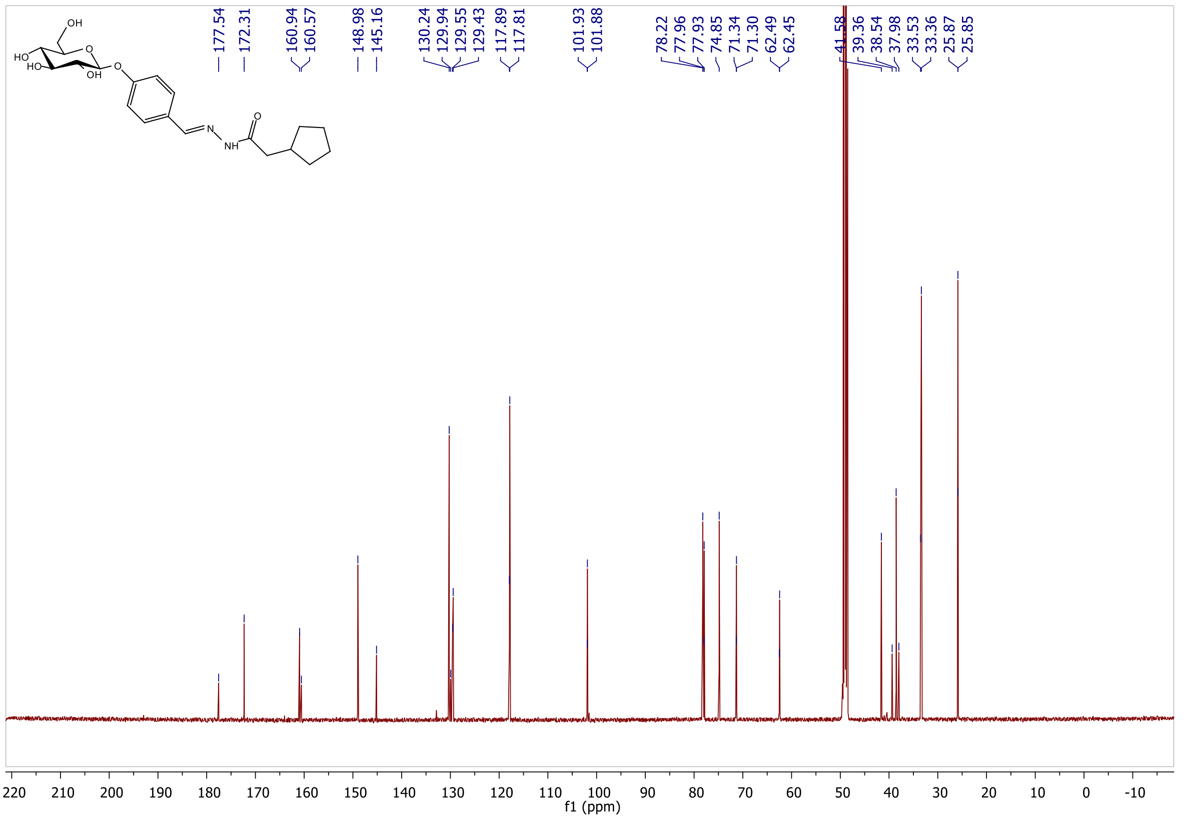

^{13}C-NMR spectrum of **A1H8**

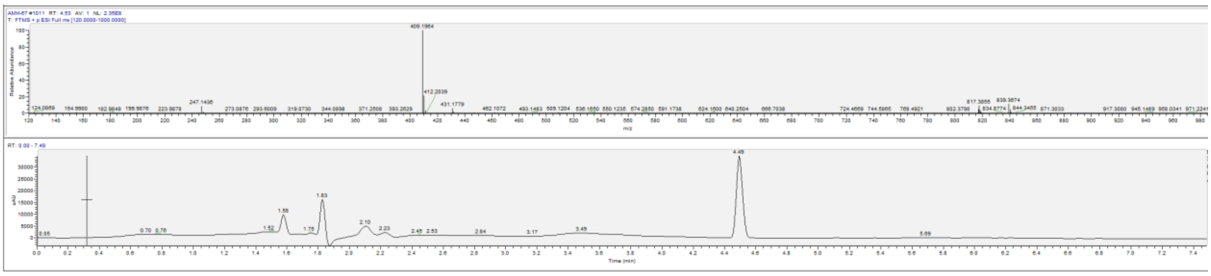

HPLC-HRMS analysis of **A1H8**, > 95% pure.

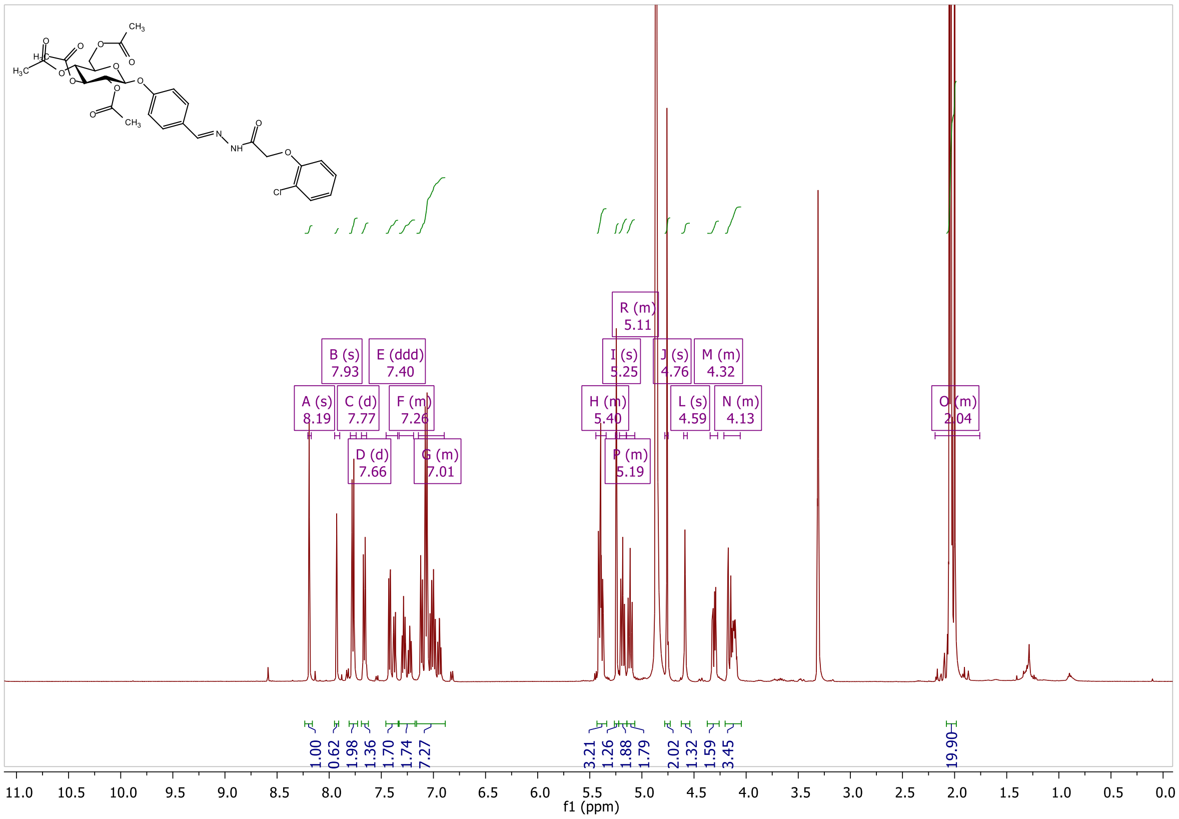

^{1}H-NMR spectrum of **acetylated A1H12**

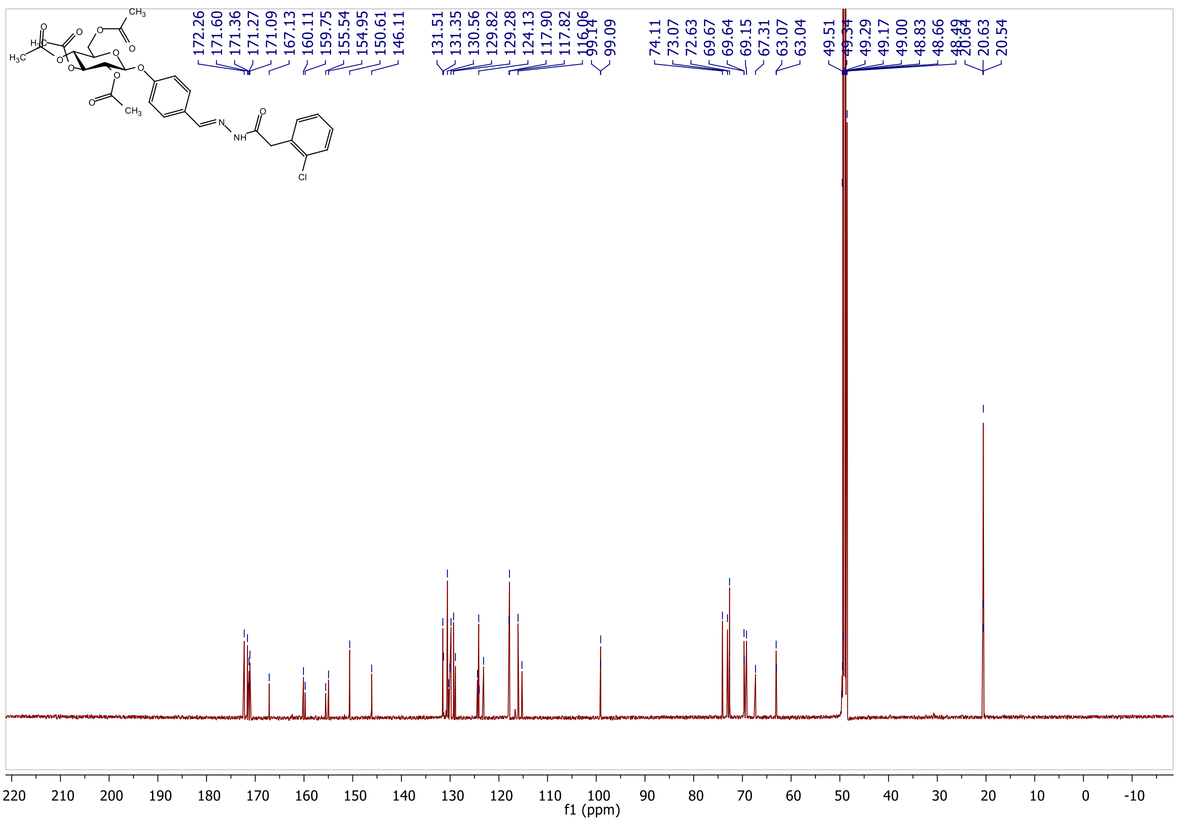

^{13}C-NMR spectrum of **acetylated A1H12**

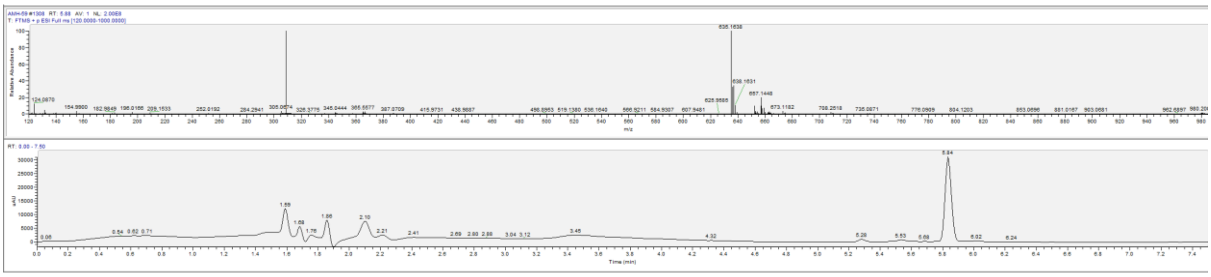

HPLC-HRMS analysis of **acetylated A1H12**, > 95% pure.

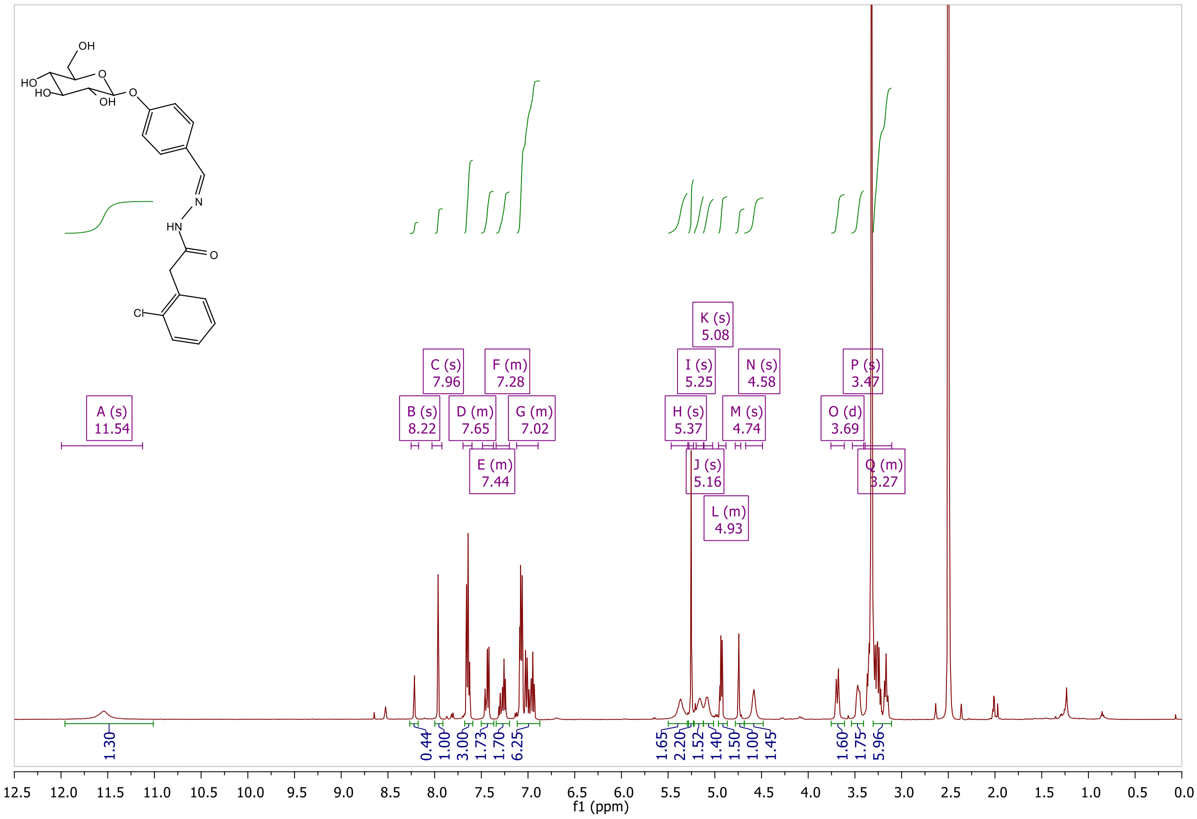

^{1}H-NMR spectrum of **A1H12**

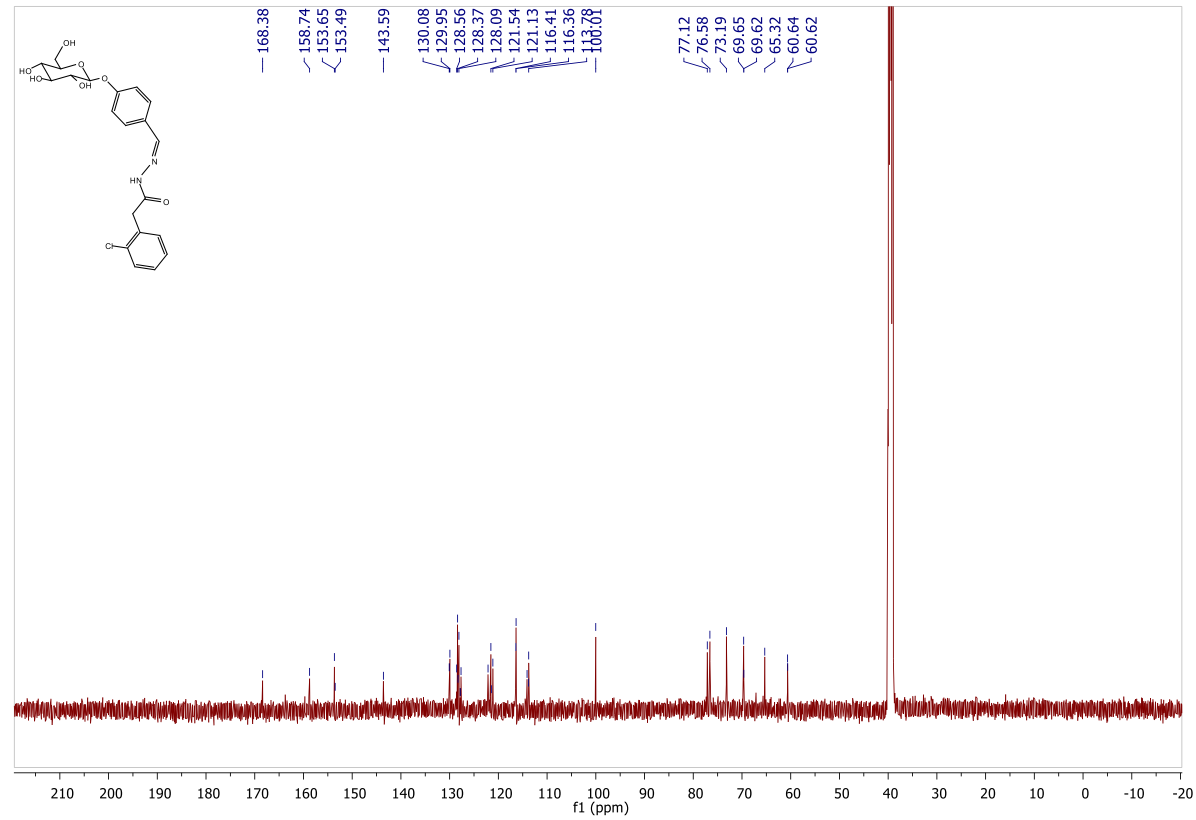

^{13}C-NMR spectrum of **A1H12**

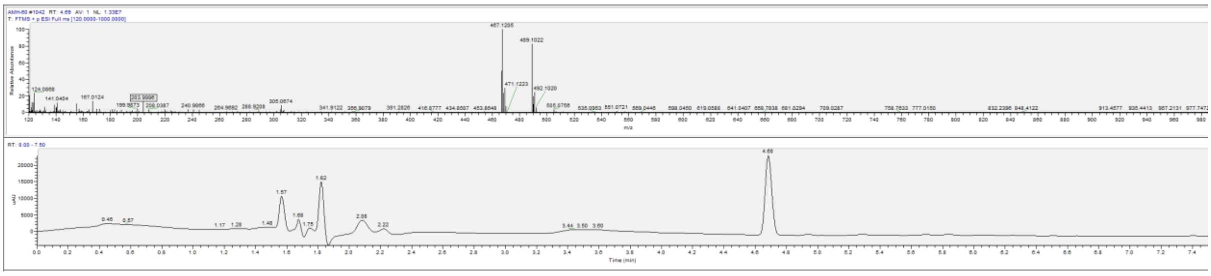

HPLC-HRMS analysis of **A1H12**, > 95% pure.

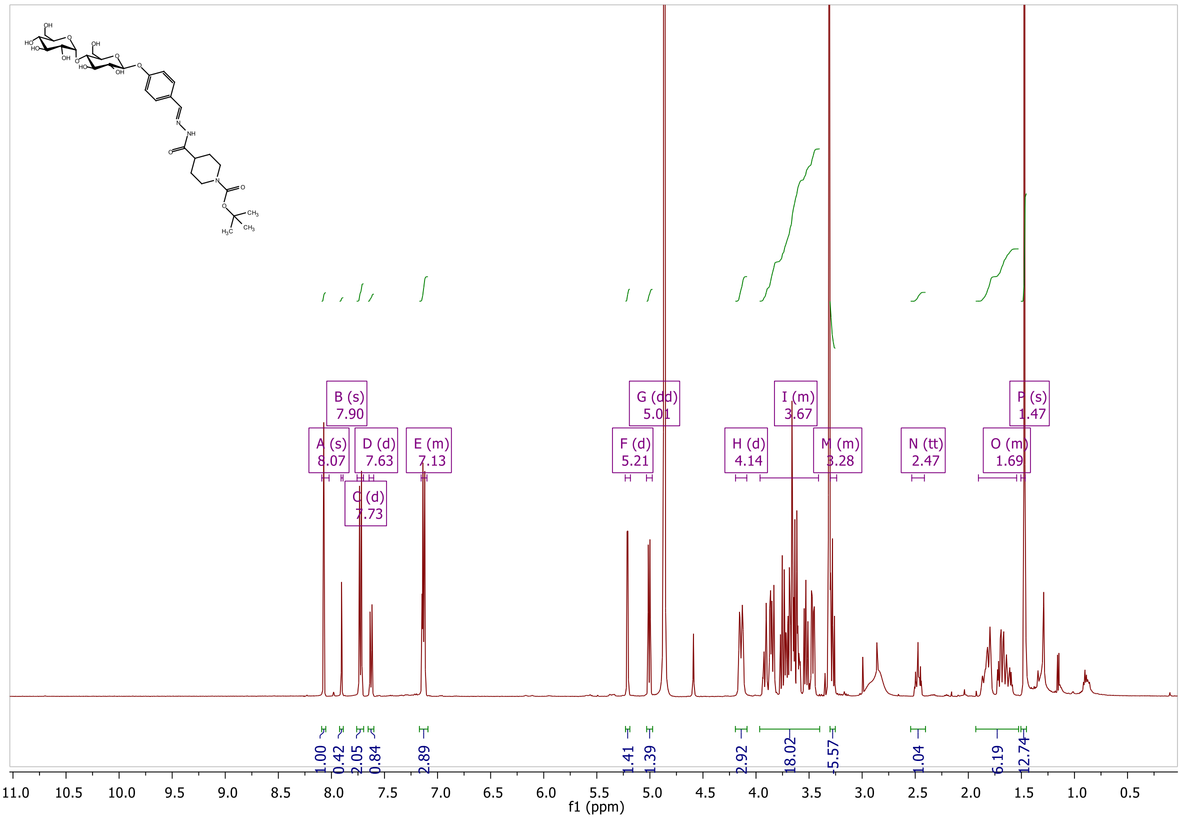

^{1}H-NMR spectrum of **A2H2**

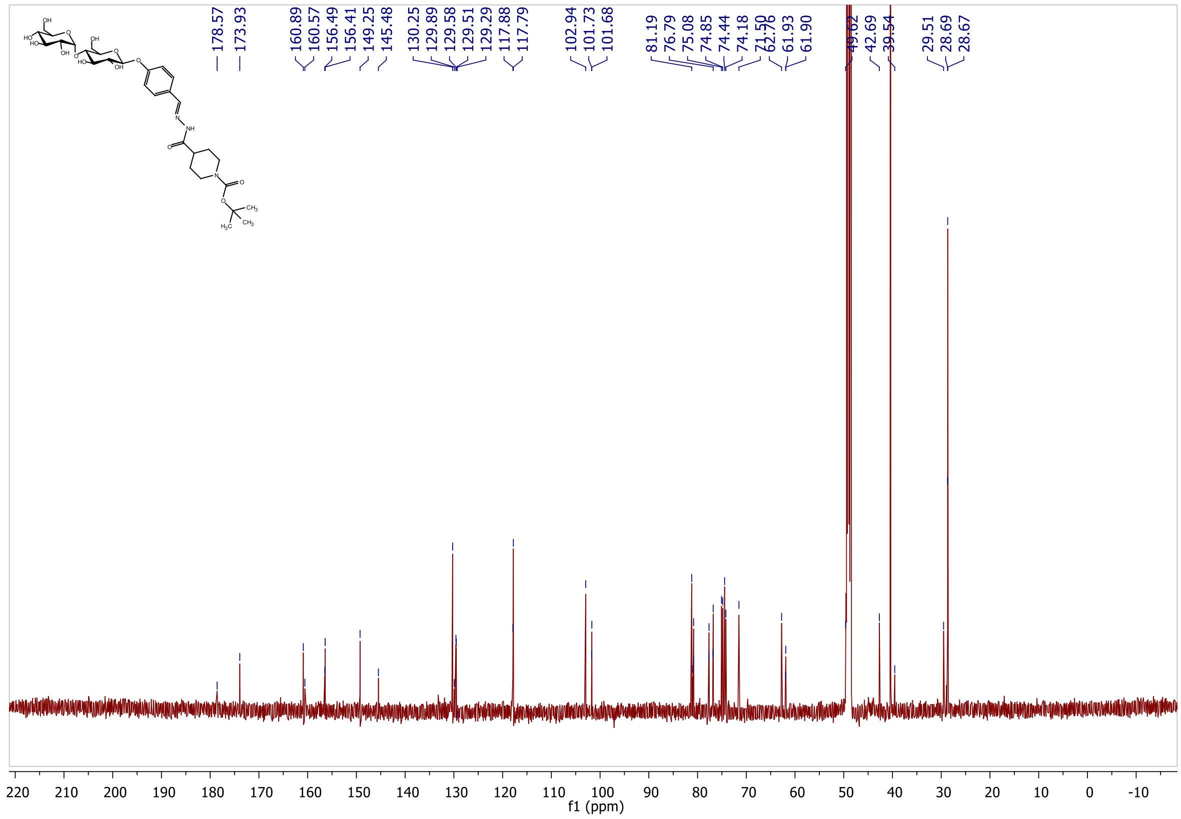

^{13}C-NMR spectrum of **A2H2**

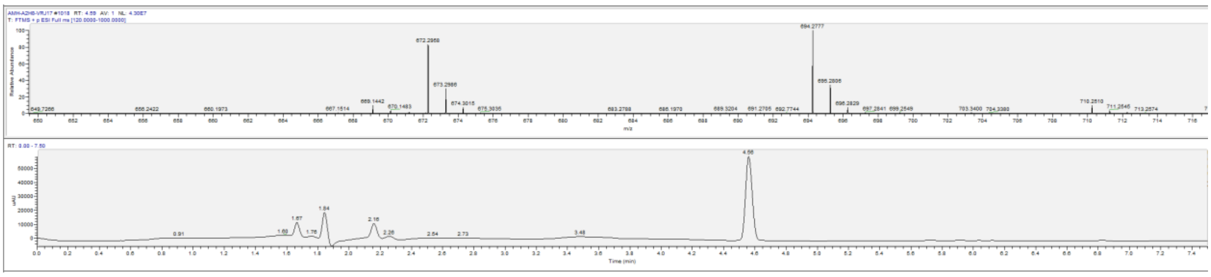

HPLC-HRMS analysis of **A2H2**, > 95% pure.

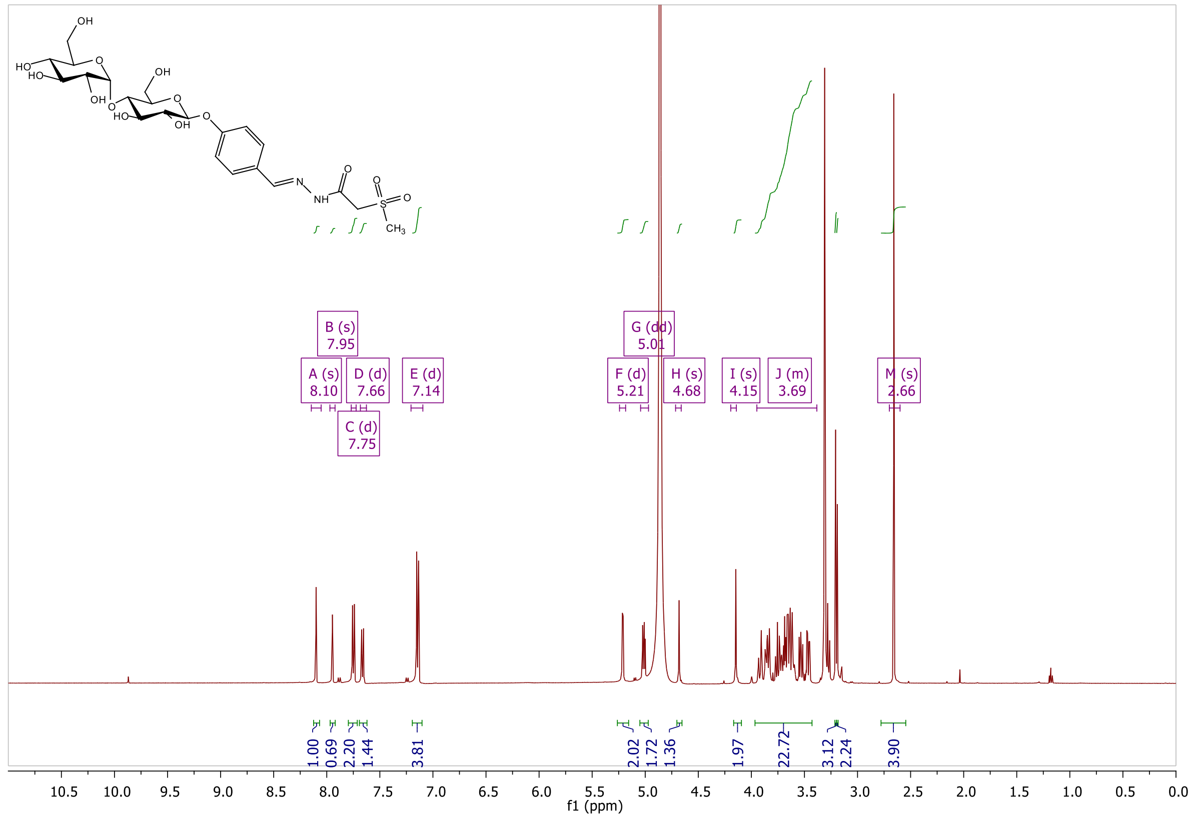

^{1}H-NMR spectrum of **A2H6**

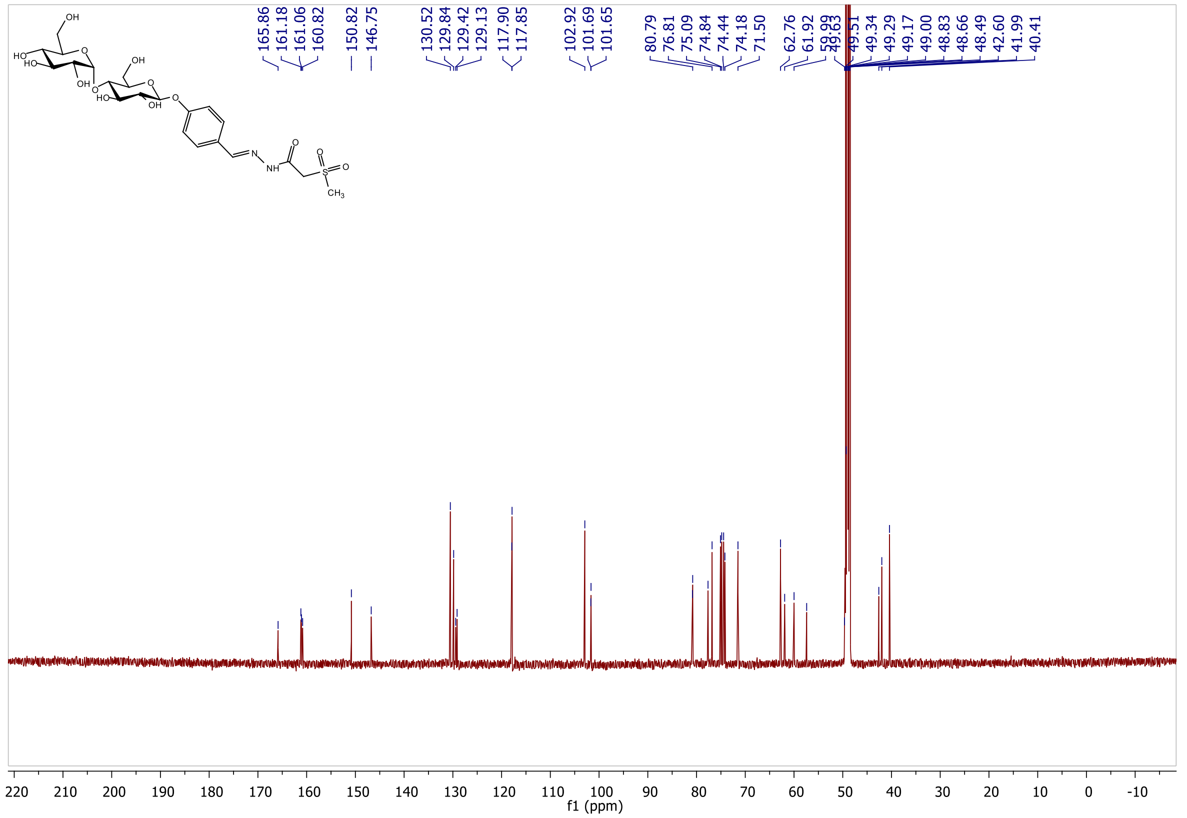

^{13}C-NMR spectrum of **A2H6**

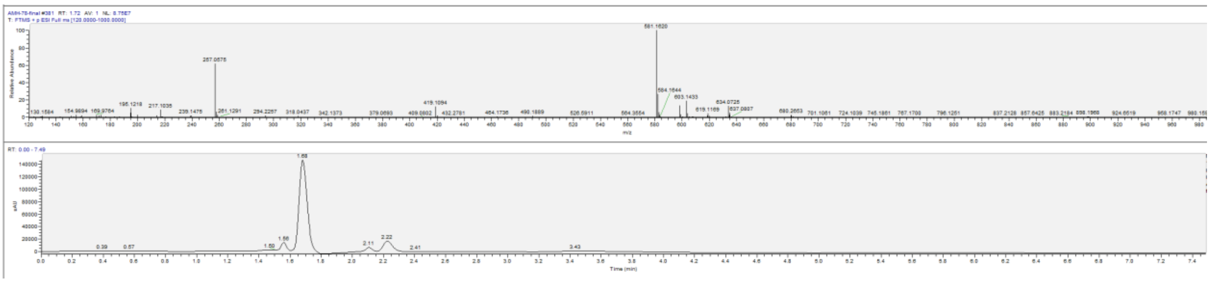

HPLC-HRMS analysis of **A2H6**, > 95% pure.

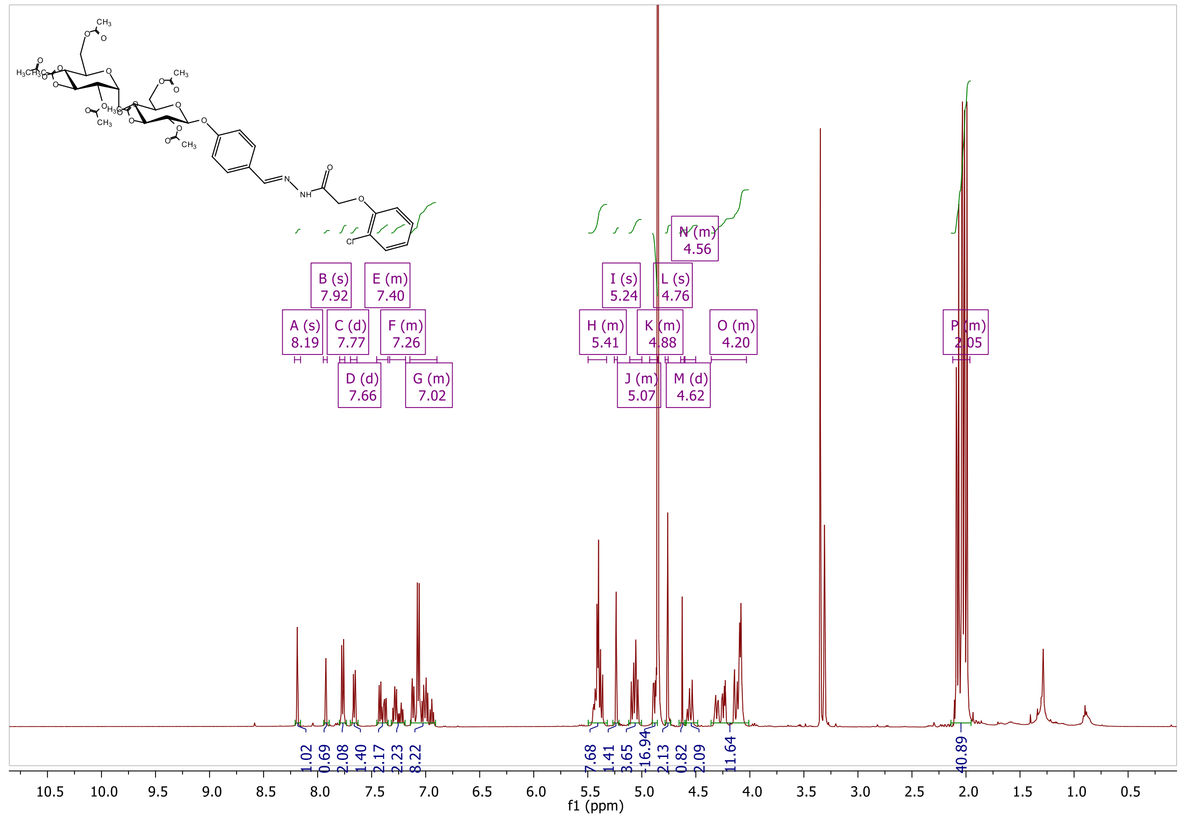

^{1}H-NMR spectrum of **acetylated A2H12**

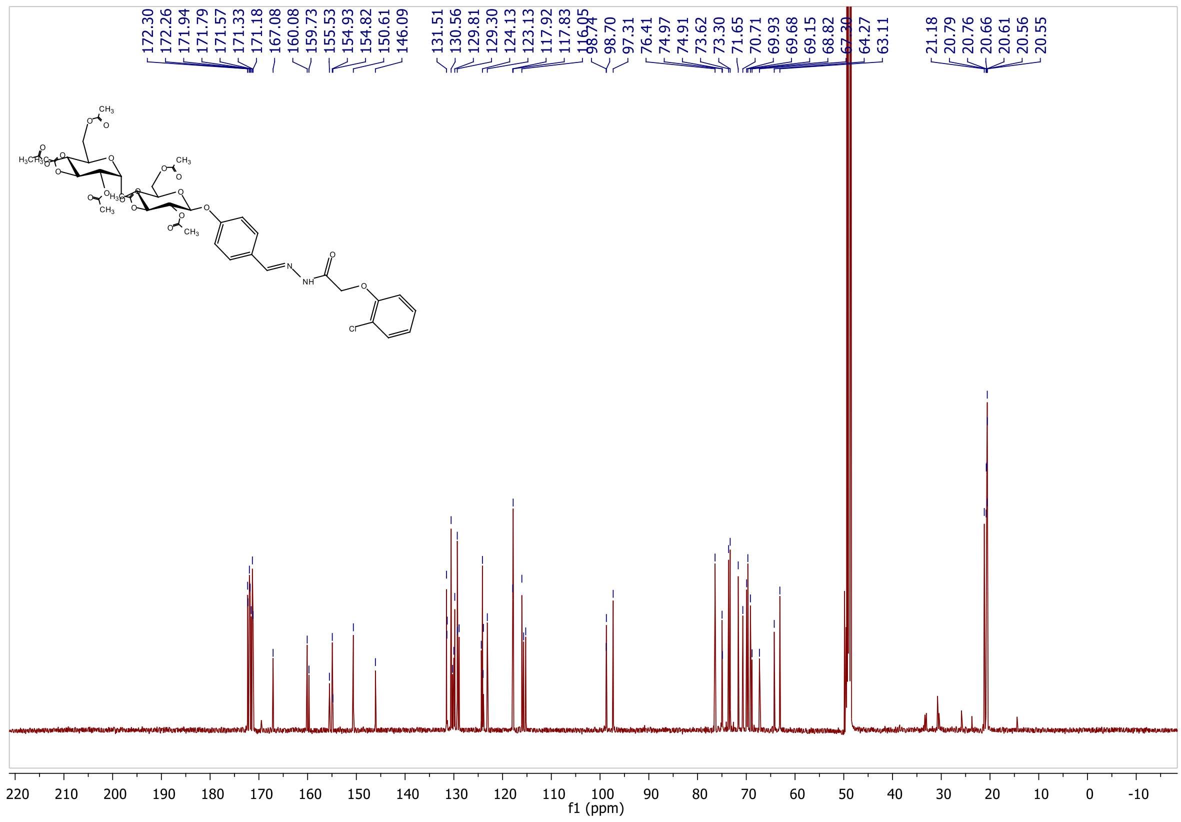

^{13}C-NMR spectrum of **acetylated A2H12**

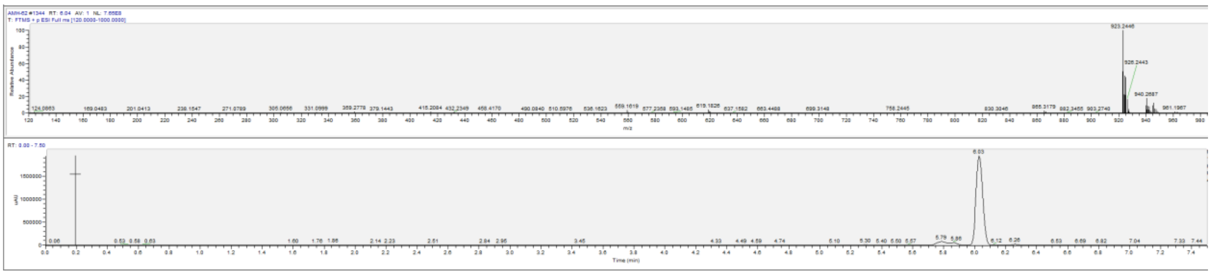

HPLC-HRMS analysis of **acetylated A2H12**, > 95% pure.

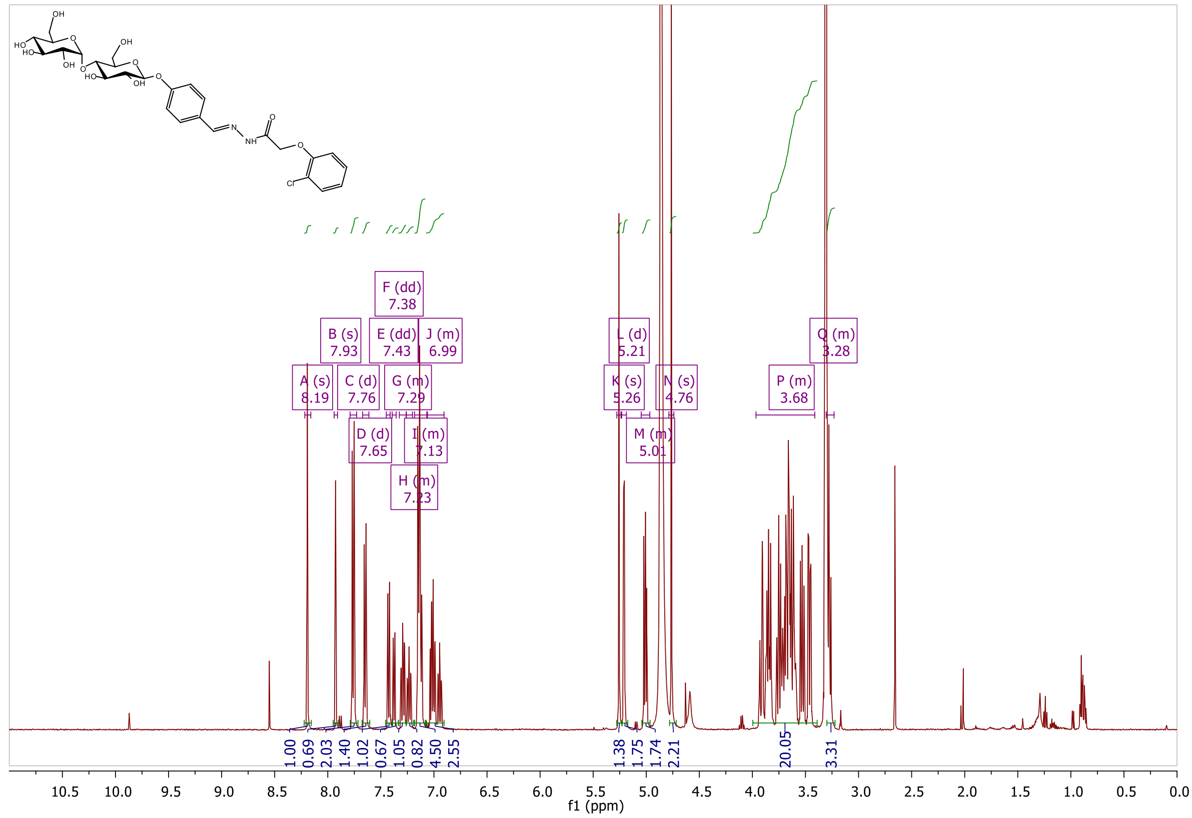

^{1}H-NMR spectrum of **A2H12**

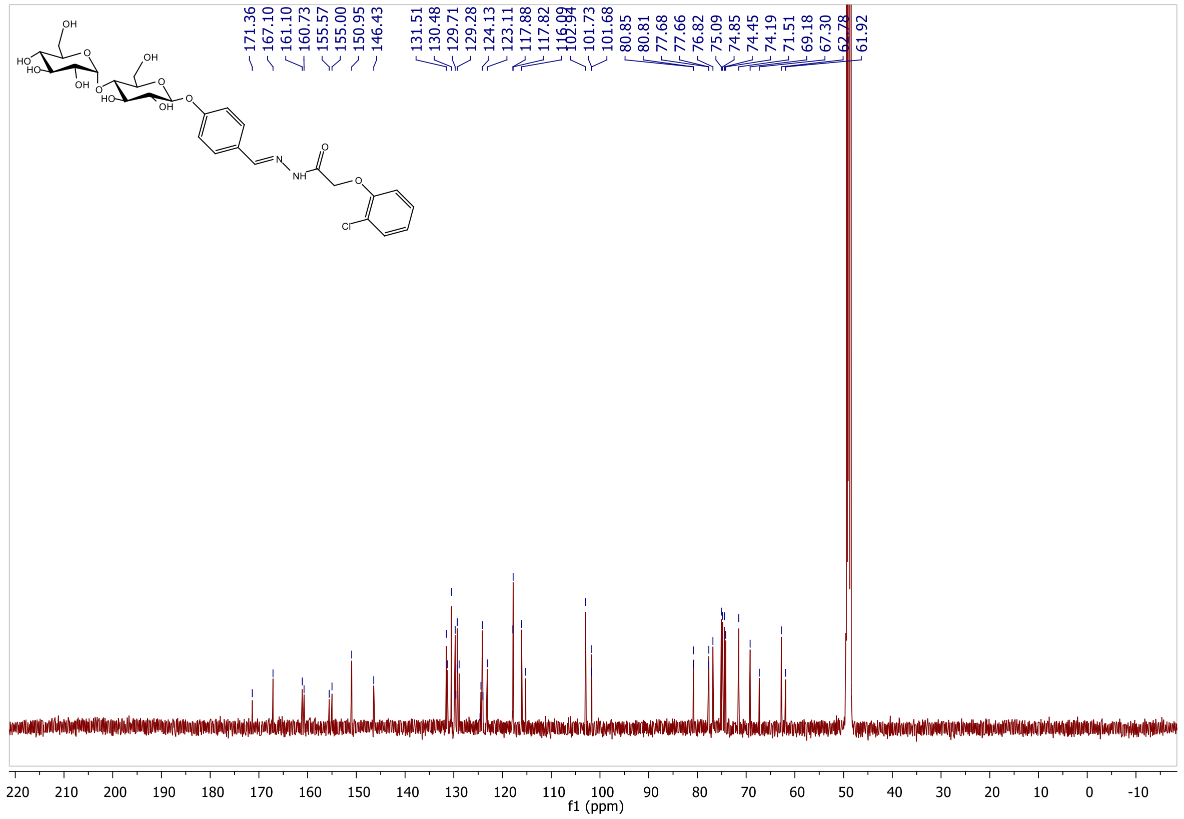

^{13}C-NMR spectrum of **A2H12**

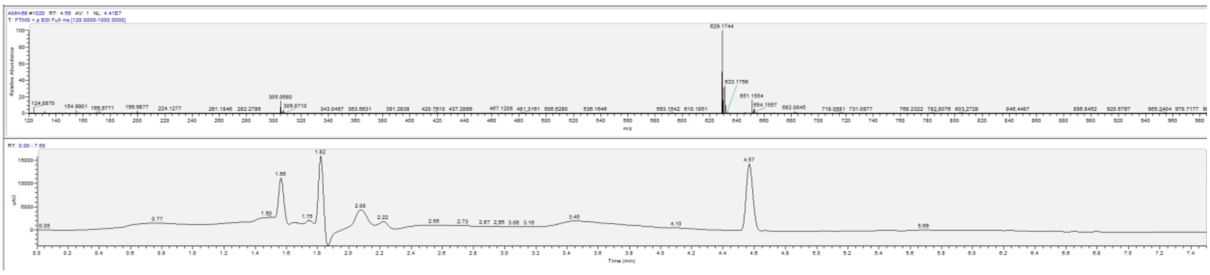

HPLC-HRMS analysis of **A2H12**, > 95% pure.
